# Supplementary material for: Complete mitochondrial genome of Episymploce splendens (Blattodea: Ectobiidae): A large intergenic spacer and lacking of two tRNA genes
Source: PLoS One. 2022 Jun 2;17(6):e0268064. doi: 10.1371/journal.pone.0268064 (PMC9162313; doi:10.1371/journal.pone.0268064)
Supplement: S1 Table — (DOCX) [file pone.0268064.s001.docx]

| Order | Species | GenBank Accession number |
| --- | --- | --- |
| Blattodea | *Panchlora nivea* | NC_030002.1 |
|  | *Opisthoplatia orientalis* | NC_029225.1 |
|  | *Gromphadorhina portentosa* | NC_030001.1 |
|  | *Nauphoeta cinerea* | NC_035052.1 |
|  | *Blaptica dubia* | NC_029224.1 |
|  | *Eupolyphaga sinensis* | NC_014274.1 |
|  | *Periplaneta fuliginosa* | NC_006076.1 |
|  | *Periplaneta australasiae* | NC_034841.1 |
|  | *Periplaneta americana* | NC_016956.1 |
|  | *Periplaneta brunnea* | MG010455 |
|  | *Shelfordella lateralis* | NC_030003.1 |
|  | *Neostylopyga rhombifolia* | NC_034842.1 |
|  | *Cryptocercus relictus* | NC_018132.1 |
|  | *Cryptocercus kyebangensis* | NC_030191.1 |
|  | *Blattella germanica* | NC_012901.1 |
|  | *Blattella bisignata* | NC_018549.1 |
|  | *Cryptocercus meridianus* | NC_037496.1 |
|  | *Episymploce splendens* | OK094023 |
|  | *Episymploce splendens-NUMT-1* |  |
|  | *Episymploce splendens-NUMT-2* |  |
| Isoptera | *Mastotermes darwiniensis* | NC_018120.1 |
|  | *Neocapritermes taracua* | NC_026116.1 |
|  | *Termes comis* | NC_034121.1 |
| Lepidoptera  (Outgroup) | *Papilio protenor* | NC_034317.1 |
|  | *Biston panterinaria* | NC_020004.1 |
| Mantodea | *Creobroter gemmatus* | NC_030267.1 |
|  | *Humbertiella nada* | NC_030264.1 |

**S1 Table. GenBank accession numbers for divergence date analyses.**
