# Supplementary material for: Complete mitochondrial genome of Episymploce splendens (Blattodea: Ectobiidae): A large intergenic spacer and lacking of two tRNA genes
Source: PLoS One. 2022 Jun 2;17(6):e0268064. doi: 10.1371/journal.pone.0268064 (PMC9162313; doi:10.1371/journal.pone.0268064)
Supplement: S2 Table — (DOCX) [file pone.0268064.s002.docx]

**S2 Table. Nucleotide composition in different regions of *Episymploce splendens.***

| **Gene region** | **A/%** | **T/%** | **C/%** | **G/%** | **A+T/%** | **G+C/%** | **AT-Skew** | **GC-Skew** |
| --- | --- | --- | --- | --- | --- | --- | --- | --- |
| whole mitogenome | 37.8 | 36.8 | 14 | 11.4 | 74.6 | 25.4 | 0.013 | -0.102 |
| PCGs | 32.1 | 42.5 | 12.2 | 13.3 | 74.6 | 25.4 | -0.139 | 0.043 |
| tRNAs | 38.6 | 37.4 | 14.2 | 9.8 | 76 | 24 | 0.016 | -0.183 |
| rRNAs | 33.7 | 42.1 | 7.9 | 16.2 | 75.8 | 24.2 | -0.111 | 0.343 |
| AT-rich region | 33.4 | 31.4 | 29.3 | 5.9 | 64.8 | 35.2 | 0.031 | -0.665 |
