# Supplementary material for: Complete mitochondrial genome of Episymploce splendens (Blattodea: Ectobiidae): A large intergenic spacer and lacking of two tRNA genes
Source: PLoS One. 2022 Jun 2;17(6):e0268064. doi: 10.1371/journal.pone.0268064 (PMC9162313; doi:10.1371/journal.pone.0268064)
Supplement: S3 Table — (DOCX) [file pone.0268064.s003.docx]

**S3 Table. Relative synonymous codon usage (RSCU) for PCGs of** ***Episymploce splendens.***

| **Amino** | **Codon** | **Count** | **RSCU** | **Amino** | **Codon** | **Count** | **RSCU** | **Amino** | **Codon** | **Count** | **RSCU** | **Amino** | **Codon** | **Count** | **RSCU** |
| --- | --- | --- | --- | --- | --- | --- | --- | --- | --- | --- | --- | --- | --- | --- | --- |
| Phe | UUU(F) | 279 | 1.74 | Ser* | UCU(S) | 102 | 2.29 | Tyr | UAU(Y) | 147 | 1.73 | Cys | UGU(C) | 42 | 1.91 |
|  | UUC(F) | 41 | 0.26 |  | UCC(S) | 13 | 0.29 |  | UAC(Y) | 23 | 0.27 |  | UGC(C) | 2 | 0.09 |
| Leu* | UUA(L) | 420 | 4.41 |  | UCA(S) | 115 | 2.58 | Stop | UAA(*) | 0 | 0 | Trp | UGA(W) | 94 | 1.81 |
|  | UUG(L) | 67 | 0.7 |  | UCG(S) | 3 | 0.07 |  | UAG(*) | 0 | 0 |  | UGG(W) | 10 | 0.19 |
| Leu | CUU(L) | 30 | 0.31 | Pro | CCU(P) | 68 | 1.97 | His | CAU(H) | 61 | 1.69 | Arg | CGU(R) | 16 | 1.1 |
|  | CUC(L) | 6 | 0.06 |  | CCC(P) | 9 | 0.26 |  | CAC(H) | 11 | 0.31 |  | CGC(R) | 2 | 0.14 |
|  | CUA(L) | 46 | 0.48 |  | CCA(P) | 60 | 1.74 | Gln | CAA(Q) | 66 | 1.86 |  | CGA(R) | 37 | 2.55 |
|  | CUG(L) | 3 | 0.03 |  | CCG(P) | 1 | 0.03 |  | CAG(Q) | 5 | 0.14 |  | CGG(R) | 3 | 0.21 |
| Ile | AUU(I) | 333 | 1.86 | Thr | ACU(T) | 78 | 1.72 | Asn | AAU(N) | 148 | 1.74 | Ser | AGU(S) | 37 | 0.83 |
|  | AUC(I) | 26 | 0.14 |  | ACC(T) | 15 | 0.33 |  | AAC(N) | 22 | 0.26 |  | AGC(S) | 3 | 0.07 |
| Met | AUA(M) | 224 | 1.8 |  | ACA(T) | 82 | 1.81 | Lys | AAA(K) | 75 | 1.67 |  | AGA(S) | 83 | 1.87 |
|  | AUG(M) | 25 | 0.2 |  | ACG(T) | 6 | 0.13 |  | AAG(K) | 15 | 0.33 |  | AGG(S) | 0 | 0 |
| Val | GUU(V) | 99 | 1.99 | Ala | GCU(A) | 93 | 2.09 | Asp | GAU(D) | 62 | 1.8 | Gly | GGU(G) | 76 | 1.35 |
|  | GUC(V) | 4 | 0.08 |  | GCC(A) | 14 | 0.31 |  | GAC(D) | 7 | 0.2 |  | GGC(G) | 9 | 0.16 |
|  | GUA(V) | 86 | 1.73 |  | GCA(A) | 68 | 1.53 | Glu | GAA(E) | 79 | 1.84 |  | GGA(G) | 124 | 2.2 |
|  | GUG(V) | 10 | 0.2 |  | GCG(A) | 3 | 0.07 |  | GAG(E) | 7 | 0.16 |  | GGG(G) | 16 | 0.28 |

L, L*, S and S* indicate tRNA-Leu(CUN), tRNA-Leu(UUR), tRNA-Ser(AGN), and tRNA-Ser(UCN), respectively.
