# Supplementary material for: Complete mitochondrial genome of Episymploce splendens (Blattodea: Ectobiidae): A large intergenic spacer and lacking of two tRNA genes
Source: PLoS One. 2022 Jun 2;17(6):e0268064. doi: 10.1371/journal.pone.0268064 (PMC9162313; doi:10.1371/journal.pone.0268064)
Supplement: S4 Table — The similarity was calculated by DNAMAN. (DOCX) [file pone.0268064.s004.docx]

**S4 Table. The length and similarity of these large intergenic spacers with their original genes in some insects.** The similarity was calculated by DNAMAN.

| **Name** | **Original genes of mito-genome** | **Long intergenic region** | **Similarity** |
| --- | --- | --- | --- |
| Episymploce splendens | 113bp | 93bp | 64.6% |
| Phaenacantha marcida | 73bp | 72bp | 46.58% |
| Hycleus cichorii | 48bp | 39bp | 43.75% |
| Blaptica dubia | 142bp | 71bp | 22.54% |
| Hycleus phaleratus | 110bp | 62bp | 25.45% |
| Hycleus marcipoli | 112bp | 56bp | 23.68% |
