# Supplementary figures and images for: Complete mitochondrial genome of Episymploce splendens (Blattodea: Ectobiidae): A large intergenic spacer and lacking of two tRNA genes
Source: PLoS One. 2022 Jun 2;17(6):e0268064. doi: 10.1371/journal.pone.0268064 (PMC9162313; doi:10.1371/journal.pone.0268064)

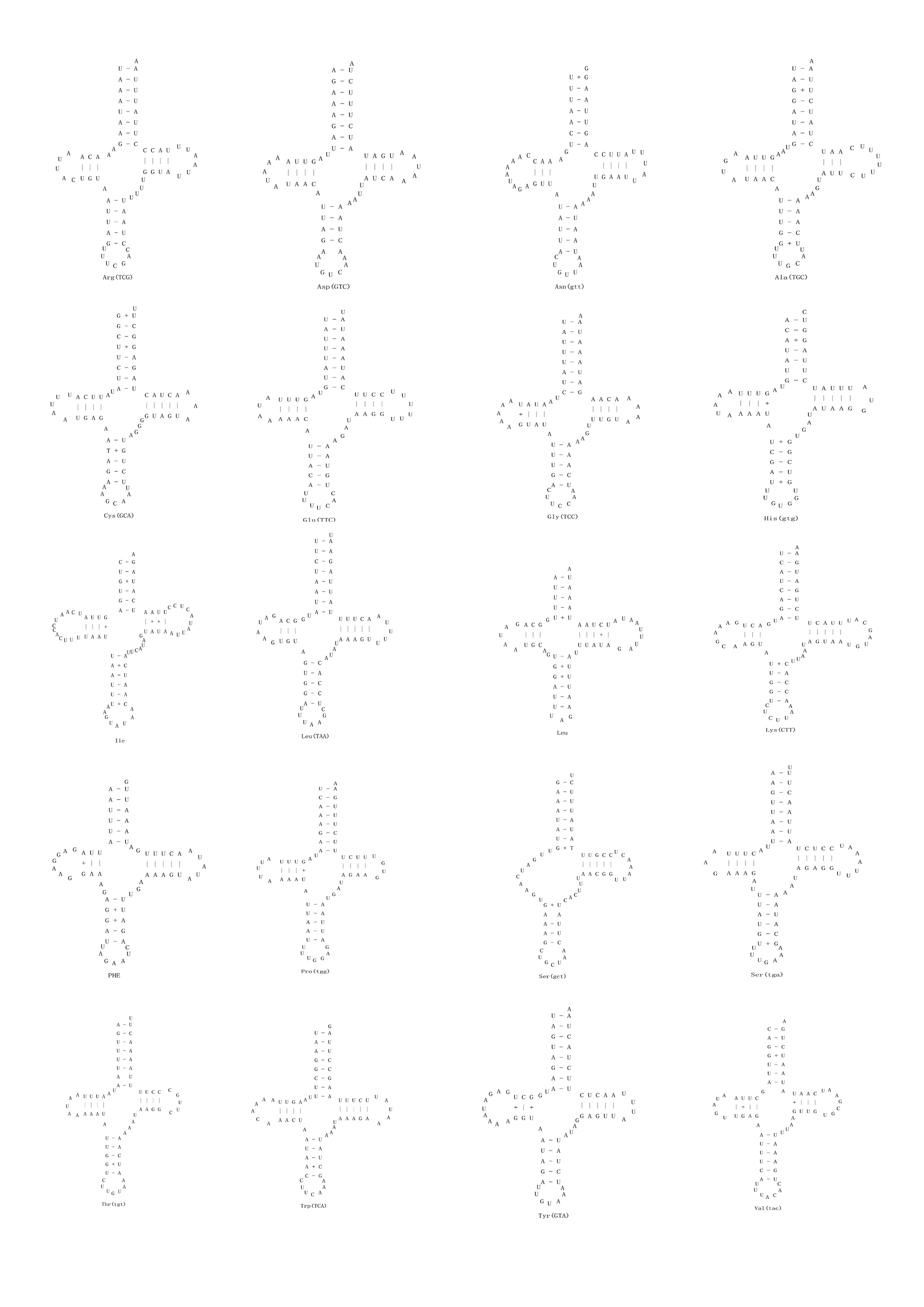

Supplement: S1 Fig — (TIF) [file pone.0268064.s005.tif]

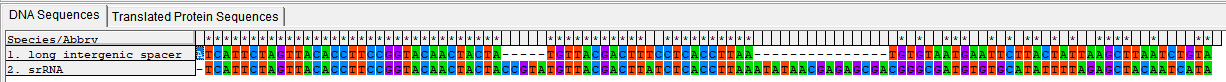

Supplement: S2 Fig — (TIF) [file pone.0268064.s006.tif]

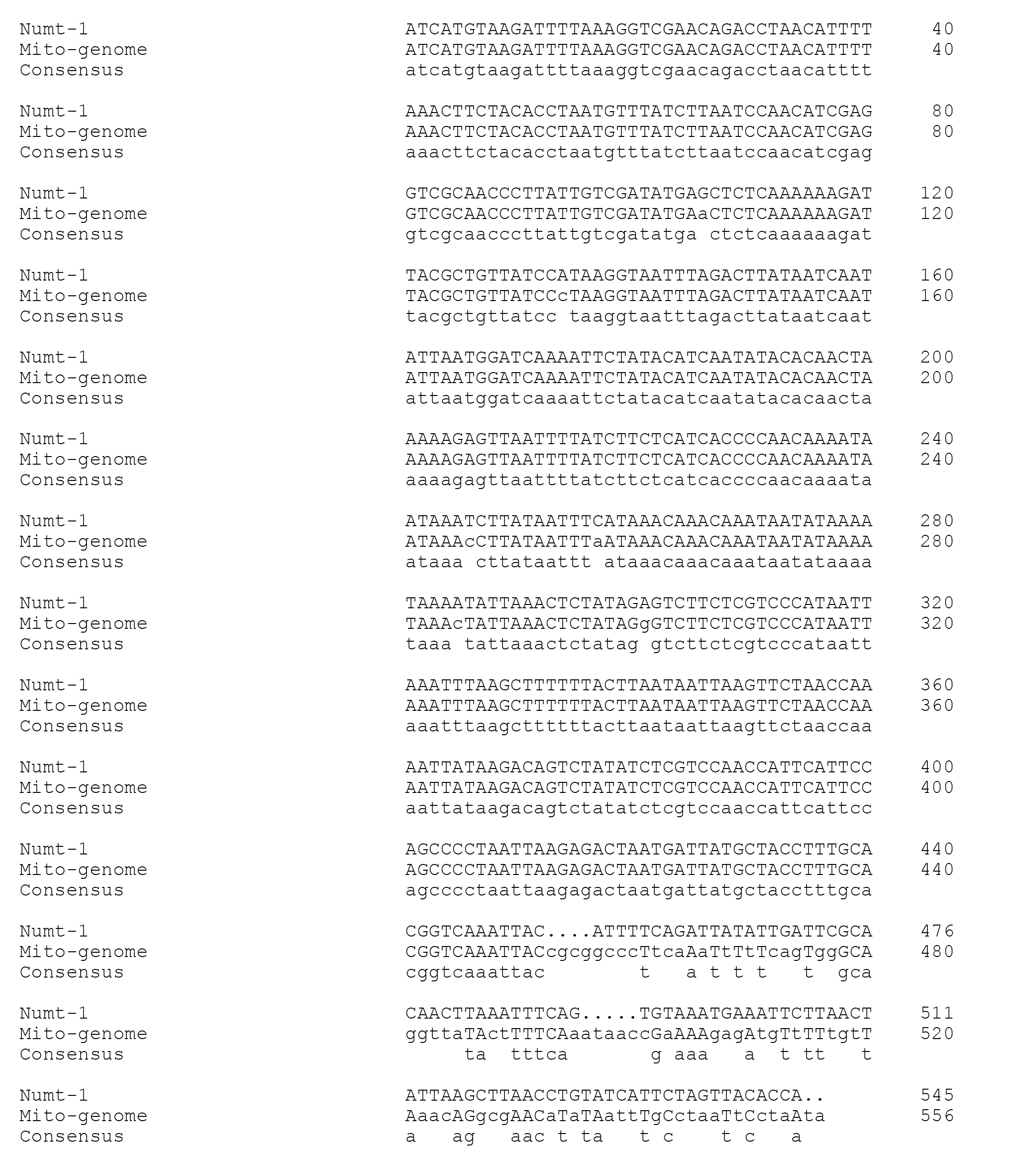

Supplement: S3 Fig — (TIF) [file pone.0268064.s007.tif]

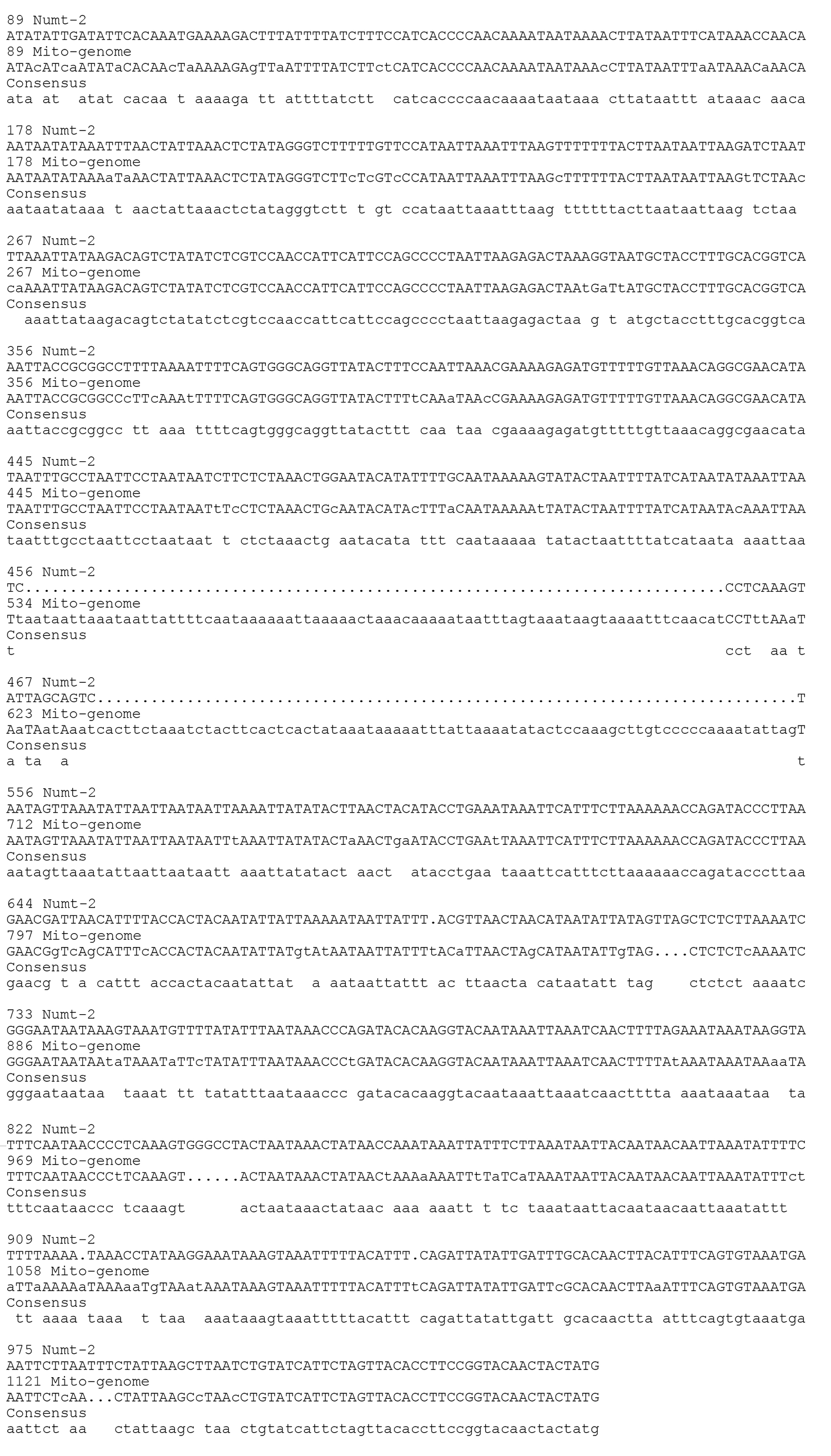

Supplement: S4 Fig — (TIF) [file pone.0268064.s008.tif]
