# Supplementary material for: Complete mitochondrial genome of Episymploce splendens (Blattodea: Ectobiidae): A large intergenic spacer and lacking of two tRNA genes
Source: PLoS One. 2022 Jun 2;17(6):e0268064. doi: 10.1371/journal.pone.0268064 (PMC9162313; doi:10.1371/journal.pone.0268064)
Supplement: Attachment — Submitted filename: PONE-D-21-15397_reviewer copie.pdf [file pone.0268064.s009.pdf]

PLOS ONE

Complete mitochondrial genome of Episymploce splendens (Blattodea: Ectobiidae): a large intergenic spacer and lacking of two tRNA genes

--Manuscript Draft--

**Manuscript Number:**	PONE-D-21-15397
**Article Type:**	Research Article
**Full Title:**	Complete mitochondrial genome of Episymploce splendens (Blattodea: Ectobiidae): a large intergenic spacer and lacking of two tRNA genes
**Short Title:**	Complete mitochondrial genome of Episymploce splendens (Blattodea: Ectobiidae)
**Corresponding Author:**	Xiuyue Zhang<br>Sichuan University<br>Chengdu, CHINA
**Keywords:**	Episymploce splendens, mito-genome, tRNAs lacking, Large intergenic spacer, Numts, Phylogenetic analysis
**Abstract:**	The complete mitochondrial genome of Episymploce splendens, 15,802 bp in length, was determined and used to reconstruct the phylogenetic relationships within Blattodea in this study. The mito-genome included 13 PCGs, 20 tRNAs and 2 rRNAs. Unlike most typical mito-genomes with conservative gene arrangement and exceptional economic organization, E. splendens mito-genome has two tRNAs (tRNA-Gln and tRNA-Met) absence and a long intergenic spacer sequence (93 bp) between tRNA-Val and srRNA, showing the diversified features of insect mito-genomes. This is the first report of the tRNAs deletion in Orthopterodea mito-genome and we also proposed the duplication/random loss model as the origin mechanism of the long intergenic spacer. Two Numts，Numt-1 (557 bp) and Numt-2 (975 bp) transferred to the nucleus at about 13.45 Ma to 57.4 Ma, and 4.36 Ma to 31.66 Ma respectively, were found in E. splenden. They can be used as molecular fossils in insect phylogenetic relationship inference. The phylogenetic relationships were analyzed based on the amino acid and nucleotide sequences of 13 PCGs using maximum likelihood and Bayesian inference and a similar topology was yielded, except for the position of Caelifera and Eupolyphaga sinensis (Corydiidae). Tree results supported that (Blattidae + (Corydiidae + (Cryptocercidae + Isoptera))) and Blaberoidea being placed in the basal position of Blattodea, Isoptera should be classified as a family within Blattodea and Mantodea as the sister clade to (Isoptera + Blattodea). Our study provided important data for further studies on insect mito-genome evolution and the phylogenetic relationships of Blattodea.
**Order of Authors:**	Lin Yan
	Zhenzhen Hou
	Jinnan Ma
	Hongmei Wang
	Jie Gao
	Chenjuan Zeng
	Qin Chen
	Bisong Yue
	Xiuyue Zhang
**Additional Information:**	
**Question**	**Response**

Powered by Editorial Manager® and ProduXion Manager® from Aries Systems Corporation

**Financial Disclosure**<br><br>Enter a financial disclosure statement that describes the sources of funding for the work included in this submission. Review the submission guidelines for detailed requirements. View published research articles from *PLOS ONE* for specific examples.<br><br>This statement is required for submission and **will appear in the published article** if the submission is accepted. Please make sure it is accurate.<br><br>**Unfunded studies**<br>Enter: *The author(s) received no specific funding for this work.*<br><br>**Funded studies**<br>Enter a statement with the following details:<br>• Initials of the authors who received each award<br>• Grant numbers awarded to each author<br>• The full name of each funder<br>• URL of each funder website<br>• Did the sponsors or funders play any role in the study design, data collection and analysis, decision to publish, or preparation of the manuscript?<br>• **NO** - Include this sentence at the end of your statement: *The funders had no role in study design, data collection and analysis, decision to publish, or preparation of the manuscript.*<br>• **YES** - Specify the role(s) played.<br><br>* typeset	This study was funded by the Science and Technology Project of Sichuan Province (2017SZ0019), Special funds for central government to guide local scientific and Technological Development (2020ZYD098) and State Administration of Traditional Chinese Medicine of the P.R.C (ZYBZH-C-SC-53).
**Competing Interests**<br><br>Use the instructions below to enter a competing interest statement for this submission. On behalf of all authors, disclose any competing interests that could be perceived to bias this work—acknowledging all financial support and any other relevant financial or non-financial competing interests.<br><br>This statement is required for submission	The authors have declared that no competing interests exist.

Powered by Editorial Manager® and ProduXion Manager® from Aries Systems Corporation

and **will appear in the published article** if the submission is accepted. Please make sure it is accurate and that any funding sources listed in your Funding Information later in the submission form are also declared in your Financial Disclosure statement.<br><br>View published research articles from *PLOS ONE* for specific examples.<br><br>**NO authors have competing interests**<br><br>Enter: *The authors have declared that no competing interests exist.*<br><br>**Authors with competing interests**<br><br>Enter competing interest details beginning with this statement:<br><br>*I have read the journal's policy and the authors of this manuscript have the following competing interests: [insert competing interests here]*<br><br>* typeset	
**Ethics Statement**<br><br>Enter an ethics statement for this submission. This statement is required if the study involved:<br><br>• Human participants<br>• Human specimens or tissue<br>• Vertebrate animals or cephalopods<br>• Vertebrate embryos or tissues<br>• Field research<br><br>Write "N/A" if the submission does not require an ethics statement.<br><br>General guidance is provided below. Consult the submission guidelines for detailed instructions. **Make sure that all information entered here is included in the Methods section of the manuscript.**	Episymploce splendens is a cockroach. It's not a vertebrate, and it's not a protectanimal. We stun the insects with ether and then put them into anhydrous ethanol to die, and take fresh materials for subsequent experiments.

**Format for specific study types**<br><br>**Human Subject Research (involving human participants and/or tissue)**<br>• Give the name of the institutional review board or ethics committee that approved the study<br>• Include the approval number and/or a statement indicating approval of this research<br>• Indicate the form of consent obtained (written/oral) or the reason that consent was not obtained (e.g. the data were analyzed anonymously)<br><br>**Animal Research (involving vertebrate animals, embryos or tissues)**<br>• Provide the name of the Institutional Animal Care and Use Committee (IACUC) or other relevant ethics board that reviewed the study protocol, and indicate whether they approved this research or granted a formal waiver of ethical approval<br>• Include an approval number if one was obtained<br>• If the study involved *non-human primates*, add *additional details* about animal welfare and steps taken to ameliorate suffering<br>• If anesthesia, euthanasia, or any kind of animal sacrifice is part of the study, include briefly which substances and/or methods were applied<br><br>**Field Research**<br><br>Include the following details if this study involves the collection of plant, animal, or other materials from a natural setting:<br>• Field permit number<br>• Name of the institution or relevant body that granted permission	
**Data Availability**<br><br>Authors are required to make all data underlying the findings described fully available, without restriction, and from the time of publication. PLOS allows rare exceptions to address legal and ethical concerns. See the PLOS Data Policy and FAQ for detailed information.	Yes - all data are fully available without restriction

Powered by Editorial Manager® and ProduXion Manager® from Aries Systems Corporation

A Data Availability Statement describing where the data can be found is required at submission. Your answers to this question constitute the Data Availability Statement and **will be published in the article**, if accepted.<br><br>**Important:** Stating 'data available on request from the author' is not sufficient. If your data are only available upon request, select 'No' for the first question and explain your exceptional situation in the text box.<br><br>Do the authors confirm that all data underlying the findings described in their manuscript are fully available without restriction?	
**Describe where the data may be found in full sentences. If you are copying our sample text, replace any instances of XXX with the appropriate details.**<br><br>• If the data are **held or will be held in a public repository**, include URLs, accession numbers or DOIs. If this information will only be available after acceptance, indicate this by ticking the box below. For example: *All XXX files are available from the XXX database (accession number(s) XXX, XXX.).*<br>• If the data are all contained **within the manuscript and/or Supporting Information files**, enter the following: *All relevant data are within the manuscript and its Supporting Information files.*<br>• If neither of these applies but you are able to provide **details of access elsewhere**, with or without limitations, please do so. For example:<br><br>*Data cannot be shared publicly because of [XXX]. Data are available from the XXX Institutional Data Access / Ethics Committee (contact via XXX) for researchers who meet the criteria for access to confidential data.*<br><br>*The data underlying the results presented in the study are available from (include the name of the third party*	All sequences are awaited to upload to NCBI Genbank database.

and contact information or URL).<br>• This text is appropriate if the data are owned by a third party and authors do not have permission to share the data.<br><br>* typeset	
Additional data availability information:	

Manuscript Click here to access/download;Manuscript;Text.docx

Complete mitochondrial genome of *Episymploce splendens* (Blattodea: Ectobiidae): a large intergenic spacer and lacking of two tRNA genes

Lin Yan[1], Zhenzhen Hou[2], Jinnan Ma[1], Hongmei Wang[1], Jie Gao[3], Chenjuan Zeng[3], Qin Chen[1], Bisong Yue[1], Xiuyue Zhang[1,2]*

1Key Laboratory of Bio-resources and Eco-environment, Ministry of Education, College of Life Sciences, Sichuan University, No.24 South Section 1, Yihuan Road, Chengdu, China, 610065.

2Sichuan Key Laboratory of Conservation Biology on Endangered Wildlife, College of Life Sciences, Sichuan University, No.24 South Section 1, Yihuan Road, Chengdu, China, 610065.

3Sichuan Key Laboratory of Medicinal American Cockroach, Xichang, Sichuan Province 615000, China.

*E-mail: zhangxiuyue@scu.edu.cn

Abstract

The complete mitochondrial genome of *Episymploce splendens*, 15,802 bp in length, was determined and used to reconstruct the phylogenetic relationships within Blattodea in this study. The mito-genome included 13 PCGs, 20 tRNAs and 2 rRNAs. Unlike most typical mito-genomes with conservative gene arrangement and exceptional economic organization, *E. splendens* mito-genome has two tRNAs (tRNA-Gln and tRNA-Met) absence and a long intergenic spacer sequence (93 bp) between tRNA-Val and srRNA, showing the diversified features of insect mito-genomes. This is the first report of the tRNAs deletion in Orthopterodea mito-genome and we also proposed the duplication/random loss model as the origin mechanism of the long intergenic spacer. Two Numts， Numt-1 (557 bp) and Numt-2 (975 bp) transferred to the nucleus at about 13.45 Ma to 57.4 Ma, and 4.36 Ma to 31.66 Ma respectively, were found in *E. splenden*. They can be used as molecular fossils in insect phylogenetic relationship inference. The phylogenetic relationships were analyzed based on the amino acid and nucleotide sequences of 13 PCGs using maximum likelihood and Bayesian inference and a similar topology was yielded, except for the position of Caelifera and *Eupolyphaga sinensis* (Corydiidae). Tree results supported that (Blattidae + (Corydiidae + (Cryptocercidae + Isoptera))) and Blaberoidea being placed in the basal position of Blattodea, Isoptera should be classified as a family within

Blattodea and Mantodea as the sister clade to (Isoptera + Blattodea). Our study provided important data for further studies on insect mito-genome evolution and the phylogenetic relationships of Blattodea.

Introduction

The mitochondrial genome has the characteristics of maternal inheritance, rare genetic recombination, faster evolution rate and conservative gene arrangement [1]. The mito-genome commonly displays exceptional economy of organization, with overlapping genes or small intergenic spacers [2]. A typical insect mito-genome, with a compact circular molecule, generally encodes a fixed set of 37 genes including 13 protein-coding genes (PCGs), 22 transfer RNA (tRNA) genes, two ribosomal RNA (rRNA) genes and a control region [3]. However, exceptions have been reported, such as diversity of COI gene initiation codons, long gene intergenic spacers, and different control regions with great variation [4] [5]. Thus, insect mitochondrial genomes are good models for researching mitochondrial genome evolution.

Cockroach is a broad common name of insects within the order Blattodea and cockroaches are an ancient, diverse and globally widespread group. Many species of cockroaches are global health pests and can carry pathogens of human diseases [6]. Although cockroach phylogenetic relationship studies are extensive, there remains debate regarding

interfamily phylogenetic relationships [7]. For example, Maekawa and Matsumoto placed Corydiidae in the basal position of Blattodea [8]. While, Inward *et al* suggested the clade (Corydiidae + Nocticolidae) was located in the basal position of cockroaches [9]. However, many studies proposed that there was a basal split between Blaberoidea and Blattodea [4] [7] [10] [11] [12].

Episymploce splendens belongs to Ectobiidae, *Episymploce* and at present, no complete mitochondrial sequences of *Episymploce* have been recorded with the NCBI database. Previous studies have mainly focused on the morphological features of *Episymploce* rather than molecular data [13]. In this study, we determined, annotated and described the complete mito-genome of *E. splendens* and reconstructed the phylogenetic relationship of Blattodea based on the amino acid and nucleotide sequences of 13 PCGs. We found several interesting aspects of this species' mito-genome, including tRNAs lacking, long intergenic spacers, and nuclear mtDNA (Numts). These discoveries could deepen our understanding of mitochondrial genome of insects, and increase the clarity of Blattodea phylogenetic relationships and contribute to the study of insect mito-genome evolution.

Materials and Methods

Sample and DNA extraction

The cockroaches used in this study were collected on Mount Emei, Sichuan Province, China. The fresh material was placed in absolute ethanol and stored it at -20 ℃. Total genomic DNA was extracted from the muscle tissue using TIANamp Genomic DNA kit (TIANGEN, Beijing, China) and was subjected to quality inspection by nucleic acid concentration analyzer and 1% agarose gel electrophoresis, and then stored at -20 ℃.

PCR amplification and sequencing

Primers were designed based on the conserved sequences of *Blattella germanica* and *B. bisignata*, and then specific primers were designed based on the amplified and sequenced sequences at both ends [10] [14]. Primer details are listed in Table 1. Primers Es3, Es9 and Es10 were obtained from Xiao *et al* [10]. PCR was conducted using 2×Taq PCR Mix (Innovagene, Chengdu, China) and performed on a PTC-100 thermal cycler (BioRad, Hercules, CA) with the following cycling conditions: an initial denaturation for 5 min at 94℃, followed by 35 cycles of denaturation for 30s at 94℃, annealing for 30s at 50-62℃ (depending on primer combinations), elongation for 1-4 min (depending on putative length of the fragments) at 72℃, and a final extension step of 72℃ for 10 min. PCR products were estimated by 1.0% agarose gel electrophoresis and sent to Tsingke Biotechnology Company (Chengdu, China) for sequencing. All fragments were bidirectional sequences, and

the unsuccessful sequenced fragments were redesigned with primers and sequenced again to complete the sequence.

Table 1. Primers for the PCR amplifications of *Episymploce splendens* (Es) mito-genomes and Numts.

Primer name	Upstream primers sequences(5'-3')	Downstream primers sequences(5'-3')	Anneal temperature (℃)	Extension time (Second)
Es1	140-CCTCTCTTATCGCAATGTCCA	1609-CGTGGGAAAGCTATATCAGGA	58	90
Es2	1367-GGTCAACAAATCATAAAGATATTGG	2074-TAAACTTCAGGGTGACCAAAAAATCA	55	45
Es3	1559-CAACATTTATTTTGATTCTTTGG	3649-GTTTAAGAGACCACCACTTG	50	90
Es4	3401-GAAGACTTTCACCAACCATC	4391-TAGTACACTCATCTACTCTGGTAAC	52	60
Es5	4031-TGTAACAGCCCATGCTT	5797-AATCGCAATGATGGTAGG	51	110
Es6	4945-AGAAGACTTTCACCAACCAT	6997-GGATTCTCAAGATATTCGTT	51	120
Es7	6378-TCAACCGTTATCGAAAGACT	7328-CTCCTACTCCTGTATCTGCTT	52	60
Es8	7010-AACGAATATCCTGAGAATCC	8415-CACGGATTATGTTCTTCAGG	52	90
Es9	8290-GAAGGGGGTGCTGCTATATTAC	11151-ATTACTCCTCCTAATTTATTAGGAAT	62	180
Es10	10491-CAATGAGTATGAGGAGGATTTGCTGT	14755-TGTGCCAGCAGTCGCGGTTATACA	59.5	240
Es11	13822-CAGATTATATTGATTCGCACAAC	303-ATAGAACTGATGAAGCTAAGGC	55	75
Numt-1-1	GATTACGCTGTTATCCCTAAG	GGTGTAACTAGAATGATACAGGT	51	65
Numt-1-2	CGGTTTGAACTCAGATCATGTAAG	GAAGGTGTAACTAGAATGATACAGGT	57	78
Numt-2-1	TGCTACCTTTGCACGGTC	AGGTGAGATAAGTCGTAACATAGT	53	54
Numt-2-2	TAAACTCTATAGGGTCTTCTCG	CTAGAATGATACAGGTTAGGCT	53	70

These sequences, which originated from the invasion of nuclear DNA by mtDNA, were named Numts [15]. Primers that amplified mitochondrial genes may occasionally amplify Numts [16]. In this study, agarose gel electrophoresis of the amplification product (the primer Numt-1-1 and Numt-2-1, Table 1) appeared as two bands. We then redesigned primers (Numt-1-2 and Numt-2-2, Table 1) to amplify these regions and obtained the same result. These non-targeted bands were purified and sequenced, and obtained sequences belonging to mitochondria via the analysis of NCBI blast. Due to differences in mutation rates or freedom from selection pressure, they had some degree of mutation compared to corresponding mito-genome sequences.

Sequence analysis and annotation

DNA SeqMan program, which is included in the Lasergene software package (DNAStar Inc. Madison Wis), was used to assemble sequences to obtain the complete mitochondrial genome. PCGs and rRNAs were identified by comparing *E. splendens* to *B. germanica* and *B. bisignata* [10] [14]. Most of the tRNA and their secondary structure inferences were conducted using the online server ARWEN (http://mbio-serv2.mbioekol.lu.se/ARWEN/) [17]. The tRNA-Ile, tRNA-Phe and tRNA-Leu were not identified by ARWEN; they were manually recognized by referring to secondary structural models of other blattaria insects. The mitogenomic map was depicted with SeqBuilder

(http://www.dnastar.com). The A+T content of the nucleotide sequence and relative synonymous codon usage (RSCU) were calculated using MEGA 5.2 [18]. The AT skewness was calculated according to the following formula: AT skew = [A-T]/[A+T], and the GC skewness was calculated according to the following formula: GC skew = [G-C]/[G+C] [19].

Divergence dating analysis

We performed divergence date analyses based on the aligned sequences of Blattodea, two mantises and two outgroups (S1 Table). The molecular clock was calibrated using three minimum age constraints based on cockroach fossils, as shown in Table 2. A relaxed molecular-clock model was used for this study with the program BEAST 1.6.1 [20]. Rate variation was modeled among branches using uncorrelated lognormal relaxed clocks [20]. A Yule speciation process was used for the tree prior and posterior distributions of parameters, including the tree, were estimated using MCMC sampling [21]. Two independent runs (each with 4 chains) of 100 million generations were sampled every 5000 generations. The tree topology was then estimated using the combined sample from the last 50 million generations of each run.

Table 2. Fossils used for estimation of divergence time of Numts in the analysis.

species	Age (Ma)	Calibration Group	Soft Maximum Bound (97.5% probability)	Reference
Juramantis initialis	145	cockroaches+mantids	250	[22]
Piniblattella sharingolensis	125	cockroaches	250	[23]
Cratomastotermes wolfschwenningeri	113	termites	200	[24]

Phylogenetic inference

Maximum likelihood (ML) and Bayesian inference (BI) analyses were based on the amino acid and nucleotide sequences of 13 proteins in the mitochondrial genome of Orthopteroidea. The sequence of *E. splendens* determined in this study and 67 species downloaded from GenBank were used to perform phylogenetic analyses. Analyses used sequences from 18 cockroaches, 21 termites, 9 mantids, 8 grasshoppers, 9 stick insects and a mantophasmatid (S2 Table). *Papilio protenor* and *Biston panterinaria* were used as outgroups. The nucleotides of 13 PCGs were extracted according to GenBank annotations using GenScalpel, except for the stop codon [25]. The nucleotide and amino acid sequences of each gene were aligned in MEGA 5.2 using default settings, the unaligned and unmatched regions were removed, and the remaining parts were used to reconstruct the phylogenetic relationships.

We used ML and BI to perform phylogenetic analyses, with the best-fit evolution models calculated by PartitionFinder 2.1.1. The best substitution models for nucleotides and amino acid sequences of 13 PCGs

were selected under the Bayesian Information Criterion (BIC) criterion, unlinked branch lengths, and greedy search algorithm parameters [26]. The GTR+I+G model was chosen as the best-fitting model for BI analysis. The BI analysis of nucleotides were then performed with MrBayes 3.2.2 [27]. In the BI analysis, four Markov chains (one cold chain and three hot chains) ran in parallel for 10,000,000 generations, sampling trees every 100 generations and burn-in of 25% trees. For the ML analysis, we implemented the GTR matrix in the PHYML online web server (http://www.atgc-montpellier.fr/phyml/) with 1,000 bootstrap replications [28]. The phylogenetic trees were modified by FigTree v1.4.0 [29].

Results

Genome content and organization

The mitochondrial genome of *E. splendens* was 15,802 bp in length with typical circular molecules. It coded for 35 mitochondrial genes: 13 PCGs, 2 rRNAs (srRNA and lrRNA), 20 tRNAs and the A+T rich region (Fig 1, Table 3). Putative secondary structures of the 20 tRNA genes were shown in S1 Fig. Similar to most species' mitochondria, the coding genes of the *E. splendens* mito-genome are compact, with several genes overlapping. There were five overlaps totaling 21 bp with the two longest overlaps being 7 bp between ATP8 and ATP6, and 7 bp between ND4 and

ND4L. There were 15 gene spacers totaling 190 bp within the entire mitochondrial genome. The longest spacer was 93 bp between tRNA-Val and srRNA, followed by 24 bp between tRNA-Ile and ND2 and 22 bp between NAD1 and tRNA-Leu. The long intergenic spacers have been reported in some Hymenopteran [30] [31], Hemipteran [32] [33], Dictyopteran [34] and Coleopteran insects [5] [35] [36]. This longest spacer (93bp) in *E. splendens* had a similarity of 64.6% compared to its adjacent and corresponding srRNA (S2 Fig). There were 16 areas with neither gene overlap nor intergenic spacer. Additionally, there were 20 tRNAs in the mitochondrial genome of *E. splendens*, lacking two tRNAs located between tRNA-Ile and NAD2, tRNA-Gln and tRNA-Met (Fig 1).

Fig 1. Circular gene map of the mito-genomes of *Episymploce splendens*. Genes coded in the J-strand are inside of the circle. Gene coded in the N-strand are outside of the circle. COX1, COX2 and COX3 refer to the cytochrome C oxidase subunits; CytB refers to cytochrome B; ATPase6 and ATPase8 refer to ATP synthase subunits 6 and 8 genes; and ND1-ND6 and ND4L refer to the NADH dehydrogenase subunit 1-6 and 4Lgenes.

Table 3. Annotation of *Episymploce splendens* (Es).

Gene	Grand	Location	Anticodon	Start codon	Stop codon
tRNA-Ile	J	1..77	TAT		

NAD2	J	102..1148		ATG	TAA
tRNA-Trp	J	1149..1212	TCA		
tRNA-Cys	N	1208..1271	GCA		
tRNA-Tyr	N	1278..1347	GTA		
COI	J	1350..2885		TTG	TAA
tRNA-Leu(UUR)	J	2887..2954	TAA		
COII	J	2956..3642		ATG	TAA
tRNA-Lys	J	3645..3715	CTT		
tRNA-Asp	J	3717..3781	GTC		
ATPase8	J	3782..3940		ATT	TAA
ATPase6	J	3934..4613		ATG	TA-
COIII	J	4614..5402		ATG	TAA
tRNA-Gly	J	5405..5468	TCC		
NAD3	J	5469..5821		ATG	TA-
tRNA-Ala	J	5822..5886	TGC		
tRNA-Arg	J	5885..5950	TCG		
tRNA-Asn	J	5951..6020	GTT		
tRNA-Ser(AGN)	J	6021..6085	GCT		
tRNA-Glu	J	6088..6152	TTC		
tRNA-Phe	N	6162..6228	GAA		
NAD5	N	6229..7947		ATT	TAA
tRNA-His	N	7963..8027	GTG		
NAD4	N	8028..9367		ATG	TA-
NAD4L	N	9361..9642		ATG	TAA
tRNA-Thr	N	9643..9709	TGT		
tRNA-Pro	N	9709..9774	TGG		
NAD6	J	9776..10275		ATT	TA-
CytB	J	10276..11409		ATG	TAA
tRNA-Ser(UCN)	J	11409..11477	TGA		
NAD1	N	11500..12441		ATA	TAG
tRNA-Leu(CUN)	N	12451..12511	TAG		
lrRNA	N	12512..13822			
tRNA-Val	N	13823..13893	TAC		
srRNA	N	13987..14792			
A+T-rich region		14793..15082			

'TA-' refer to incomplete stop codons.

Nucleotide composition and codon usage

We calculated the nucleotide composition of the mtDNA in *E. splendens* using MEGA5.2, refer to S3 Table for detailed results. The

content of A+T (74.6%) was higher than G+C (25. 4%). It corresponded well to the AT bias generally observed in insect mito-genomes, which ranges from 69.5 to 84.9% [32] [37].

The high A+T content and nucleotide skewness of the mitochondrial genome were also reflected in the codon use of protein-coding genes. According to the relative synonymous codon usage (RSCU) value (S4 Table, Fig 2), the occurrence of synonymous codons ending in A or T was much higher than other synonymous codons, and they accounted for 89.7% (3,330) of the total codons. The third position was A or T for all codons with RSCU values greater than 1. The six most frequently used codons were also composed of A and T, namely TTT, TTA, ATT, ATA, TAT and AAT, accounting for 40.7% of the total number of codons. However, the reason for the high content of AT in the mitochondrial genome is still unclear [38].

Fig 2. Relative synonymous codon usage (RSCU) in the mitochondrial genome of *Episymploce splendens*. A total of 3,711 codons for meloids were analyzed, excluding stop codons. Leu, Leu*, Ser, and Ser* indicate trnL1 (CUN), trnL2 (UUR), trnS1 (AGN), and trnS2 (UCN), respectively.

Protein-coding genes

All PCGs of *E. splendens* used ATN as the start codon, except for COI. The COI gene in the *E. splendens* mito-genome used TTG as the starting codon, in agreement with other known cockroaches [39]. The initiation codons of COI genes were various in different insect species, with typical COI triple start codons including ATG and ATC. However, non-ATN trinucleotide (CGA, TTG) codons, tetranucleotides (ATAA, TTAA, and ATTA) and hexanucleotides (ATTTAA) have been found as the start codons of COI genes in some species [40] [41] [42] [43] [44] [45]. The stop codon was most commonly TAA in the *E. splendens* mito-genome, followed by TAG (NAD1). Four protein coding genes: NAD4, NAD6, ATP6, and NAD3, used incomplete TA as the stop codon. The TA stop codon is common in insect mitochondrial genomes and it has been suggested that these incomplete stop codons can be completed by adding A during transcription without affecting translation [2] [46].

A+T rich region

The A+T rich region plays an important role in mitochondrial transcription and replication [47] [48]. Since the control region does not code for genes, the degree of variation in the control region is high and the length varies greatly due to its non-coding characteristic [49]. For example, the non-coding region in insects varies from 70 bp in *Ruspolia dubia* to 9-13 kb in *Pissodes* spp. [50] [51]. The non-coding region of *E. splendens* mito-genome was located between srRNA and tRNA-Ile, and it

was 290 bp long with 64.8% A+T content. Two 120 bp long repeated units separated by a 7 bp interval and four Poly-A structures were found in the A+T rich region of the *E. splendens* mito-genome (Fig 3). Repeated units in the control regions were also found in different blattarian insects. However, these repeated sequences had low similarity in various cockroaches and thus may have different origins [4].

Fig 3. The A +T rich region sequence of *Episymploce splendens*. The blue and gray areas represented two 120bp-long repeated segments, respectively. The red fonts represented the poly-A structures.

Phylogenetic analyses

The phylogenetic relationships were analyzed based on the amino acid and nucleotide sequences of 13 PCGs using ML and BI methods. The ML and BI analyses yielded similar topologies except for the position of Caelifera and *Eupolyphaga sinensis* (Corydiidae) (Fig 4 and S3 Fig). *E. splendens* was located in the basal position of Ectobiidae in four trees. In the ML analysis of 13 PCG's nucleotide sequences, Orthoptera was paraphyletic, with Caelifera being sister to Phasmatodea with a low confidence value (Fig 4). Phasmatodea clustered with Mantophasmatodea with high support in BI trees (S3 Fig). All topologies supported Mantodea as the basal clade of Dictyoptera and (Ectobiidae +

Blaberidae) clustered with the remaining cockroaches (Fig 4 and S3 Fig). For the nodal support, we found bootstrap values in ML analyses were usually lower than the posterior probabilities in BI analyses in several branch nodes, in accordance with Ma et al [4]. Different methods or databases may have a slight influence on topology at the interfamily level, but clearly resulted in a difference to nodal support.

Fig 4. Maximum likelihood (ML) phylogenetic tree based on the nucleotide sequence of 13 PCGs. Numbersabutting branches refer to bootstrap proportions (BSP). *Papilio protenor* and *Biston panterinaria* were employed to root the tree as outgroups.

Numts

There were two Numts found in *E. splendens*, namely Numt-1 (557 bp) and Numt-2 (975 bp). Numt-1 corresponded to partial lrRNA, and Numt-2 was similar to partial lrRNA and its neighboring tRNA-Val of the *E. splendens* mito-genome. Comparisons with mitochondrial sequence showed 87.23% homologies in Numt-1 and 76.63% in Numt-2 (S4 Fig and S5 Fig). We found that the deletion mutation, base substitution and insertion mutation conformed to Numt-1 and Numt-2 [52] [53].

The timescale for evolution of 25 species and Numt-1 diversification based on aligning sequences and calibrations based on three cockroach

fossils is shown in Fig 5(a) while the timescale for Numt-2 is shown in Fig 5(b). The divergence of the lineages leading to *Blattella* and *Episymploce* was 38.52 Ma to 96.76 Ma (95% confidence interval [CI]) in Fig 5(a) while the estimated age of the split between them was 24.52 Ma to 82.03 Ma (95% confidence interval [CI]) in Fig 5(b). Results were similar although different aligning sequences were used to calculate the divergence time in this study (Fig 5). Numt-1 transferred from the mitochondrion to the nucleus between 13.45 Ma to 57.4 Ma (95% confidence interval [CI]), and Numt-2 were estimated to have diverged between 4.36 Ma to 31.66 Ma (95% confidence interval [CI]). Numts have been found in the nuclear genomes of various eukaryotes and can be used as outgroups of phylogeny to solve phylogenetic problems [54] [55] [56]. The two Numts found in this study could be useful tools for the study of cockroach phylogenetic relationships in the future.

Fig 5(a). Phylogenetic chronogram of Numt-1 and blattodean species based on the sequence aligned with NUMT-1, reconstructed using BEAST. An optimal partitioning scheme was determined by PartitionFinder. Scale bar estimates age in millions of years and blue bars represent 95% highest posterior density intervals for the node ages. *Papilio protenor* and *Biston panterinaria* were employed to root the tree as outgroups.

Fig 5(b). Phylogenetic chronogram of Numt-2 and blattodean species based on the sequence aligned with NUMT-2, reconstructed using BEAST. An optimal partitioning scheme was determined by PartitionFinder. Scale bar estimates age in millions of years and blue bars represent 95% highest posterior density intervals for the node ages. *Papilio protenor* and *Biston panterinaria* were employed to root the tree as outgroups.

Discussion

Intergenic spacer

We found that the *E. splendens* mito-genome typically exhibited compact arrangements, such as small gene spacing, gene overlap, or incomplete stop codons [57]. However, the *E. splendens* mito-genome had a long intergenic spacer region (93 bp) located between tRNA-Val and srRNA, which is the longest intergenic spacer region of the reported cockroach mito-genomes. There are two commonly posited evolutionary mechanisms for the origin of mitochondrial intergenic spacers, the duplication/random loss model and slipped-strand mispairing [5] [34]. We could not find a homologous sequence with both ends in this intergenic spacer, thus its formation is difficult to explain by slipped-strand mispairing [5] [58]. A similar long intergenic spacer

reported in a blattarian insect mito-genome, *Blaptica dubia* (71-bp between tRNA-Gln and tRNA-Met) [34], the duplication/random loss model could be used to explain the formation of this intergenic spacer [5] [34][59] [60]. We suggested that this intergenic region may be derived from the replication of the 3’ end of the srRNA when the DNA double helix unraveled, followed by random loss of partial duplicated gene, and then the residues formed the 93-bp remaining intergenic spacer in *E. splendens* (Fig 6).

Fig 6. Putative mechanism of the intergenic spacer in mitochondrial genome of *E. splendens*. The Randomly copied fragment was marked with *

Animal mito-genomes were generally considered to be economic and optimized for rapid replication and transcription [61]. Therefore, mitochondrial evolution had traditionally been regarded as favoring genome size reduction [3] [62] [63], possibly by eliminating intergenic spacers [64]. Eliminating nonfunctional intergenic spacers in mitochondrial evolution was important in the highly reduced and efficient mito-genomes [36]. But with the discovery of more large intergenic spacers in mito-genomes, as several containing additional origin of replication (*Apis mellifera*, *Triatoma dimidiata*, *Bombus ignitus*) [32] [37]

[65], tandem repeat units (*Triatoma dimidiata*, *Pyrocoelia rufa*) [32] [35], or even open reading frames (*Triatoma dimidiata*) [32], whether these long spacer regions were functional was controversial [32] [66]. We analyzed the large intergenic spacers in the mito-genomes of several insects, comparing the length and similarity of these large intergenic spacer sequences with their root sequences (S5 Table) [5] [33] [34]. We found these spacer sequences were usually shorter than their root sequences and the greater the length difference between these spacer and their root sequences, the lower the similarity between them. It implied that these mito-genomes lost partial duplicated nucleotides in mitochondrial evolution. In addition, large intergenic spacers found in several genetically close species were not homologous, implying their origin occurred independently after species differentiation [5]. This suggests that these long intergenic spacers did not confer an evolutionary advantage, and they were gradually deleted during mitochondrial evolution. Consequently, we consider that the mito-genome may continue evolving towards compact arrangement and the long intergenic region may gradually decrease or even disappear during mito-genome evolution. However, these discoveries of large intergenic spacers in insect mito-genomes contribute to species identification, and also provide valuable information for the study of the evolution of insect mitochondrial genomes.

tRNAs deletion

Mitochondrial gene content, arrangement and composition were highly conserved, and mito-genomes typically contained 37 coding genes [3] [67]. However, some exceptions, such as gene duplications, deletions or arrangements, were found in several species [68] [69] [70]. tRNA deletion events were unusual and only several cases have been reported in prokaryotes, unicellular organisms [71] [72], *Placobdella* [73], amphibians (*Limnonectes bannaensis* and *Gegeneophis ramaswamii*) [68] [74], reptile (*Platysternon megacephalum*) [75], marsupial [76], and insects (*Urochela quadrinotata* Reuter, *Dendroctonus armandi*, *Scolytoplatypus mikado*) [77] [78]. In this study, two tRNAs, tRNA-Gln and tRNA-Met, were absent in the mito-genome of *E. splendens*. The tRNA deletions was only found in the *E. splendens* mito-genome (this stuy) in all Orthopteroidea mito-genomess reported and the tRNA deletion events reported in previous research also scattered in different clade branches, therefore, we consider tRNA deletions appear to be separate events occasionally occurring in some species or evolutionary branches. The deletion of functional genes was obviously disadvantageous for species, and the mechanism of deletion is still unclear.

The organism may have a functional replacement to cope with the

loss of tRNAs. Two mechanisms were proposed for this functional compensation. The first mechanism where tRNAs from the cytosol are imported into mitochondria has been confirmed, as aminoacyl-tRNA synthetases being imported from the cytosol into mitochondria [79], and functional tRNA-Lys encoded in the nuclear genome being imported into marsupial mitochondria [76]. The second compensation mechanism for the missing tRNAs is 'superwobble', where a tRNA with an unmodified U in the wobble position reads all four nucleotides in the third codon position [80]. In our study, the loss of tRNA-Gln and tRNA-Met can be compensated with their first and second codons matching His and Ile, respectively, and the anticodon swing site U for His and Ile. We calculated the Relative Synonymous Codon Usage (RSCU) values of CAU (H) and AUU (I) in reported cockroach mito-genomes, and discovered that *E. splendens* mito-genome had the highest RSCU values (Table 4), indicating that *E. splendens* might be compensated through tRNA superwobble. Regardless, we cannot exclude tRNA import from the cytosol in *E. splendens* mitochondrion. Therefore, more studies are needed to confirm the compensation mechanism for the absence of tRNAs in the mito-genome of *E. splendens*.

Table 4. The RSCU values of CAU (H) and AUU (I) in known cockroaches

Cockroaches	CAU (H)	AUU (I)	GenBank Accession number
Episymploce splendens	1.69	1.86	

Blattella germanica	1.54	1.83	NC_012901.1
Blattella bisignata	1.57	1.63	NC_018549.1
Panchlora nivea	1.57	1.77	NC_030002.1
Shelfordella lateralis	1.63	1.63	NC_030003.1
Nauphoeta cinerea	1.49	1.64	NC_035052.1
Cryptocercus meridianus	1.51	1.49	NC_037496.1
Periplaneta australasiae	1.44	1.7	NC_034841.1
Neostylopyga rhombifolia	1.45	1.66	NC_034842.1
Periplaneta brunnea	1.42	1.71	MG010455
Blaptica dubia	1.55	1.54	NC_029224.1
Periplaneta americana	1.36	1.71	NC_016956.1
Gromphadorhina portentosa	1.45	1.42	NC_030001.1
Periplaneta fuliginosa	1.43	1.73	NC_006076.1
Opisthoplatia orientalis	1.35	1.6	NC_029225.1
Cryptocercus relictus	1.21	1.57	NC_018132.1
Cryptocercus kyebangensis	1.33	1.6	NC_030191.1
Eupolyphaga sinensis	0.93	1.64	NC_014274.1

Phylogenetic relationships of Dictyoptera

The Blattodea, Isoptera and Mantodea all belonged to Dictyoptera based on morphological and anatomical characteristics [11] [12]. According to molecular and morphology data, Mantodea was considered as the basal split within Dictyoptera [81] [82]. However, DeSalle *et al* [83] supported that Mantodea and Isoptera were sister groups within Dictyoptera. Nevertheless, more recent studies discovered that Mantodea was the sister clade to (Isoptera + Blattodea), and even indicated that Isoptera should be classified as a family (Termitidae) within Blattodea [34] [39] [84]. In our study, all results supported Mantodea was a sister clade to Blattodea (including Blattodea and Isoptera) with high support. In addition, we found Isoptera clustered into a monophyletic clade with high support (Fig 4 and S3 Fig), and this was also supported by several

previous studies [9] [12] [85]. Consequently, we propose that Isoptera should be classified as a family within Blattodea.

Although about 4,000 species of cockroaches have been recorded, the phylogenetic relationships within the Blattodea have been debated [86]. Blaberoidea was comprised of Blaberidae and Ectobiidae. The relationships within Blattodea, between Blaberoidea and the remaining cockroaches, have been disputed. Most researchers have supported Blaberoidea being clustered with the remaining cockroaches, while some studies supported Blaberoidea as the sister group to Blattidae [4] [8] [10] [87]. In our study, all trees strongly supported Blaberoidea being placed in the basal position of Blattodea. In addition, within Blaberoidea, we found Ectobiidae was paraphyletic while Blaberoidea was monophyletic (Fig 4 and S3 Fig), consistent with several previous studies [7] [88]. However, more Ectobiidae samples needed to be added in the future to confirm our finding.

The position of Corydiidae within Blattodea has been debated over the last 20 years. According to morphological and anatomical data, some researchers supported Corydiidae was the sister group to Ectobiidae [81] [89]. Meanwhile, a few studies suggested that Corydiidae was the sister clade to Blaberoidea based on mitochondrial and nuclear genes [90]. Some researchers suggested that Corydiidae be recovered as the sister group to Nocticolidae and placed (Corydiidae + Nocticolidae) as sister to

the remaining Blattodea, according to different analysis methods [7] [85] [91]. Various analysis methods and small sample size may have effect on the reconstructed results [4]. Maekawa and Matsumoto placed Corydiidae as the basal position of Blattodea using the COII gene [8]. Xiao *et al* suggested Corydiidae was a sister clade to Isoptera based on whole mtDNA sequences, concatenated nucleotide sequences and concatenated amino acid sequences, and this relationship was supported by two other studies [10] [84] [92]. According to 12S, 16S, 18S and COII genes, Pellens *et al* [93] supported Corydiidae and Cryptocercidae + Isoptera + Blattidae as sister groups. Gong *et al* [39] supported the relationship (Corydiidae + (Cryptocercidae + Isoptera)) based on the nucleotide dataset of 13 PCGs. In our study, three trees supported the relationship (Blattidae + (Corydiidae + (Cryptocercidae + Isoptera))) with high support (Fig 4, S3(a) Fig and S3(c) Fig). However, we found the BI tree of nucleotide sequences of 13 PCGs supported the relationship (Corydiidae + (Blattidae + (Cryptocercidae + Isoptera))) (S3(b) Fig).

Conclusion

In this study, we sequenced and annotated the *E. splendens* mito-genome. We found that two tRNAs (tRNA-Gln, tRNA-Met) were absent in *E. splendens* mito-genome, which is the first report of tRNAs deletion in reported Orthopteroidea mito-genomes. A long intergenic region between tRNA-Val and srRNA (93 bp, with a 64.6% similarity

with its corresponding srRNA) was also found. The duplication/random loss model may account for the origin of this long intergenic spacer. We also found two Numts, Numt-1 and Numt-2 transfered from mitochondrion to nucleus at about 13.45 Ma to 57.4 Ma, and 4.36 Ma to 31.66 Ma respectively. Phylogenetic inference based on 13 PCGs supported Isoptera should be classified as a family within Blattodea and Mantodea was the sister clade to (Isoptera + Blattodea). Also, our results supported (Blattidae + (Corydiidae + (Cryptocercidae + Isoptera))) and Blaberoidea being placed in the basal position of Blattodea.

Acknowledgments

We sincerely appreciate Natural History Museum of Sichuan University for the sample collection. Thanks Dr. Megan for language help. This study was funded by the Science and Technology Project of Sichuan Province (2017SZ0019), Special funds for central government to guide local scientific and Technological Development (2020ZYD098) and State Administration of Traditional Chinese Medicine of the P.R.C (ZYBZH-C-SC-53).

Author Contributions

Conceptualization: XYZ JNM BSY.

Funding acquisition: XYZ.

Investigation: LY ZZH HMW JG QC CJZ.

Resources: LY BSY XYZ JG CJZ.

Writing – original draft: LY ZZH HMW JG QC.

Writing – review & editing: LY XYZ JNM BSY.

References

[1] Wilson AJ, Xu J. Mitochondrial inheritance: diverse patterns and mechanisms with an emphasis on fungi. Mycology. 2012; 3(2): 158-166. doi: 10.1080/21501203.2012.684361. PMID: 7219536.

[2] Ojala D, Montoya J, Attardi G. tRNA punctuation model of RNA processing in human mitochondria. Nature. 1981; 290(5806): 470-474. doi: 10.1038/290470a0. PMID: 7219536.

[3] Boore JL. Animal mitochondrial genomes. Nucleic Acids Res. 1999; 27(8): 1767-1780. doi: 10.1093/nar/27.8.1767. PMID: 10101183.

[4] Ma JN, Du C, Zhou C, Shen YM, Fan ZX, Yue BS, et al. (2017) Complete mitochondrial genomes of two blattid cockroaches, *Periplaneta australasiae* and *Neostylopyga rhombifolia*, and phylogenetic relationships within the Blattaria. PLoS One. 2017; 12(5): e0177162. doi: 10.1371/journal.pone.0177162. PMID: 28486518.

[5] Du C, Zhang L, Lu T, Ma J, Chen Z, Yue B, et al. Mitochondrial genomes of blister beetles (Coleoptera, Meloidae) and two large

intergenic spacers in *Hycleus* genera. BMC Genomics. 2017; 18(1): 698. doi: 10.1186/s12864-017-4102-y. PMID: 28874137.

[6] Menasria T, Moussa F, El-Hamza S, Tina S, Megri R, Chenchouni H. Bacterial load of German cockroach (*Blattella germanica*) found in hospital environment. Pathog Glob Health. 2014; 108(3):141-147. doi: 10.1179/2047773214Y.0000000136. PMID: 24766338; PMCID: PMC4083176.

[7] Legendre F, Nel A, Svenson GJ, Robillard T, Pellens R, Grandcolas P. Phylogeny of Dictyoptera: Dating the Origin of Cockroaches, Praying Mantises and Termites with Molecular Data and Controlled Fossil Evidence. PLoS One. 2015; 10(7): e0130127. doi: 10.1371/journal.pone.0130127. PMID: 26200914; PMCID: PMC4511787.

[8] Maekawa K, Matsumoto T. Molecular phylogeny of cockroaches (Blattaria) based on mitochondrial COII gene sequences. Syst Entomol. 2000; 25(4): 511-519. doi: 10.1046/j.1365-3113.2000.00128.x

[9] Inward D, Beccaloni G, Eggleton P. Death of an order: a comprehensive molecular phylogenetic study confirms that termites are eusocial cockroaches. Biol Lett. 2007; 3(3): 331-335. PMID: 17412673; PMCID: PMC2464702.

[10] Xiao B, Chen AH, Zhang YY, Jiang GF, Hu CC, Zhu CD. Complete mitochondrial genomes of two cockroaches, *Blattella germanica* and

Periplaneta americana, and the phylogenetic position of termites. Curr Genet. 2012; 58(2): 65-77. doi: 10.1007/s00294-012-0365-7. PMID: 22311390.

[11] Djernæs M, Klass KD, Picker MD, Damgaard J. Phylogeny of cockroaches (Insecta, Dictyoptera, Blattodea), with placement of aberrant taxa and exploration of out-group sampling. Syst Entomol. 2012; 37(1): 65-83. doi: 10.1111/j.1365-3113.2011.00598.x

[12] Djernæs M, Klass KD, Eggleton P. Identifying possible sister groups of Cryptocercidae+Isoptera: a combined molecular and morphological phylogeny of Dictyoptera. Mol Phylogenet Evol. 2015; 84: 284-303. doi: 10.1016/j.ympev.2014.08.019. PMID: 25194324.

[13] Wang Z, Che Y. Three new species of cockroach genus *Symploce* Hebard, 1916 (Blattodea, Ectobiidae, Blattellinae) with redescriptions of two known species based on types from Mainland China. Zookeys. 2013; 337(337): 1-18. doi: 10.3897/zookeys.337.5770. PMID: 24146575.

[14] Chen AH. Complete mitochondrial genome of the double-striped cockroach *Blattella bisignata* (Insecta: Blattaria: Blaberoidea). Mitochondrial DNA. 2013; 24(1): 14-6. doi: 10.3109/19401736.2012.710228. PMID: 22897805.

[15] Lopez JV, Yuhki N, Masuda R, Modi W, O'Brien SJ. Numt, a recent transfer and tandem amplification of mitochondrial DNA to the nuclear

genome of the domestic cat. J Mol Evol. 1994; 39(2): 174-90. doi: 10.1007/BF00163806. PMID: 7932781.

[16] Arctander P. Comparison of a mitochondrial gene and a corresponding nuclear pseudogene. Proc Biol Sci. 1995; 262(1363): 13-9. doi: 10.1098/rspb.1995.0170. PMID: 7479989.

[17] Laslett D, Canbäck B. ARWEN: a program to detect tRNA genes in metazoan mitochondrial nucleotide sequences. Bioinformatics. 2008; 24(2): 172-5. doi: 10.1093/bioinformatics/btm573. PMID: 18033792.

[18] Tamura K, Peterson D, Peterson N, Stecher G, Nei M, Kumar S. MEGA5: molecular evolutionary genetics analysis using maximum likelihood, evolutionary distance, and maximum parsimony methods. Mol Biol Evol. 2011; 28(10): 2731-2739. doi: 10.1093/molbev/msr121. PMID: 21546353; PMCID: PMC3203626.

[19] Perna NT, Kocher TD. Patterns of nucleotide composition at fourfold degenerate sites of animal mitochondrial genomes. J Mol Evol. 1995; 41(3): 353-358. PMID: 7563121.

[20] Drummond AJ, Rambaut A. BEAST: Bayesian evolutionary analysis by sampling trees. BMC Evol Biol. 2007; 7: 214. doi: 10.1186/1471-2148-7-214

[21] Gernhard T. The conditioned reconstructed process. J Theor Biol. 2008; 253(4): 769-778. doi: 10.1016/j.jtbi.2008.04.005. PMID: 18538793.

[22] Vrsˇansky P. (2002) Origin and the early evolution of mantises. AMBA Projekty. 2002; 6(1): 1-16. Retrieved from http://scholar.google.com/scholar?hl=en&btnG=Search&q=intitle:Origin+and+early+evolution+of+mantises#0

[23] Vrsˇansky´ P. Lower Cretaceous cockroaches and mantids (Insecta: Blattaria, Mantodea) from the Sharin-Gol in Mongolia. Entomol Probl. 2005; 35(2): 163-167. Retrieved from http://fossilinsects.net/pdfs/Vrso_2005_SharinGol.pdf

[24] Makarkin VN, Menon F. First record of fossil 'rapismatid-like' Ithonidae (Insecta, Neuroptera) from the Lower Cretaceous Crato Formation of Brazil. Cretaceous Res. 2007; 28(5): 743-753. doi: 10.1016/j.cretres.2006.11.003

[25] Yin H, Zhi Y, Jiang H, Wang P, Yin X, Zhang D. The complete mitochondrial genome of *Gomphocerus tibetanus* Uvarov, 1935 (Orthoptera: Acrididae: Gomphocerinae). Gene. 2012; 494(2): 214-218. doi: 10.1016/j.gene.2011.12.020. PMID: 22209720.

[26] Lanfear R, Frandsen PB, Wright AM, Senfeld T, Calcott B. PartitionFinder 2: New Methods for Selecting Partitioned Models of Evolution for Molecular and Morphological Phylogenetic Analyses. Mol Biol Evol. 2017; 34(3):772-773. doi: 10.1093/molbev/msw260. PMID: 28013191.

[27] Ronquist F, Teslenko M, van der Mark P, Ayres DL, Darling A, Höhna S, et al. MrBayes 3.2: efficient Bayesian phylogenetic inference and model choice across a large model space. Syst Biol. 2012; 61(3): 539-42. doi: 10.1093/sysbio/sys029. PMID: 22357727; PMCID: PMC3329765.

[28] Guindon S, Lethiec F, Duroux P, Gascuel O. PHYML Online--a web server for fast maximum likelihood-based phylogenetic inference. Nucleic Acids Res. 2005; 33(Web Server issue): W557-559. PMID: 15980534; PMCID: PMC1160113.

[29] Rambaut A. FigTree, a graphical viewer of phylogenetic trees. 2007. Available: http://treebioed.ac.uk/software/figtree

[30] Wei SJ, Tang P, Zheng LH, Shi M, Chen XX. The complete mitochondrial genome of *Evania appendigaster* (hymenoptera: Evaniidae) has low A+T content and a long intergenic spacer between atp8 and atp6. Mol Biol Rep. 2010; 37(4): 1931-42. doi:10.1007/s11033-009-9640-1. PMID:19655273.

[31] Rodovalho CM, Lyra ML, Ferro M, Jr MB. The mitochondrial genome of the leaf-cutter ant *Atta laevigata*: a mitogenome with a large number of intergenic spacers. PLoS One. 2014; 9(5): e97117. doi: 10.1371/journal.pone.0097117. PMID: 24828084.

[32] Dotson EM, Beard CB. Sequence and organization of the mitochondrial genome of the Chagas disease vector, *Triatoma dimidiata*. Insect Mol Biol. 2001; 10(3): 205-215. PMID: 11437912.

[33] Hua J, Li M, Dong P, Cui Y, Xie Q, Bu W. Comparative and phylogenomic studies on the mitochondrial genomes of Pentatomomorpha (Insecta: Hemiptera: Heteroptera). BMC Genomics. 2008; 9(1): 1-15. doi: 10.1186/1471-2164-9-610. PMID: 19091056; PMCID: PMC2651891.

[34] Cheng XF, Zhang LP, Yu DN, Storey KB, Zhang JY. The complete mitochondrial genomes of four cockroaches (Insecta: Blattodea) and phylogenetic analyses within cockroaches. Gene. 2016; 586(1): 115-122. doi: 10.1016/j.gene.2016.03.057. PMID: 27045773.

[35] Bae JS, Kim I, Sohn HD, Jin BR. The mitochondrial genome of the firefly, *Pyrocoelia rufa*: complete DNA sequence, genome organization, and phylogenetic analysis with other insects. Mol Phylogenet Evol. 2004; 32(3): 978-85. doi: 10.1016/j.ympev.2004.03.009. PMID: 15288070.

[36] Sheffield NC, Song H, Cameron SL, Whiting MF. A comparative analysis of mitochondrial genomes in Coleoptera (Arthropoda: Insecta) and genome descriptions of six new beetles. Mol Biol Evol. 2008; 25(11): 2499-2509. doi: 10.1093/molbev/msn198. PMID: 18779259.

[37] Crozier RH, Crozier YC. The mitochondrial genome of the honeybee *Apis mellifera*: complete sequence and genome organization. Genetics. 1993; 133(1): 97-117. PMID: 8417993; PMCID: PMC1205303.

[38] Miya M, Nishida M. Organization of the Mitochondrial Genome of a Deep-Sea Fish, *Gonostoma gracile* (Teleostei: Stomiiformes): First Example of Transfer RNA Gene Rearrangements in Bony Fishes. Mar Biotechnol (NY). 1999; 1(5): 416-0426. doi: 10.1007/pl00011798. PMID: 10525676.

[39] Gong R, Guo X, Ma J, Song X, Shen Y, Geng F, et al. Complete mitochondrial genome of *Periplaneta brunnea* (Blattodea: Blattidae) and phylogenetic analyses within Blattodea. J Asia-Pac Entomol. 2018; 21(3): 885-895. doi: 10.1016/j.aspen.2018.05.006

[40] Liu Y, Li Y, Pan M, Dai F, Zhu X, Lu C, et al. The complete mitochondrial genome of the Chinese oak silkmoth, *Antheraea pernyi* (Lepidoptera: Saturniidae). Acta Bioch Bioph Sin. 2008; 40(8): 693-703. doi: 10.1093/abbs/40.8.693

[41] Kim SR, Kim MI, Hong MY, Kim KY, Kang PD, Hwang JS, et al. The complete mitogenome sequence of the Japanese oak silkmoth, *Antheraea yamamai* (Lepidoptera: Saturniidae). Mol Biol Rep. 2009; 36(7): 1871-80. doi: 10.1007/s11033-008-9393-2. PMID: 18979227.

[42] Hong MY, Lee EM, Jo HY, Park HC, Kim SR, Hwang JS, et al. Complete nucleotide sequence and organization of the mitogenome of the

silk moth Caligula boisduvalii (Lepidoptera: Saturniidae) and comparison with other lepidopteran insects. Gene. 2008; 413(1-2): 49-57. doi: 10.1016/j.gene.2008.01.019. PMID: 18337026.

[43] Yang L, Wei ZJ, Hong GY, Jiang ST, Wen LP. The complete nucleotide sequence of the mitochondrial genome of *Phthonandria atrilineata* (Lepidoptera: Geometridae). Mol Biol Rep. 2009; 36(6): 1441-9. doi: 10.1007/s11033-008-9334-0. PMID: 18696255.

[44] Kim I, Lee EM, Seol KY, Yun EY, Lee YB, Hwang JS, et al. The mitochondrial genome of the Korean hairstreak, *Coreana raphaelis* (Lepidoptera: Lycaenidae). Insect Mol Biol. 2006; 15(2): 217-25. doi: 10.1111/j.1365-2583.2006.00630.x. PMID: 16640732.

[45] Coates BS, Sumerford DV, Hellmich RL, Lewis LC. Partial mitochondrial genome sequences of *Ostrinia nubilalis* and *Ostrinia furnicalis*. Int J Biol Sci. 2005; 1(1): 13-18. doi: 10.7150/ijbs.1.13. PMID: 15951845.

[46] Lavrov DV, Boore JL, Brown WM. Complete mtDNA sequences of two millipedes suggest a new model for mitochondrial gene rearrangements: duplication and nonrandom loss. Mol Biol Evol. 2002; 19(2): 163-9. doi: 10.1093/oxfordjournals.molbev.a004068. PMID: 11801744.

[47] Clayton DA. Replication of animal mitochondrial DNA. Cell. 1982; 28(4): 693-705. doi: 10.1016/0092-8674(82)90049-6. PMID: 6178513.

[48] Clayton DA. Replication and Transcription of Vertebrate Mitochondrial DNA. Annu Rev Cell Biol. 1991; 7: 453-78. doi: 10.1146/annurev.cb.07.110191.002321. PMID: 1809353.

[49] Wolstenholme DR. Animal mitochondrial DNA: structure and evolution. Int Rev Cytol. 1992; 141: 173-216. doi: 10.1016/s0074-7696(08)62066-5. PMID: 1452431.

[50] Zhou Z, Huang Y, Shi F. The mitochondrial genome of *Ruspolia dubia* (Orthoptera: Conocephalidae) contains a short A+T-rich region of 70 bp in length. Genome. 2007; 50(9): 855-66. doi: 10.1139/g07-057. PMID: 17893726.

[51] Boyce TM, Zwick ME, Aquadro CF. Mitochondrial DNA in the bark weevils: size, structure and heteroplasmy. Genetics. 1989; 123(4): 825-36. PMID: 2612897; PMCID: PMC1203892.

[52] Tourmen Y, Baris O, Dessen P, Jacques C, Malthièry Y, Reynier P. Structure and chromosomal distribution of human mitochondrial pseudogenes. Genomics. 2002; 80(1): 71-7. doi: 10.1006/geno.2002.6798. PMID: 12079285.

[53] Hazkani-Covo E, Sorek R, Graur D. Evolutionary dynamics of large numts in the human genome: rarity of independent insertions and abundance of post-insertion duplications. J Mol Evol. 2003; 56(2): 169-74. doi: 10.1007/s00239-002-2390-5. PMID: 12574863.

[54] Thalmann O, Serre D, Hofreiter M, Lukas D, Eriksson J, Vigilant L. Nuclear insertions help and hinder inference of the evolutionary history of gorilla mtDNA. Mol Ecol. 2005; 14(1): 179-88. doi: 10.1111/j.1365-294X.2004.02382.x. PMID: 15643961.

[55] Zischler H, Geisert H, von Haeseler A, Pääbo S. A nuclear 'fossil' of the mitochondrial D-loop and the origin of modern humans. Nature. 1995; 378(6556): 489-92. doi: 10.1038/378489a0. PMID: 7477404.

[56] Hay JM, Sarre SD, Daugherty CH. Nuclear mitochondrial pseudogenes as molecular outgroups for phylogenetically isolated taxa: a case study in Sphenodon. Heredity (Edinb). 2004; 93(5): 468-75. doi: 10.1038/sj.hdy.6800525. PMID: 15266297.

[57] Minton RL, Cruz MAM, Farman ML, Perez KE. Two complete mitochondrial genomes from *Praticolella mexicana* Perez, 2011 (*Polygyridae*) and gene order evolution in Helicoidea (*Mollusca*, *Gastropoda*). Zookeys. 2016; (626): 137-154.

[58] Stanton DJ, Daehler LL, Moritz CC, Brown WM. Sequences with the potential stem-and-Ioop structures are associated with coding-region duplications in animal mitochondrial DNA. Genetics. 1994; 137(1): 233-241. doi: 10.1101/gad.8.9.1131. PMID: 8056313.

[59] Moritz C, Dowling TE, Brown WM. Evolution of Animal Mitochondrial DNA: Relevance for Population Biology and Systematics.

Annu Rev Ecol Evol S. 1987; 18: 269-292. doi: 10.1146/annurev.ecolsys.18.1.269

[60] Boore JL. Comparative Genomics: The duplication/random loss model for gene rearrangement exemplified by mitochondrial genomes of Deuterostome animals. 1st ed. Dordrecht: Springer; 2000.

[61] Sayadi A, Immonen E, Tellgren-Roth C, Arnqvist G. The Evolution of Dark Matter in the Mitogenome of Seed Beetles. Genome Biol Evol. 2017; 9(10): 2697-2706. doi: 10.1093/gbe/evx205. PMID: 29048527; PMCID: PMC5737749.

[62] Macey JR, Larson A, Ananjeva NB, Fang Z, Papenfuss TJ. Two novel gene orders and the role of light-strand replication in rearrangement of the vertebrate mitochondrial genome. Mol Biol Evol. 1997; 14(1): 91-104. doi: 10.1093/oxfordjournals.molbev.a025706. PMID: 9000757.

[63] McKnight ML, Shaffer HB. Large, rapidly evolving intergenic spacers in the mitochondrial DNA of the salamander family Ambystomatidae (Amphibia: Caudata). Mol Biol Evol. 1997; 14(11): 1167-76. doi: 10.1093/oxfordjournals.molbev.a025726. PMID: 9364774.

[64] Burger G, Gray MW, Lang BF. Mitochondrial genomes: anything goes. Trends Genet. 2003; 19(12): 709-16. doi: 10.1016/j.tig.2003.10.012. PMID: 14642752.

[65] Cha SY, Yoon HJ, Lee EM, Yoon MH, Hwang JS, Jin BR, et al. The complete nucleotide sequence and gene organization of the mitochondrial genome of the bumblebee, *Bombus ignitus* (Hymenoptera: Apidae). Gene. 2007; 392(1-2): 206-20. doi: 10.1016/j.gene.2006.12.031. PMID: 17321076.

[66] Jørgensen TE, Bakke I, Ursvik A, Andreassen M, Moum T, Johansen SD. An evolutionary preserved intergenic spacer in gadiform mitogenomes generates a long noncoding RNA. BMC Evol Biol. 2014; 14: 182. doi: 10.1186/s12862-014-0182-3. PMID: 25145347; PMCID: PMC4236577.

[67] Gemmell NJ, Janke A, Western PS, Watson JM, Pääbo S, Graves JA. Cloning and characterization of the platypus mitochondrial genome. J Mol Evol. 1994; 39(2): 200-5. doi: 10.1007/BF00163808. PMID: 7932783.

[68] Zhang JF, Nie LW, Wang Y, Hu LL. The complete mitochondrial genome of the large-headed frog, *Limnonectes bannaensis* (Amphibia: Anura), and a novel gene organization in the vertebrate mtDNA. Gene. 2009; 442(1-2): 119-27. doi: 10.1016/j.gene.2009.04.018. Epub 2009 May 3. PMID: 19397958.

[69] Hlaing T, Tun-Lin W, Somboon P, Socheat D, Setha T, Min S, Chang MS, Walton C. Mitochondrial pseudogenes in the nuclear genome of *Aedes aegypti* mosquitoes: implications for past and future population

genetic studies. BMC Genet. 2009; 10: 11. doi: 10.1186/1471-2156-10-11. PMID: 19267896; PMCID: PMC2660364.

[70] von Nickisch-Rosenegk M, Brown WM, Boore JL. Complete sequence of the mitochondrial genome of the tapeworm *Hymenolepis diminuta*: gene arrangements indicate that Platyhelminths are Eutrochozoans. Mol Biol Evol. 2001; 18(5): 721-30. doi: 10.1093/oxfordjournals.molbev.a003854. PMID: 11319256.

[71] Adams KL, Palmer JD. Evolution of mitochondrial gene content: gene loss and transfer to the nucleus. Mol Phylogenet Evol. 2003; 29(3): 380-95. doi: 10.1016/s1055-7903(03)00194-5. PMID: 14615181.

[72] Tran TT, Belahbib H, Bonnefoy V, Talla E. A Comprehensive tRNA Genomic Survey Unravels the Evolutionary History of tRNA Arrays in Prokaryotes. Genome Biol Evol. 2015; 8(1): 282-95. doi: 10.1093/gbe/evv254. PMID: 26710853; PMCID: PMC4758250.

[73] Jiménez-Armenta J, Kvist S, Oceguera-Figueroa A. An exceptional case of mitochondrial tRNA duplication-deletion events in blood-feeding leeches. Org Divers Evol. 2020; 20: 221-231. doi: 10.1007/s13127-020-00431-6

[74] San MD, Gower DJ, Oommen OV, Wilkinson M, Zardoya R. Phylogeny of caecilian amphibians (Gymnophiona) based on complete mitochondrial genomes and nuclear RAG1. Mol Phylogenet. 2004; 33(2): 413-27. doi:10.1016/j.ympev.2004.05.014

[75] Peng QL, Nie LW, Pu YG. Complete mitochondrial genome of Chinese big-headed turtle, *Platysternon megacephalum*, with a novel gene organization in vertebrate mtDNA. Gene. 2006; 380(1): 14-20. doi: 10.1016/j.gene.2006.04.001. PMID: 16842936.

[76] Dorner M, Altmann M, Paabo S, Morl M. Evidence for import of a lysyl-tRNA into marsupial mitochondria. Mol Biol Cell. 2001; 12(9): 2688-2698. doi: 10.1091/mbc.12.9.2688

[77] Dai Y, Li L, Jiang P, Song F, Ye Z, Yuan X, et al. Sequence and organization of the mitochondrial genome of an urostylidid bug, *Urochela quadrinotata* Reuter (Hemiptera: Urostylididae). Entomotaxonomia, 2012; 34, 613-623.

[78] Yang Y. Determination and analysis of mitochondrial genomes of four species of Curculionoidea. Shaanxi Normal University, 2017.

[79] Duchêne AM, Pujol C, Maréchal-Drouard L. Import of tRNAs and aminoacyl-tRNA synthetases into mitochondria. Curr Genet. 2009; 55(1): 1-18. doi: 10.1007/s00294-008-0223-9. PMID: 19083240.

[80] Rogalski M, Karcher D, Bock R. Superwobbling facilitates translation with reduced tRNA sets. Nat Struct Mol Biol. 2008; 15(2): 192-8. doi: 10.1038/nsmb.1370. PMID: 18193063.

[81] Roth LM. Systematics and phylogeny of cockroaches (Dictyoptera: Blattaria). Orient Insects. 2003; 37(1): 1-186. doi: 10.1080/00305316.2003.10417344

[82] Klass KD, Meier R. A phylogenetic analysis of Dictyoptera (Insecta) based on morphological characters. Entomol Abh. 2006; 63: 3-50. doi: 10.2514/6.1984-2395

[83] DeSalle R, Gatesy J, Wheeler W, Grimaldi D. DNA sequences from a fossil termite in Oligo-Miocene amber and their phylogenetic implications. Science. 1992; 257: 1933-1936.

[84] Zhang YY, Xuan WJ, Zhao JL, Zhu CD, Jiang GF. The complete mitochondrial genome of the cockroach *Eupolyphaga sinensis* (Blattaria: Polyphagidae) and the phylogenetic relationships within the Dictyoptera. Mol Biol Rep. 2010; 37(7): 3509-16. doi: 10.1007/s11033-009-9944-1. PMID: 20012368.

[85] Murienne J. Molecular data confirm family status for the *Tryonicus*-*Lauraesilpha* group (Insecta: Blattodea: Tryonicidae). Org Divers Evol. 2009; 9(1): 44-51. doi: 10.1016/j.ode.2008.10.005

[86] Velez A, Wolff M, Gutierrez E. Blattaria of Colombia: list and distribution of genera. Zootaxa. 2006; 1210: 39-52. Available from: https://www.researchgate.net/publication/228622554

[87] Kambhampati S. A phylogeny of cockroaches and related insects based on DNA sequence of mitochondrial ribosomal RNA genes. Proc Natl Acad Sci U.S.A. 1995; 92(6): 2017-2020. doi: 10.1073/pnas.92.6.2017. PMID: 7534409; PMCID: PMC42414.

[88] Roth S, Fromm B, Gäde G, Predel R. A proteomic approach for studying insect phylogeny: CAPA peptides of ancient insect taxa (Dictyoptera, Blattoptera) as a test case. BMC Evol Biol. 2009; 9: 50. doi: 10.1186/1471-2148-9-50. PMID: 19257902; PMCID: PMC2667406.

[89] Grandcolas P. The phylogeny of cockroach families: a cladistic appraisal of morpho-anatomical data. Can J Zool. 1996; 74(3): 508-527. doi: 10.1139/z96-059.

[90] Lo N, Bandi C, Watanabe H, Nalepa C, Beninati T. Evidence for cocladogenesis between diverse dictyopteran lineages and their intracellular endosymbionts. Mol Biol Evol. 2003; 20(6): 907-13. doi: 10.1093/molbev/msg097. PMID: 12716997.

[91] Ware JL, Litman J, Klass KD, Spearman LA. Relationships among the major lineages of Dictyoptera: the effect of outgroup selection on dictyopteran tree topology. Syst Entomol. 2008; 33(3): 429-450.

[92] Misof B, Liu S, Meusemann K, Peters RS, Donath A, Mayer C, et al. Phylogenomics resolves the timing and pattern of insect evolution. Science. 2014; 346(6210): 763-7. doi: 10.1126/science.1257570. Epub 2014 Nov 6. PMID: 25378627.

[93] Pellens R, D'Haese CA, Bellés X, Piulachs MD, Legendre F, Wheeler WC, et al. The evolutionary transition from subsocial to eusocial behaviour in Dictyoptera: phylogenetic evidence for modification of the "shift-in-dependent-care" hypothesis with a new subsocial cockroach.

Mol Phylogenet Evol. 2007; 43(2): 616-26. doi: 10.1016/j.ympev.2006.12.017. PMID: 17291786.

Supporting Information

S1 Table. GenBank accession numbers for divergence date analyses.

S2 Table. GenBank accession numbers for taxas used in this study.

S3 Table. Nucleotide composition in different regions of *Episymploce splendens.*

S4 Table. Relative synonymous codon usage (RSCU) for PCGs of *Episymploce splendens.*

S5 Table. The length and similarity of these large intergenic spacers with their original genes in some insects. The similarity was calculated by DNAMAN.

S1 Fig. Putative secondary structures of the 20 tRNA genes identified in *Episymploce splendens.*

S2 Fig. Aligement result of the long intergenic spacer and srRNA of *Episymploce splendens* by MEGA.

S3(a) Fig. Maximum likelihood (ML) phylogenetic tree based on the amino acid sequences of 13 PCGs. Numbersabutting branches refer to bootstrap proportions (BSP). *Papilio protenor* and *Biston panterinaria* were employed to root the tree as outgroups.

S3(b) Fig. Bayesian phylogenetic tree based on the nucleotide sequences of 13 PCGs. Numbers abutting branches refer to Bayesian

posterior probabilities (BPP). *Papilio protenor* and *Biston panterinaria* were employed to root the tree as outgroups.

S3(c) Fig. Bayesian phylogenetic tree based on the amino acid sequences of 13 PCGs. Numbers abutting branches refer to Bayesian posterior probabilities (BPP). *Papilio protenor* and *Biston panterinaria* were employed to root the tree as outgroups.

S4 Fig. Similarity comparison results of Numt-1 and mito-genome of *Episymploce splendens* by DNAMAN.

S5 Fig. Similarity comparison results of Numt-2 and mito-genome of *Episymploce splendens* by DNAMAN.

Fig 1

Click here to access/download;Figure;Fig 1..tiff

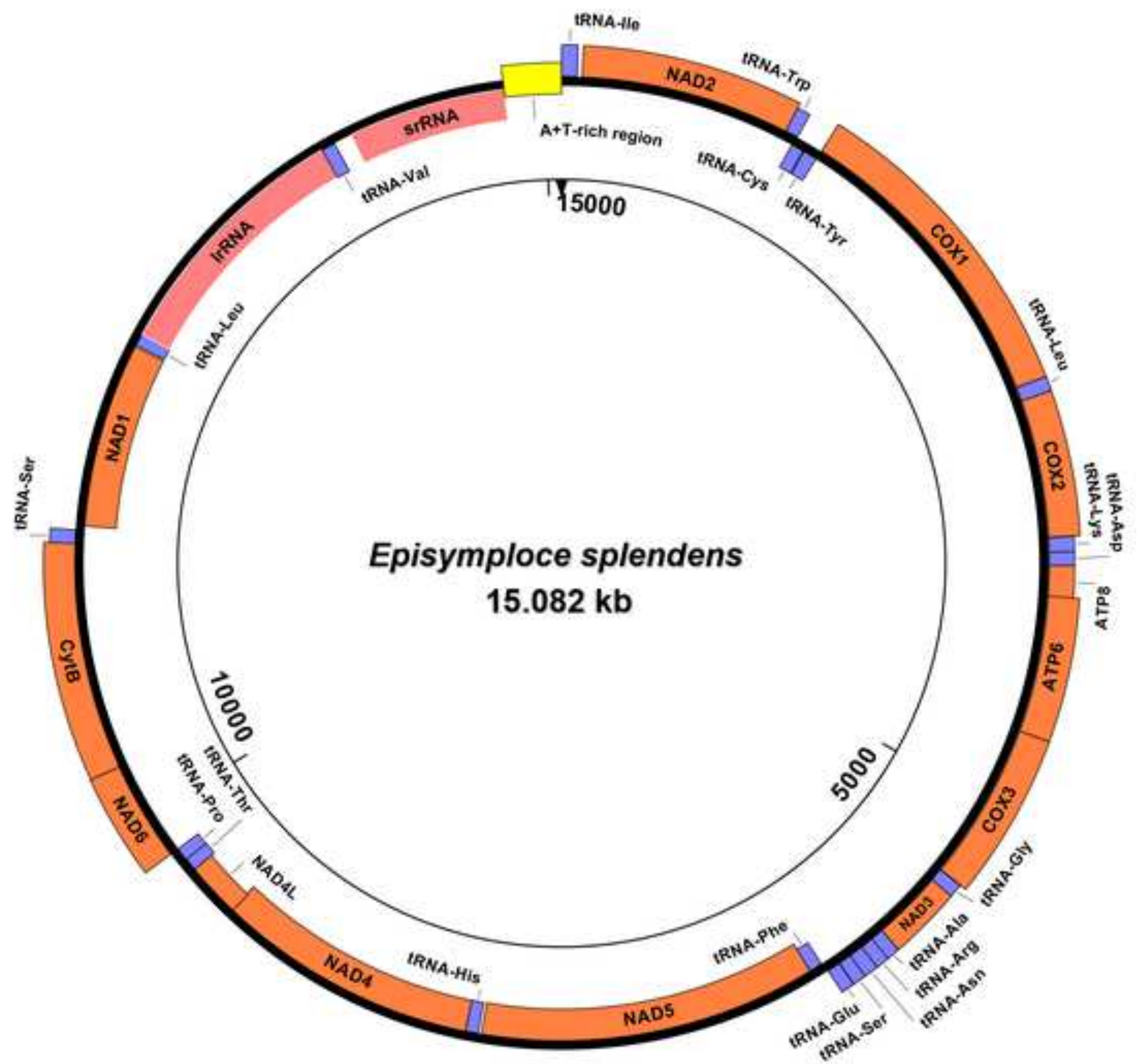

Fig 2

Click here to access/download;Figure;Fig 2..tif

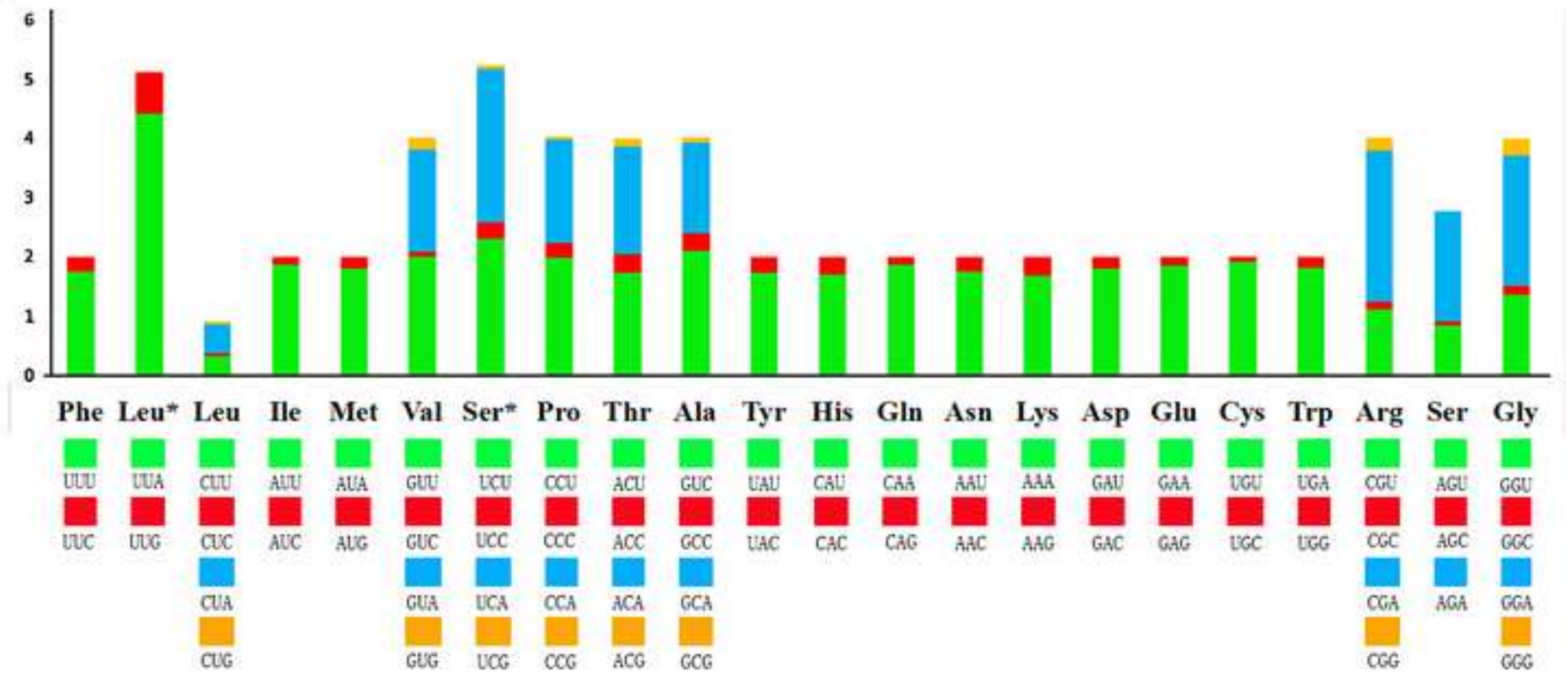

Fig 3

Click here to access/download;Figure;Fig 3..tif

TACCTAAAAATAAAGAGTGCCCCTGTCCCCCACTA

AACTATGATTTTACCTTACCTAAAAATAAAATAAG

TACAACTTCCTGCCCTTGTCCAATTTTCAACCTTTTC

AACCTTTTCAACCTTTTCAACCTAAATAAAAAATA

CCGTCCCTTGTCCCCCACTGAACTATGATTTTACCT

TACCTAAAAATAAAATAAGTACAACTTCCTGCCCT

TGTCCAATTTTCAACCTTTTCAACCTTTTCAACCTTT

TCAACCTAAATAAAAAATACCGTCCCTTGTCCAAT

CACCA

Fig 4

Click here to access/download;Figure;Fig 4..tif

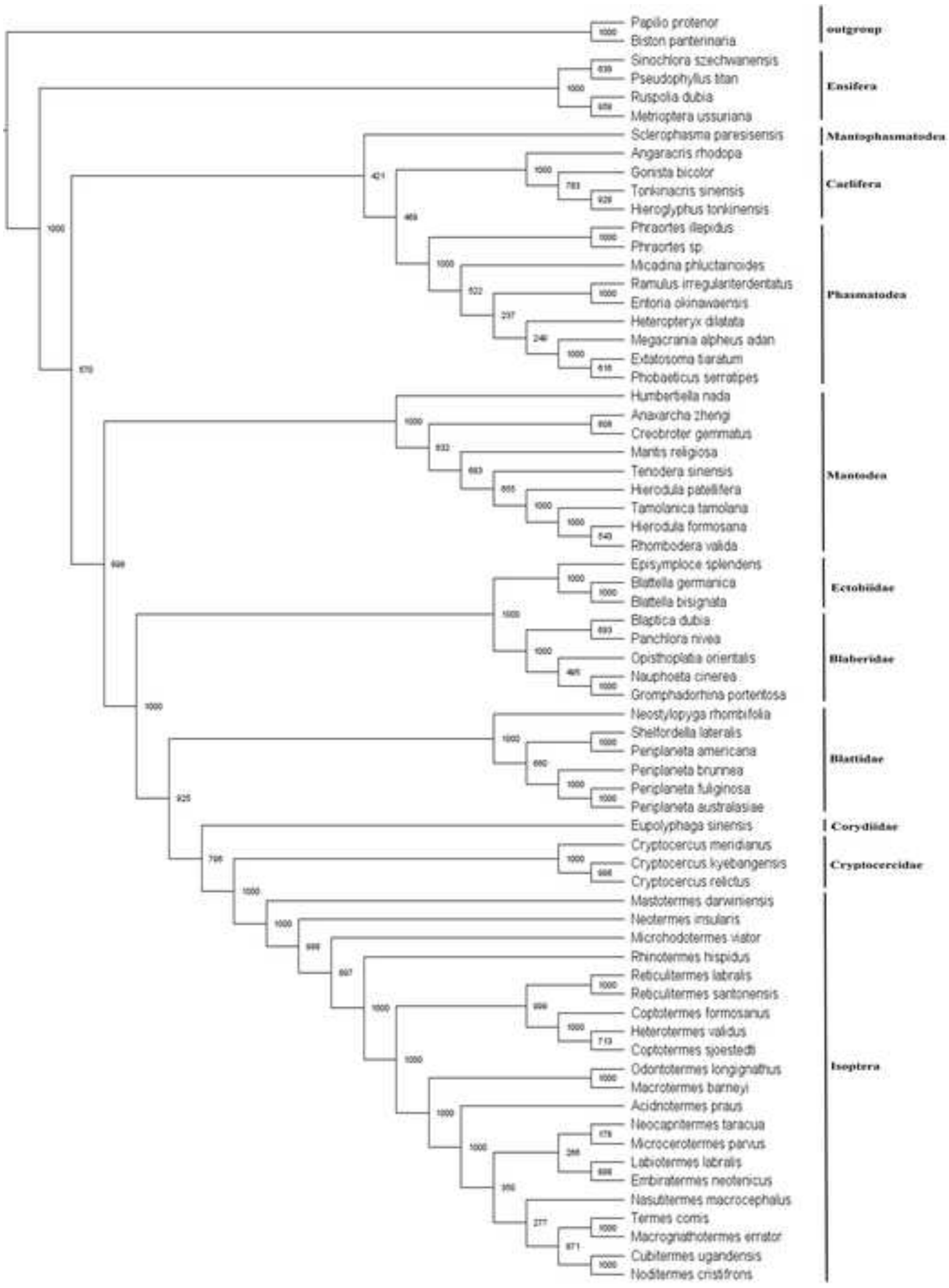

Fig 5 (a)

Click here to access/download;Figure;Fig 5 (a)..tif

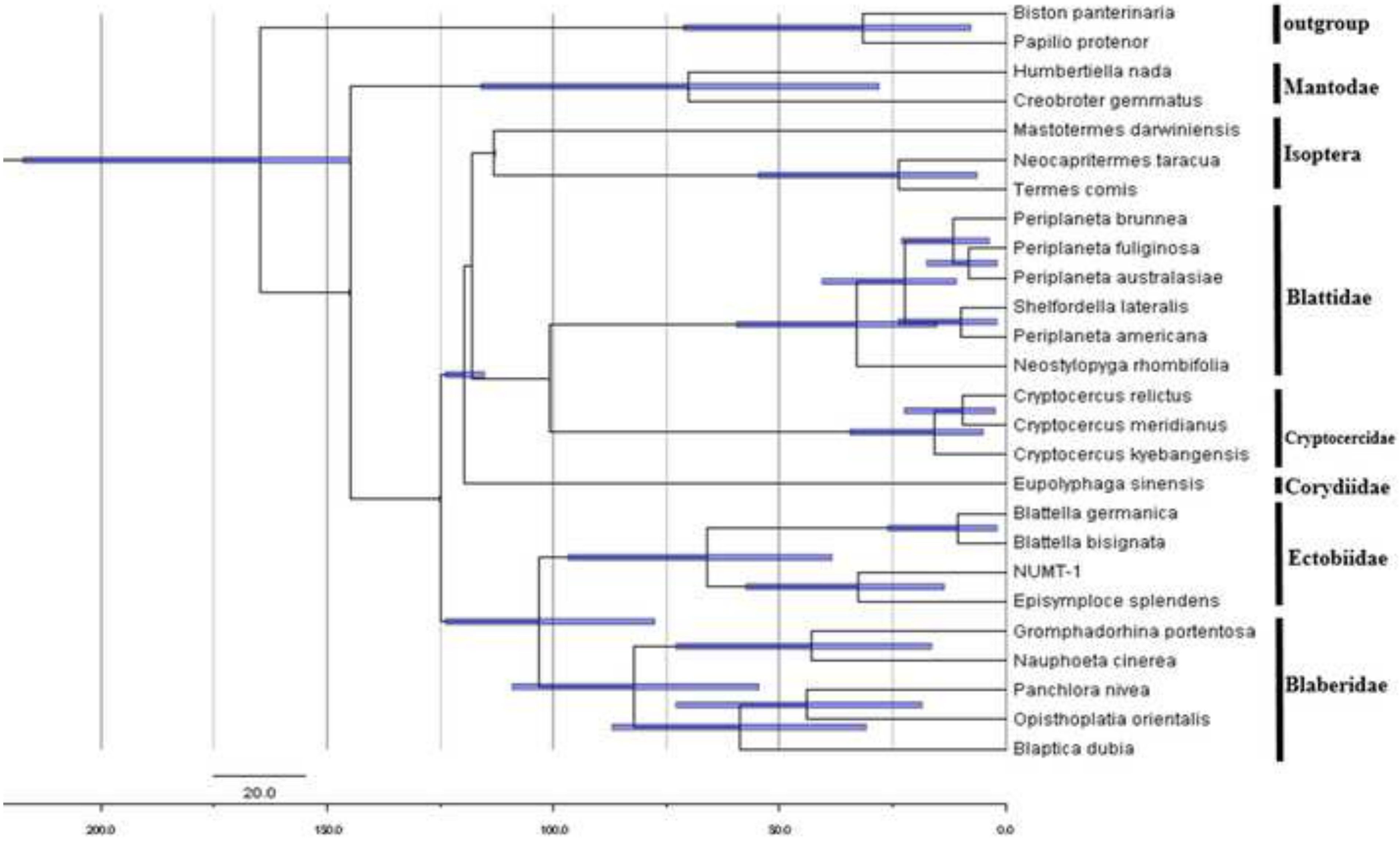

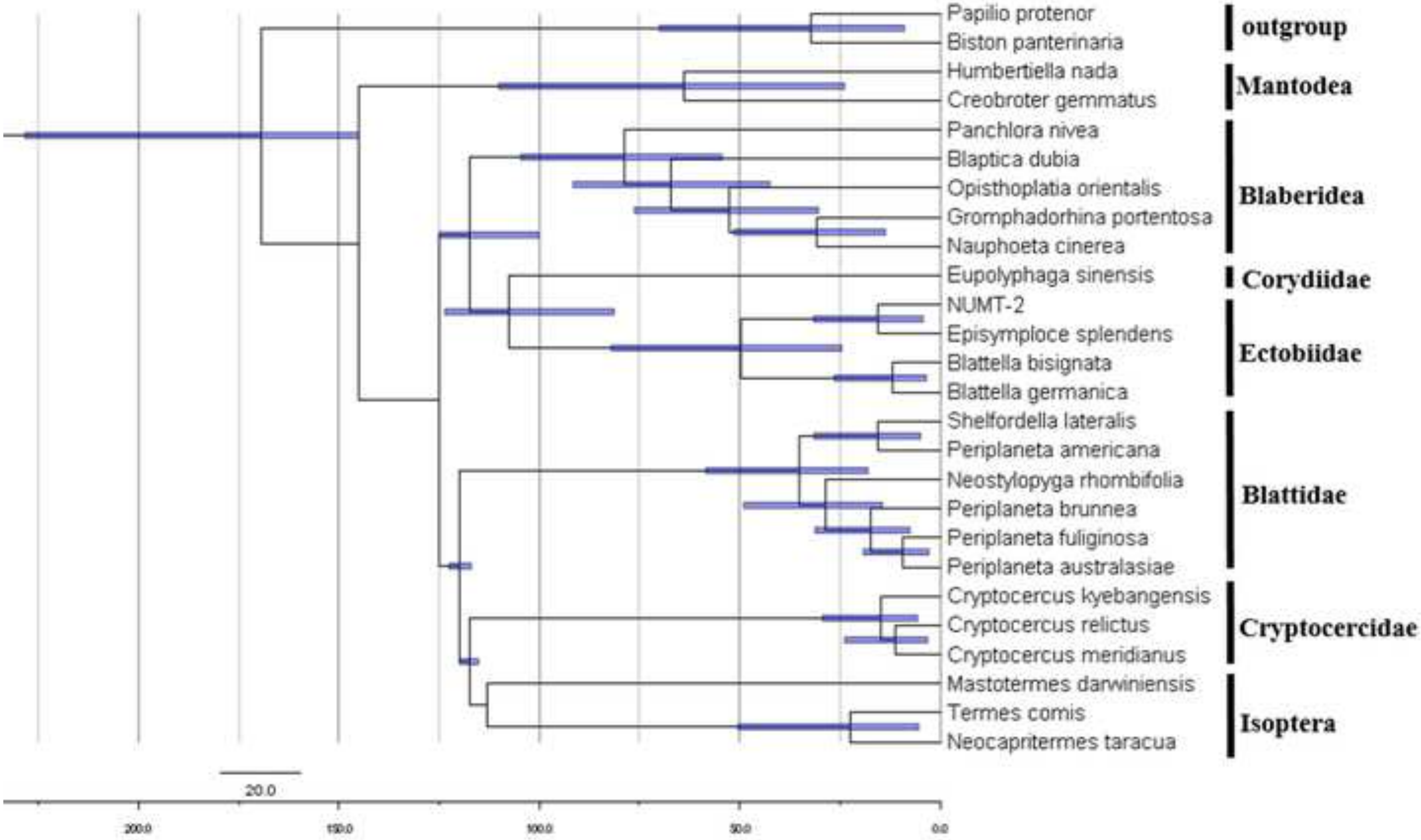
Papilio protenor
Biston panterinaria
Humbertiella nada
Creobroter gemmatus
Panchlora nivea
Blaptica dubia
Opisthoplatia orientalis
Gromphadorhina portentosa
Nauphoeta cinerea
Eupolyphaga sinensis
NUMT-2
Episymploce splendens
Blattella bisignata
Blattella germanica
Shelfordella lateralis
Periplaneta americana
Neostylopyga rhombifolia
Periplaneta brunnea
Periplaneta fuliginosa
Periplaneta australasiae
Cryptocercus kyebangensis
Cryptocercus relictus
Cryptocercus meridianus
Mastotermes darwiniensis
Termes comis
Neocapritermes taracua
outgroup
Mantodea
Blaberidea
Corydiidae
Ectobiidae
Blattidae
Cryptocercidae
Isoptera
20.0
200.0
150.0
100.0
50.0
0.0

Fig 6

Click here to access/download;Figure;Fig 6..tif

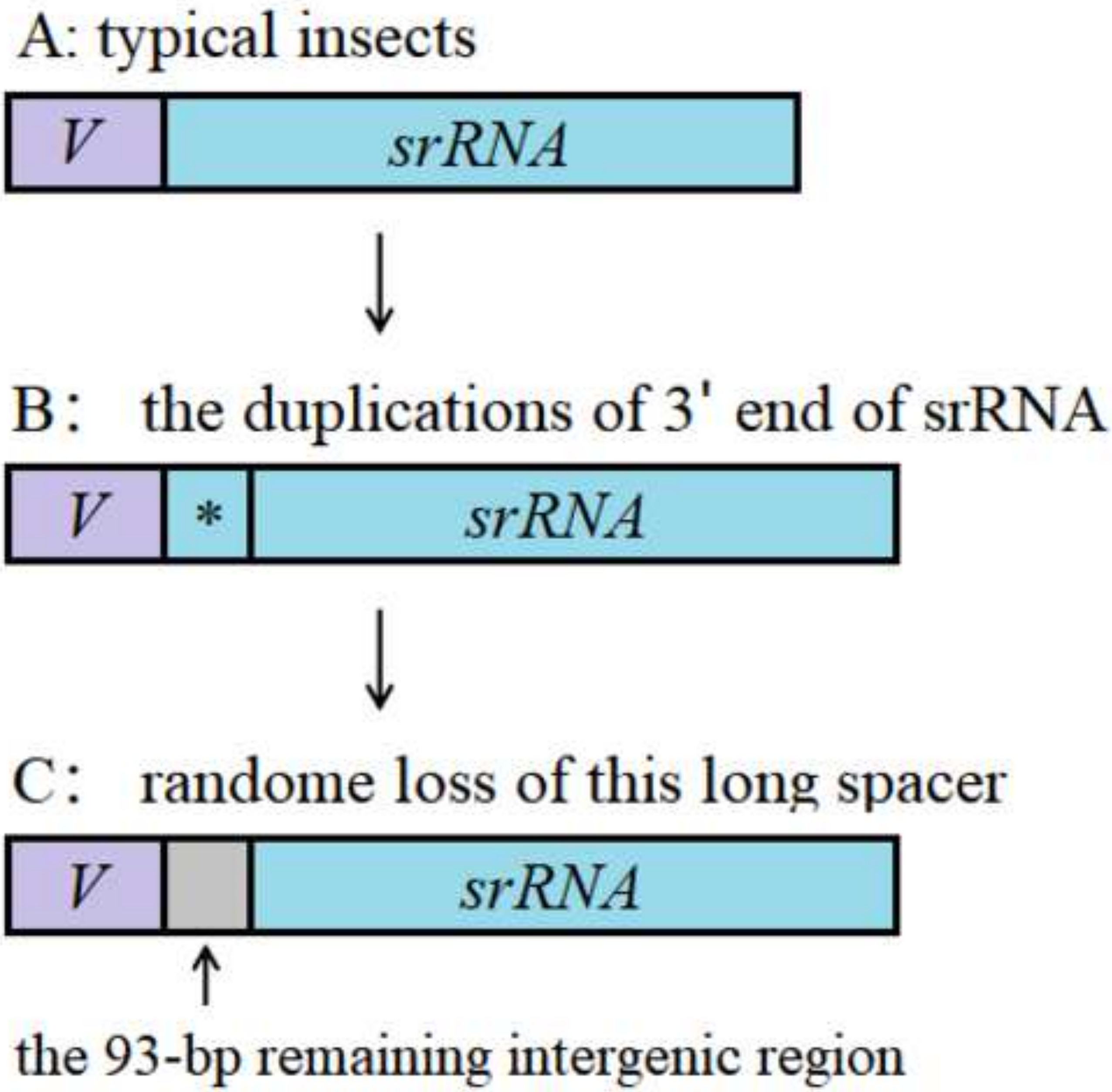

Click here to access/download
Supporting Information - Compressed/ZIP File Archive
Supporting Information.zip
