# Supplementary material for: Complete mitochondrial genome of Episymploce splendens (Blattodea: Ectobiidae): A large intergenic spacer and lacking of two tRNA genes
Source: PLoS One. 2022 Jun 2;17(6):e0268064. doi: 10.1371/journal.pone.0268064 (PMC9162313; doi:10.1371/journal.pone.0268064)
Supplement: Attachment — Submitted filename: PONE-D-21-15397_R3_reviewer.pdf [file pone.0268064.s013.pdf]

PLOS ONE

Complete mitochondrial genome of Episymploce splendens (Blattodea: Ectobiidae): a large intergenic spacer and lacking of two tRNA genes

--Manuscript Draft--

**Manuscript Number:**	PONE-D-21-15397R3
**Article Type:**	Research Article
**Full Title:**	Complete mitochondrial genome of Episymploce splendens (Blattodea: Ectobiidae): a large intergenic spacer and lacking of two tRNA genes
**Short Title:**	Complete mitochondrial genome of Episymploce splendens (Blattodea: Ectobiidae)
**Corresponding Author:**	Xiuyue Zhang<br>Sichuan University<br>Chengdu, CHINA
**Keywords:**	Episymploce splendens, mito-genome, tRNAs lacking, Large intergenic spacer, Numts, Phylogenetic analysis
**Abstract:**	The complete mitochondrial genome of Episymploce splendens, 15,802 bp in length, was determined and annotated in this study. The mito-genome included 13 PCGs, 20 tRNAs and 2 rRNAs. Unlike most typical mito-genomes with conservative gene arrangement and exceptional economic organization, E. splendens mito-genome has two tRNAs (tRNA-Gln and tRNA-Met) absence and a long intergenic spacer sequence (93 bp) between tRNA-Val and srRNA, showing the diversified features of insect mito-genomes. This is the first report of the tRNAs deletion in Orthopteroidea mito-genomes and we supported the duplication/random loss model as the origin mechanism of the long intergenic spacer. Two Numts, Numt-1 (557 bp) and Numt-2 (975 bp) transferred to the nucleus at about 14.15 Ma to 22.34 Ma, and 19.19 Ma to 24.06 Ma respectively, were found in E. splendens. They can be used as molecular fossils in insect phylogenetic relationship inference. Our study provided useful data for further studies on the evolution of insect mito-genome.
**Order of Authors:**	Lin Yan
	Zhenzhen Hou
	Jinnan Ma
	Hongmei Wang
	Jie Gao
	Chenjuan Zeng
	Qin Chen
	Bisong Yue
	Xiuyue Zhang
**Response to Reviewers:**	Response to Reviewers<br>Dear editor:<br>Thank you for giving us the opportunity to revise our manuscript entitled “Complete mitochondrial genome of Episymploce splendens (Blattodea: Ectobiidae): a large intergenic spacer and lacking of two tRNA genes” to PLOS ONE. We appreciate that the reviewers’ valuable comments for our manuscript. These comments are very important for improving our manuscript. We have revised our manuscript according to the reviewers’ comments and also made other improvement for our manuscript. We do hope that our manuscript is appropriate and could be considered for publication in this journal.<br>This study was funded by the Special funds for central government to guide local scientific and Technological Development (2020ZYD098) and National Natural Science Foundation of China (U21A20409).The funders had no role in study design, data collection and analysis, decision to publish, or preparation of the manuscript.<br><br>Best regards,

Powered by Editorial Manager® and ProduXion Manager® from Aries Systems Corporation

Sincerely yours,
Xiuyue Zhang, PhD and Professor
College of Life Sciences, Sichuan University
Email: zhangxy317@126.com

Reviewers' comments:
Reviewer 2:
This round you responded all the comments carefully, and deleted the meaningless or not closely related part. it feels much more compact and focused. I suggest emphasizing the existence of pseudogenes (numts) and the time when pseudogenes appear in this cockroach, instead of a large intergenic spacer. And I think the introduction to the study of the cockroach mitochondrial genome is too simplistic.

Response:
Thanks for your suggestion. The mito-genome commonly displays exceptional economy of organization and the large intergenic spacer was uncommon. We believed the find of large intergenic spacer in mitochondrial genome of Episymploce splendens was important for the study of insect mitochondrial genome evolution in the future and so we retained it. And based on your suggests, we also further emphasized the discovery of numts and their significance, and added a description of the mitochondrial genome and enriched the content.

Reviewer 3:
1.I failed to remember that I have reviewed this paper before. I failed to find this mt genome of OK094023 in NCBI. I need this sequence to judge the mt genome right or not! If I reviewed it before, I must be concerned on this question. I did not believe two trnQ and trnM lost in Episymploce splendens.
The two trnQ f and trnM can be translated into between two CRs. As the authors said that this is the first report of the tRNAs deletion in Orthopteroidea mito-genomes. But I did not believe it. I suggest the authors check it carefully using the other species in genus Episymploce. Because if the mt genome is existed two similar CRs, the lost genes will be found between two CRs.
I suggest the authors should be carefully checked again.
When the authors used a pair of primers of "Es11 13822-CAGATTATATTGATTCGCACAAC" and "303-ATAGAACTGATGAAGCTAAGGC" to amplify PCR using 75 second Extension. It may be failed to amplify two control regions. I suggest the authors can design more primers to check it.
Response:
Thanks for your careful reading.
We really appreciate your valuable time on reviewing our manuscript. We had provided the complete mt-genome to Genbank and obtained the GenBank identifier (OK094023) but we set up the release date, and so this sequence cannot be found in NCBI at present. However, in order to the convenience of review, it has been uploaded as an attachment in our submitted manuscript and also in our revised manuscript.
The non-coding region of E. splendens mito-genome was located between srRNA and tRNA-Ile, and it was 290 bp long consist of two 120 bp long repeated units. The two lost tRNAs were located behind tRNA-Ile, and the interval between tRNA-Ile and NAD2 was only 24bp. They cannot form the 2 tRNAs. Because we used ARWEN and tRNA-SE to identify tRNAs and obtained same result through reference to secondary structure models for those genes from other blattarian insects (Ma et al., 2017; Xiao et al., 2012). The tRNA-Gln and tRNA-Met located between tRNA-Ile and NAD2, and they were typically 150bp long in other cockroaches. However, this site was only 24bp long in E. splendens. In addition, we tried to refer to other cockroach mitochondrial genomes, but however, there was no mitochondrial sequences reported in Episymploce in NCBI. In reported cockroach mitochondrial genomes, we did not find two similar CRs, namely, these reported cockroach mitochondrial genomes have only one A-T region. We designed different primers (in the table) to amplify this area and obtain same amplification sequences, so we think our experiment result was correct and there was no sequence lost due to inappropriate primers. We could not found the secondary structures of two tRNAs in E. splendens mito-genome sequence, and so we believed their loss event. Although tRNA lacking was rare, other species had been found in previous studies. For example, Limnonectes bannaensis mt-genome lost 4 tRNAs (tRNA-Ala, tRNA-Asn, tRNA-Cys and tRNA-Glu), Gegeneophis ramaswamii mt-genome lost tRNA-Phe, Urochela quadrinotata Reuter mt-genome lost 2 tRNAs (tRNA-

	Ile and tRNA-Gln), Platysternon megacephalum mt-genome lost tRNA-Thr. These loss events of tRNAs occur accidentally and independently in these mt-genome, and we also believe that these findings will be useful for future studies on the cause of their loss and the subsequent adaptive evolution.<br>FR<br>1.ES11CAGATTATATTGATTCGCACAACATAGAACTGATGAAGCTAAGGC<br>2.(Ma et al,2017)CCTCTAAAAAGACTAAAATACCGCCGGAATCATCAGTGAAAGGGAGC<br>3.AATAAAGAGTGCCCCTGTCGATATTACAGGGATGAAGGATA<br>4.TAAAGAGTGCCCCTGTCTGATGAAGCTAAGGCTTGAA<br>2. Two Numts were found by PCR. Two Numts were similar to lrRNA, which can be explained why the author failed to obtained trnQ and trnM. Check the mt genome again!<br>Another question is how the authors judge the two Numts？ Why is not lrRNA belonging to Numt？<br>I did think the divergence time of Numts are interesting. Because the title is "Complete mitochondrial genome of Episymploce splendens (Blattodea: Ectobiidae): a large intergenic spacer and lacking of two tRNA genes" not including Numts.<br>Response:<br>The sequences which originated from the invasion of mtDNA into nuclear DNA were named Numts. Numts exist in the nuclear DNA and do not belong to the mitochondrial genome sequence, but due to their partial sequences may be identical to their source sequences, some primers that amplify mitochondrial genomes may accidentally amplify these sequences. So, two Numts may be not related to the loss of tRNAs.<br>Primers that amplified mitochondrial genes may occasionally amplify Numts. In this study, agarose gel electrophoresis of the amplification product (the primer Numt-1-1 and Numt-2-1, Table 1) appeared as two bands. We then redesigned primers (Numt-1-2 and Numt-2-2, Table 1) to amplify these regions and obtained the same result. These non-targeted bands were purified and sequenced, and obtained sequences belonging to mitochondria via the analysis of NCBI blast. Due to differences in mutation rates or relaxation from selection pressure, they had some degree of base differences compared to corresponding mito-genome sequences. These sequences have more mutations of insertion and deletion compared to their corresponding mitochondrial sequences, and may lose their functions. They did not belong to the mitochondrial genome, so they were not the lost tRNAs. We had explained this in the corresponding section of the manuscript (Page line 162). Numts can be used to solve problems in phylogeny, but they were not a part of the mitochondria, so we did not mentioned them in the title.<br><br>3.Is a large intergenic spacer similar to trnI or trnQ? or AT-rich region?<br>Response:<br>The large intergenic spacer was not A-T region or similar to trnI or trnQ. It was similar to its corresponding srRNA with a 64.6% similarity.<br><br>4.I read the paper and found the English writing is really poor. There are many mistaken in the paper. I can not believe the paper is revsied the second version.<br>Response:<br>we're sorry for the writing mistaken and we have made careful revisions to our English writing.<br>5.All genes name should be the same in text, figures, and tables.<br>Response:<br>We have made relevant changes in our revised manuscript.
**Additional Information:**	
**Question**	**Response**
**Financial Disclosure**<br><br>Enter a financial disclosure statement that describes the sources of funding for the work included in this submission. Review the submission guidelines for detailed requirements. View published research	This study was funded by the Science and Technology Project of Sichuan Province (2017SZ0019), Special funds for central government to guide local scientific and Technological Development (2020ZYD098) and State Administration of Traditional Chinese Medicine of the P.R.C (ZYBZH-C-SC-53).

Powered by Editorial Manager® and ProduXion Manager® from Aries Systems Corporation

articles from *PLOS ONE* for specific examples.<br><br>This statement is required for submission and **will appear in the published article** if the submission is accepted. Please make sure it is accurate.<br><br>**Unfunded studies**<br>Enter: *The author(s) received no specific funding for this work.*<br><br>**Funded studies**<br>Enter a statement with the following details:<br>• Initials of the authors who received each award<br>• Grant numbers awarded to each author<br>• The full name of each funder<br>• URL of each funder website<br>• Did the sponsors or funders play any role in the study design, data collection and analysis, decision to publish, or preparation of the manuscript?<br>• **NO** - Include this sentence at the end of your statement: *The funders had no role in study design, data collection and analysis, decision to publish, or preparation of the manuscript.*<br>• **YES** - Specify the role(s) played.<br><br>* typeset	
**Competing Interests**<br><br>Use the instructions below to enter a competing interest statement for this submission. On behalf of all authors, disclose any competing interests that could be perceived to bias this work—acknowledging all financial support and any other relevant financial or non-financial competing interests.<br><br>This statement is required for submission and **will appear in the published article** if the submission is accepted. Please make sure it is accurate and that any funding sources listed in your Funding Information later in the submission form are also declared in your Financial Disclosure statement.	The authors have declared that no competing interests exist.

Powered by Editorial Manager® and ProduXion Manager® from Aries Systems Corporation

View published research articles from *PLOS ONE* for specific examples.<br><br>**NO authors have competing interests**<br><br>Enter: *The authors have declared that no competing interests exist.*<br><br>**Authors with competing interests**<br><br>Enter competing interest details beginning with this statement:<br><br>*I have read the journal's policy and the authors of this manuscript have the following competing interests: [insert competing interests here]*<br><br>* typeset	
**Ethics Statement**<br><br>Enter an ethics statement for this submission. This statement is required if the study involved:<br><br>• Human participants<br>• Human specimens or tissue<br>• Vertebrate animals or cephalopods<br>• Vertebrate embryos or tissues<br>• Field research<br><br>Write "N/A" if the submission does not require an ethics statement.<br><br>General guidance is provided below. Consult the submission guidelines for detailed instructions. **Make sure that all information entered here is included in the Methods section of the manuscript.**	Episymploce splendens is a cockroach. It's not a vertebrate, and it's not a protectanimal. We stun the insects with ether and then put them into anhydrous ethanol to die, and take fresh materials for subsequent experiments.

Powered by Editorial Manager® and ProduXion Manager® from Aries Systems Corporation

**Format for specific study types**<br><br>**Human Subject Research (involving human participants and/or tissue)**<br>• Give the name of the institutional review board or ethics committee that approved the study<br>• Include the approval number and/or a statement indicating approval of this research<br>• Indicate the form of consent obtained (written/oral) or the reason that consent was not obtained (e.g. the data were analyzed anonymously)<br><br>**Animal Research (involving vertebrate animals, embryos or tissues)**<br>• Provide the name of the Institutional Animal Care and Use Committee (IACUC) or other relevant ethics board that reviewed the study protocol, and indicate whether they approved this research or granted a formal waiver of ethical approval<br>• Include an approval number if one was obtained<br>• If the study involved *non-human primates*, add *additional details* about animal welfare and steps taken to ameliorate suffering<br>• If anesthesia, euthanasia, or any kind of animal sacrifice is part of the study, include briefly which substances and/or methods were applied<br><br>**Field Research**<br><br>Include the following details if this study involves the collection of plant, animal, or other materials from a natural setting:<br>• Field permit number<br>• Name of the institution or relevant body that granted permission	
**Data Availability**<br><br>Authors are required to make all data underlying the findings described fully available, without restriction, and from the time of publication. PLOS allows rare exceptions to address legal and ethical concerns. See the PLOS Data Policy and FAQ for detailed information.	Yes - all data are fully available without restriction

A Data Availability Statement describing where the data can be found is required at submission. Your answers to this question constitute the Data Availability Statement and **will be published in the article**, if accepted.<br><br>**Important:** Stating 'data available on request from the author' is not sufficient. If your data are only available upon request, select 'No' for the first question and explain your exceptional situation in the text box.<br><br>Do the authors confirm that all data underlying the findings described in their manuscript are fully available without restriction?	
**Describe where the data may be found in full sentences. If you are copying our sample text, replace any instances of XXX with the appropriate details.**<br><br>• If the data are **held or will be held in a public repository**, include URLs, accession numbers or DOIs. If this information will only be available after acceptance, indicate this by ticking the box below. For example: *All XXX files are available from the XXX database (accession number(s) XXX, XXX.)*.<br>• If the data are all contained **within the manuscript and/or Supporting Information files**, enter the following: *All relevant data are within the manuscript and its Supporting Information files.*<br>• If neither of these applies but you are able to provide **details of access elsewhere**, with or without limitations, please do so. For example:<br><br>*Data cannot be shared publicly because of [XXX]. Data are available from the XXX Institutional Data Access / Ethics Committee (contact via XXX) for researchers who meet the criteria for access to confidential data.*<br><br>*The data underlying the results presented in the study are available from (include the name of the third party*	***********NOTE TO PA AT ACCEPT: Please ask for accession numbers to data at accept*************<br><br>Yes - all data are fully available without restriction; All sequences are awaited to upload to NCBI Genbank database.

and contact information or URL).<br>• This text is appropriate if the data are owned by a third party and authors do not have permission to share the data.<br><br>* typeset	
Additional data availability information:	

Powered by Editorial Manager® and ProduXion Manager® from Aries Systems Corporation

Manuscript Click here to access/download;Manuscript;manuscrip.docx

Complete mitochondrial genome of *Episymploce splendens* (Blattodea: Ectobiidae): a large intergenic spacer and lacking of two tRNA genes

Lin Yan[1], Zhenzhen Hou[2], Jinnan Ma[1], Hongmei Wang[1], Jie Gao[3], Chenjuan Zeng[3], Qin Chen[1], Bisong Yue[1], Xiuyue Zhang[1,2]*

1Key Laboratory of Bio-resources and Eco-environment, Ministry of Education, College of Life Sciences, Sichuan University, No.24 South Section 1, Yihuan Road, Chengdu, China, 610065.

2Sichuan Key Laboratory of Conservation Biology on Endangered Wildlife, College of Life Sciences, Sichuan University, No.24 South Section 1, Yihuan Road, Chengdu, China, 610065.

3Sichuan Key Laboratory of Medicinal Periplaneta americana, Xichang, Sichuan Province 615000, China.

*E-mail: zhangxiuyue@scu.edu.cn

Abstract

The complete mitochondrial genome of *Episymploce splendens*, 15,802 bp in length, was determined and annotated in this study. The mito-genome included 13 PCGs, 20 tRNAs and 2 rRNAs. Unlike most typical mito-genomes with conservative gene arrangement and exceptional economic organization, *E. splendens* mito-genome has two tRNAs (tRNA-Gln and tRNA-Met) absence and a long intergenic spacer sequence (93 bp) between tRNA-Val and srRNA, showing the diversified features of insect mito-genomes. This is the first report of the tRNAs deletion in Blattarian mito-genomes and we supported the duplication/random loss model as the origin mechanism of the long intergenic spacer. Two Numts, Numt-1 (557 bp) and Numt-2 (975 bp) transferred to the nucleus at about 14.15 Ma to 22.34 Ma, and 19.19 Ma to 24.06 Ma respectively, were found in *E. splendens*. They can be used as molecular fossils in insect phylogenetic relationship inference. Our study provided useful data for further studies on the evolution of insect mito-genome.

Introduction

The mitochondrial genome has the characteristics of maternal inheritance, rare genetic recombination, faster evolution rate and conservative gene arrangement [1]. The mito-genome commonly displays

exceptional economy of organization, with overlapping genes or small intergenic spacers [2]. A typical insect mito-genome, with a compact circular molecule, generally encodes a fixed set of 37 genes including 13 protein-coding genes (PCGs), 22 transfer RNA (tRNA) genes, two ribosomal RNA (rRNA) genes and a control region [3]. However, exceptions have been reported, such as long gene intergenic spacer [4 -6], gene rearrangement and gene loss [7-8]. So far, mitochondrial gene rearrangement has been reported in about 17 orders of insects, and these genes include protein coding gene, rRNA gene and tRNA gene [8]. In addition tRNAs, the missing protein coding genes have also been reported in Mantodea, Phthiraptera, and Psocoptera[9-13]. The tRNAs are most various in no-typical insect mito-genomes, including tRNA translocation, tRNA loss, tRNA tandem duplication and tRNA conversion [4,9-10,14-15]. Phthiraptera and Psocoptera insect mito-genomes may be most multipartite and most fast in evolution, that they could fission into fragmented mito-genomes and have numerous pseudo-genes and diverse gene rearrangement [7-10]. Thus, insect mitochondrial genomes are good models for researching mitochondrial genome evolution.

There are of 4,600 species of cockroaches [16] and 300 mitochondrial genomes of them have been sequenced and uploaded to the NCBI database. The Blattarian mito-genomes seem to be in a conserved evolution [16-17]. The gene rearrangement was only reported in

Cryptocercidae within Blattarian mito-genomes [4]. *Episymploce splendens* belongs to Ectobiidae and at present, no complete mitochondrial sequences of *Episymploce* have been recorded in the NCBI database. Previous studies have mainly focused on the morphological features of *Episymploce* rather than molecular data [18-19]. In this study, we sequenced, annotated and described the complete mito-genome of *E. splendens*. Two tRNAs loss and a long intergenic region was found in *E. splendens* mito-genome and two pseudo-genes were also identified in the study. This study could deepen our understanding of mitochondrial genome of insects and contribute to the study of insect mito-genome evolution.

Materials and Methods

Sample and DNA extraction

The *Episymploce* species have typical characteristics of adult cockroaches such as two sets of wings, sclerotized tegmina and wider membranous hindwings [18]. Besides, the pronotum of *E. splendens* is black and one-orange patch is on each side of the lateral border. The front and hind wings are fully developed and through the end of the abdomen. Supra-anal plate of male genitalia is asymmetrical.

The cockroaches used in this study were collected on Mount Emei, Sichuan Province, China. The fresh material was placed in absolute ethanol and stored at -20 ℃. Total genomic DNA was extracted from the

muscle tissue (legs) using TIANamp Genomic DNA kit (TIANGEN, Beijing, China). The concentration and purity of total DNA were detected by spectrophotometer. Firstly, 2 μl DNA elution solution was taken to calibrate the instrument. Then, the spot sample hole was wiped clean and 2 μl DNA was taken to determine the concentration and purity of the test sample. In addition, DNA was detected by agarose gel electrophoresis, and 1% agarose gel electrophoresis judged whether DNA was successfully extracted or not. Finally, DNA was stored at -20 ℃.

PCR amplification and sequencing

Primers were designed based on the conserved sequences of *Blattella germanica* and *Blattella bisignata*, and then specific primers were designed based on the amplified and sequenced sequences at both ends [20-21]. The software Primer Premier 5.0 was used to designed primers and the primer details are listed in Table 1. Primers Es3, Es9 and Es10 were obtained from Xiao *et al* [10]. PCR was conducted using 2×Taq PCR Mix (Innovagene, Chengdu, China) and performed on a PTC-100 thermal cycler (BioRad, Hercules, CA) with the following cycling conditions: an initial denaturation for 5 min at 94℃, followed by 35 cycles of denaturation for 30s at 94℃, annealing for 30s at 50-62℃ (depending on primer combinations), elongation for 1-4 min (depending on putative length of the fragments) at 72℃, and a final extension step of 72℃ for 10 min. PCR products were estimated by 1.0% agarose gel

electrophoresis and sent to Tsingke Biotechnology Company (Chengdu, China) for sequencing. All fragments were bidirectional sequences, and the unsuccessful sequenced fragments were redesigned with primers and sequenced again to complete the sequence.

Table 1. Primers for the PCR amplifications of *Episymploce splendens* (Es) mito-genome and Numts.

Primer name	Upstream primers sequences(5'-3')	Downstream primers sequences(5'-3')	Anneal temperature (℃)	Extension time (Second)
Es1	140-CCTCTCTTATCGCAATGTCCA	1609-CGTGGGAAAGCTATATCAGGA	58	90
Es2	1367-GGTCAACAAATCATAAAGATATTGG	2074-TAAACTTCAGGGTGACCAAAAAATCA	55	45
Es3	1559-CAACATTTATTTTGATTCTTTGG	3649-GTTTAAGAGACCACCACTTG	50	90
Es4	3401-GAAGACTTTCACCAACCATC	4391-TAGTACACTCATCTACTCTGGTAAC	52	60
Es5	4031-TGTAACAGCCCATGCTT	5797-AATCGCAATGATGGTAGG	51	110
Es6	4945-AGAAGACTTTCACCAACCAT	6997-GGATTCTCAAGATATTCGTT	51	120
Es7	6378-TCAACCGTTATCGAAAGACT	7328-CTCCTACTCCTGTATCTGCTT	52	60
Es8	7010-AACGAATATCCTGAGAATCC	8415-CACGGATTATGTTCTTCAGG	52	90
Es9	8290-GAAGGGGGTGCTGCTATATTAC	11151-ATTACTCCTCCTAATTTATTAGGAAT	62	180
Es10	10491-CAATGAGTATGAGGAGGATTTGCTGT	14755-TGTGCCAGCAGTCGCGGTTATACA	59.5	240
Es11	13822-CAGATTATATTGATTCGCACAAC	303-ATAGAACTGATGAAGCTAAGGC	55	75
Numt-1-1	GATTACGCTGTTATCCCTAAG	GGTGTAACTAGAATGATACAGGT	51	65
Numt-1-2	CGGTTTGAACTCAGATCATGTAAG	GAAGGTGTAACTAGAATGATACAGGT	57	78
Numt-2-1	TGCTACCTTTGCACGGTC	AGGTGAGATAAGTCGTAACATAGT	53	54
Numt-2-2	TAAACTCTATAGGGTCTTCTCG	CTAGAATGATACAGGTTAGGCT	53	70

The sequences which originated from the invasion of nuclear DNA by mtDNA were named Numts [22]. Primers that amplified mitochondrial genes may occasionally amplify Numts [23]. In this study, agarose gel electrophoresis of the amplification product (the primer Numt-1-1 and Numt-2-1, Table 1) appeared as two bands. We then redesigned primers (Numt-1-2 and Numt-2-2, Table 1) to amplify these regions and obtained the same result. These non-targeted bands were purified and sequenced, and obtained sequences belonging to mitochondria via the analysis of NCBI blast. Due to differences in mutation rates or freedom from selection pressure, they had some degree of base differences compared to corresponding mito-genome sequences.

Sequence analysis and annotation

DNA SeqMan program, which is included in the Lasergene software package (DNAStar Inc. Madison Wis), was used to assemble sequences to obtain the complete mitochondrial genome. PCGs and rRNAs were identified by comparing *E. splendens* to *B. germanica* and *B. bisignata* [20-21]. Most of the tRNA and their secondary structure inferences were conducted using the online server ARWEN (http://mbio-serv2.mbioekol.lu.se/ARWEN/) [24]. The tRNA-Ile, tRNA-Phe and tRNA-Leu were not identified by ARWEN; they were manually ~~recognized~~ by referring to secondary structural models of other blattaria insects. The mitogenomic map was depicted with SeqBuilder

(http://www.dnastar.com). The A+T content of the nucleotide sequence and relative synonymous codon usage (RSCU) were calculated using MEGA 5.2 [25]. The AT skewness was calculated according to the following formula: AT skew = [A-T]/[A+T], and the GC skewness was calculated according to the following formula: GC skew = [G-C]/[G+C] [26].

Divergence dating analysis

There were two Numts found in *E. splendens,* namely Numt-1 and Numt-2. Through sequence alignment by MEGA 5.2, Numt-1 corresponded to partial lrRNA, and Numt-2 was similar to partial lrRNA and its neighboring tRNA-Val of the *E. splendens* mito-genome. We performed divergence date analyses based on the aligned sequences of Blattodea, two mantises and two outgroups (S1 Table). The molecular clock was calibrated using three minimum age constraints based on cockroach fossils, as shown in Table 2. A relaxed molecular-clock model was used for this study with the program BEAST 1.6.1 [27]. Rate variation was modeled among branches using uncorrelated lognormal relaxed clocks [27]. A Yule speciation process was used for the tree prior and posterior distributions of parameters, including the tree, were estimated using MCMC sampling [28]. Two independent runs (each with 4 chains) of 100 million generations were sampled every 5000 generations based on the GTR model. The tree topology was then

estimated using the combined sample from the last 50 million generations of each run.

Table 2. Fossils used for estimation of divergence time of Numts in the analysis.

species	Age (Ma)	Calibration Group	Reference
Valditermes brennae	130.8	*Cryptocercus* + Isoptera	[29]
"Gyna" obesa	57.7	Blaberidae	[30]
Cratomastotermes wolfschwenningeri	113	termites	[31]

Results

Genome content and organization

The mitochondrial genome of *E. splendens* was 15,802 bp in length with typical circular molecules. It coded for 35 mitochondrial genes: 13 PCGs, 2 rRNAs (srRNA and lrRNA), 20 tRNAs and the A+T rich region (Fig 1, Table 3). Putative secondary structures of the 20 tRNAs were shown in Fig. S1. Similar to most species' mitochondria, the coding genes of *E. splendens* mito-genome are compact, with several genes overlapping. There were five overlaps totaling 21 bp with the two longest overlaps being 7 bp between *ATP8* and *ATP6*, and 7 bp between *NAD4* and *NAD4L*. There were 15 gene spacers totaling 190 bp within the entire mitochondrial genome. The longest spacer was 93 bp between tRNA-Val and srRNA, followed by 24 bp between tRNA-Ile and *NAD2* and 22 bp between *NAD1* and tRNA-Leu and it had a similarity of 64.6% compared

to its adjacent and corresponding srRNA (Fig. S2). There were 16 areas with neither gene overlap nor intergenic spacer. Additionally, there were 20 tRNAs in the mitochondrial genome of *E. splendens*, lacking two tRNAs located between tRNA-Ile and *NAD2*, tRNA-Gln and tRNA-Met (Fig 1).

Fig 1. Circular gene map of *Episymploce splendens* mito-genome. Genes coded in the J-strand are inside of the circle. Gene coded in the N-strand are outside of the circle. *COX1*, *COX2* and *COX3* refer to the cytochrome C oxidase subunits; *CytB* refers to cytochrome B; ATPase6 and ATPase8 refer to ATP synthase subunits 6 and 8 genes; and *NAD1-NAD6* and *NAD4L* refer to the NADH dehydrogenase subunit 1-6 and 4Lgenes.

Table 3. Annotation of *Episymploce splendens* mito-genome.

Gene	Grand	Location	Anticodon	Start codon	Stop codon
tRNA-Ile	J	1..77	TAT		
NAD2	J	102..1148		ATG	TAA
tRNA-Trp	J	1149..1212	TCA		
tRNA-Cys	N	1208..1271	GCA		
tRNA-Tyr	N	1278..1347	GTA		
COX1	J	1350..2885		TTG	TAA
tRNA-Leu(UUR)	J	2887..2954	TAA		
COX2	J	2956..3642		ATG	TAA
tRNA-Lys	J	3645..3715	CTT		
tRNA-Asp	J	3717..3781	GTC		
ATPase8	J	3782..3940		ATT	TAA
ATPase6	J	3934..4613		ATG	TA-
COX3	J	4614..5402		ATG	TAA
tRNA-Gly	J	5405..5468	TCC		

NAD3	J	5469..5821		ATG	TA-
tRNA-Ala	J	5822..5886	TGC		
tRNA-Arg	J	5885..5950	TCG		
tRNA-Asn	J	5951..6020	GTT		
tRNA-Ser(AGN)	J	6021..6085	GCT		
tRNA-Glu	J	6088..6152	TTC		
tRNA-Phe	N	6162..6228	GAA		
NAD5	N	6229..7947		ATT	TAA
tRNA-His	N	7963..8027	GTG		
NAD4	N	8028..9367		ATG	TA-
NAD4L	N	9361..9642		ATG	TAA
tRNA-Thr	N	9643..9709	TGT		
tRNA-Pro	N	9709..9774	TGG		
NAD6	J	9776..10275		ATT	TA-
CytB	J	10276..11409		ATG	TAA
tRNA-Ser(UCN)	J	11409..11477	TGA		
NAD1	N	11500..12441		ATA	TAG
tRNA-Leu(CUN)	N	12451..12511	TAG		
lrRNA	N	12512..13822			
tRNA-Val	N	13823..13893	TAC		
srRNA	N	13987..14792			
A+T-rich region		14793..15082			

'TA-' refer to incomplete stop codons.

Nucleotide composition and codon usage

We calculated the nucleotide composition of the mtDNA in *E. splendens* using MEGA5.2, refer to S2 Table for detailed results. The content of A+T (74.6%) was higher than G+C (25. 4%). It corresponded well to the AT bias generally observed in insect mito-genomes, which ranges from 69.5 to 84.9% [32-33].

The high A+T content and nucleotide skewness of the mitochondrial genome were also reflected in the codon use of protein-coding genes. According to the relative synonymous codon usage (RSCU) value (S3

Table, Fig 2), the occurrence of synonymous codons ending in A or T was much higher than other synonymous codons, and they accounted for 89.7% (3,330) of the total codons. The third position was A or T for all codons with RSCU values greater than 1. The six most frequently used codons were also composed of A and T, namely TTT, TTA, ATT, ATA, TAT and AAT, accounting for 40.7% of the total number of codons.

Fig 2. Relative synonymous codon usage (RSCU) in *Episymploce splendens* mito-genome. A total of 3,711 codons for *E. splendens* mito-genome were analyzed, excluding stop codons. Leu, Leu*, Ser, and Ser* indicate trnL1 (CUN), trnL2 (UUR), trnS1 (AGN), and trnS2 (UCN), respectively.

Protein-coding genes

All PCGs of *E. splendens* used ATN as the start codon, except for *COX1*. The *COX1* gene in the *E. splendens* mito-genome used TTG as the starting codon, in agreement with other known cockroaches [5]. The stop codon was most commonly TAA in the *E. splendens* mito-genome, followed by TAG (*NAD1*). Four protein coding genes: *NAD4*, *NAD6*, *ATP6*, and *NAD3*, used incomplete TA as the stop codon.

A+T rich region

The non-coding region of *E. splendens* mito-genome was located between srRNA and tRNA-Ile, and it was 290 bp long with 64.8% A+T content. Two 120 bp long repeated units separated by a 7 bp interval and four Poly-A structures were found in the A+T rich region of the *E. splendens* mito-genome (Fig 3).

Fig 3. The A +T rich region sequence of *Episymploce splendens* mito-genome. The blue and gray areas represented two 120bp-long repeated segments, respectively. The red fonts represented the poly-A structures.

Numts and its divergence time

Numts are originated from mt-genome. They have a low rate of evolution, and the pattern of nucleotide substitution is similar to ancient mtDNA. So they can be called "fossil" markers. Numts can be used to solve some problems in phylogeny, such as Zischler et al used the Numt as phylogenetic outgroup to prove the origin of man [34].

There were two Numts found in *E. splendens*, namely Numt-1 (557 bp) and Numt-2 (975 bp). Comparisons with aligned mitochondrial sequence showed 87.23% homologies in Numt-1 and 76.63% in Numt-2 (Figs S3 and S4). Some characteristics in Numts such as the deletion mutation, base substitution and insertion mutation were also found in Numt-1 and Numt-2 [35-36].

The timescale for evolution of 25 species and Numt-1 diversification based on aligning sequences and calibrations based on three cockroach fossils is shown in Fig 4(a) while the timescale for Numt-2 is shown in Fig 4(b). The divergence of the lineages leading to *Blattella* and *Episymploce* was 22.60 Ma to 36.50 Ma (95% confidence interval [CI]) in Fig 4(a) while the estimated age of the split between them was 15.54 Ma to 34.27 Ma (95% confidence interval [CI]) in Fig 4(b). Results were similar although different aligning sequences were used to calculate the divergence time in this study (Fig 4). Numt-1 transferred from the mitochondrion to the nucleus between 14.15 Ma to 22.35 Ma (95% confidence interval [CI]), and Numt-2 were estimated to have diverged between 19.19 Ma to 24.06 Ma (95% confidence interval [CI]).

Fig 4(a). Phylogenetic chronogram of Numt-1 and blattodean species based on the sequence aligned with Numt-1, reconstructed using BEAST. The best-fit evolution model was determined by PartitionFinder. Scale bar estimates age in millions of years and blue bars represent 95% highest posterior density intervals for the node ages. *Papilio protenor* and *Biston panterinaria* were employed to root the tree as outgroups.

Fig 4(b). Phylogenetic chronogram of Numt-2 and blattodean species based on the sequence aligned with Numt-2, reconstructed using

BEAST. The best-fit evolution model was determined by PartitionFinder. Scale bar estimates age in millions of years and blue bars represent 95% highest posterior density intervals for the node ages. *Papilio protenor* and *Biston panterinaria* were employed to root the tree as outgroups.

Discussion

Intergenic spacer

Mito-genomes typically exhibited compact arrangements, such as small gene spacing, gene overlap, or incomplete stop codons. However, the *E. splendens* mito-genome had a long intergenic spacer region (93 bp) located between tRNA-Val and srRNA, which is the longest intergenic spacer region of the reported cockroach mito-genomes. The long intergenic spacers in mito-genomes have been reported in some Hymenopteran [37-38], Hemipteran [39-40], Dictyopteran [41] and Coleopteran insects [6] [42-43]. There are two commonly posited evolutionary mechanisms for the origin of mitochondrial intergenic spacers, the duplication/random loss model and slipped-strand mispairing [6] [41]. We could not find a homologous sequence with both ends in this intergenic spacer, thus its formation is difficult to explain by slipped-strand mispairing [6] [44]. A similar long intergenic spacer was reported in a blattarian insect mito-genome, *Blaptica dubia* (71-bp between tRNA-Gln and tRNA-Met) [41], and the duplication/random loss

model was used to explain the formation of this intergenic spacer [6] [41][45-46]. We suggested that this intergenic region may be derived from the replication of the 3' end of the srRNA when the DNA double helix unraveled, followed by random loss of partial duplicated gene, and then the residues formed the 93-bp remaining intergenic spacer in *E. splendens* (Fig 5).

Fig 5. Putative formation mechanism of the intergenic spacer in *Episymploce splendens* mito-genome. The Randomly copied fragment was marked with *

Animal mito-genomes were generally considered to be economic and optimized for rapid replication and transcription [47]. Therefore, mitochondrial evolution had traditionally been regarded as favoring genome size reduction [3] [48-49], possibly by eliminating intergenic spacers [50]. Eliminating nonfunctional intergenic spacers in mitochondrial evolution was important in the highly reduced and efficient mito-genomes [43]. But with the discovery of more large intergenic spacers in mito-genomes, as several containing additional origin of replication (*Apis mellifera*, *Triatoma dimidiata*, *Bombus ignitus*) [33] [39] [51], tandem repeat units (*Triatoma dimidiata*, *Pyrocoelia rufa*) [39] [42], or even open reading frames retained (*Triatoma dimidiata*) [39], whether

these long spacer regions were functional was controversial [39] [52]. We analyzed the large intergenic spacers in the mito-genomes of several insects, comparing the length and similarity of these large intergenic spacer sequences with their root sequences (S4 Table) [6] [40-41]. We found these spacer sequences were usually shorter than their root sequences and the greater the length difference between these spacer and their root sequences, the lower the similarity between them. It implied that these mito-genomes lost partial duplicated nucleotides in mitochondrial evolution. In addition, large intergenic spacers found in several genetically close species were not homologous, implying their origin occurred independently after species differentiation [6]. This suggests that these long intergenic spacers did not confer an evolutionary advantage, and they were gradually deleted during mitochondrial evolution. Consequently, we consider that the mito-genome may continue evolving towards compact arrangement and the long intergenic region may gradually decrease or even disappear during mito-genome evolution. However, these discoveries of large intergenic spacers in insect mito-genomes contribute to species identification, and also provide valuable information for the study of the evolution of insect mitochondrial genomes.

tRNAs deletion

Mitochondrial gene content, arrangement and composition were highly conserved, and mito-genomes typically contained 37 coding genes [3] [53]. However, some exceptions, such as gene duplications, deletions or rearrangements, were found in some species [54-56]. Although tRNA deletion is unusual, increasing cases have been reported, such as three amphibians species [54，57], three reptile species [58-59], one crustacean species [60], one Hemiptera insect and one Coleoptera insect [61], four Psocoptera insects[10,62] and three Mantode*a* insects [9]. In this study, two tRNAs, tRNA-Gln and tRNA-Met, were absent in *E. splendens* mito-genome. The tRNA deletions was only found in the *E. splendens* mito-genome (this study) in all Blattaria mito-genomes reported and the tRNA deletion events reported in previous research also scattered in different clade branches, therefore, we consider tRNA deletions appear to be separate events occasionally occurring in some species or evolutionary branches. The deletion of functional genes was obviously disadvantageous for species, and the mechanism of deletion is still unclear.

The organism may have a functional replacement to cope with the loss of tRNAs. Two mechanisms were proposed for this functional compensation. The first mechanism where tRNAs from the cytosol are imported into mitochondria has been confirmed, as aminoacyl-tRNA synthetases being imported from the cytosol into mitochondria [63], and

functional tRNA-Lys encoded in the nuclear genome being imported into marsupial mitochondria [64]. The second compensation mechanism for the missing tRNAs is 'superwobble', where a tRNA with an unmodified U in the wobble position reads all four nucleotides in the third codon position [65]. In our study, the loss of tRNA-Gln and tRNA-Met can be compensated with their first and second codons matching His and Ile, respectively, and the anticodon swing site U for His and Ile. We calculated the Relative Synonymous Codon Usage (RSCU) values of CAU (H) and AUU (I) in reported cockroach mito-genomes, and discovered that *E. splendens* mito-genome had the highest RSCU values (Table 4), indicating that *E. splendens* might be compensated through tRNA superwobble. Regardless, we cannot exclude tRNA import from the cytosol in *E. splendens* mitochondrion. Therefore, more studies are needed to confirm the compensation mechanism for the absence of tRNAs in the mito-genome of *E. splendens*.

Table 4. The RSCU values of CAU (H) and AUU (I) in known cockroaches

Cockroaches	CAU (H)	AUU (I)	GenBank Accession number
Episymploce splendens	1.69	1.86	OK094023
Blattella germanica	1.54	1.83	NC_012901.1
Blattella bisignata	1.57	1.63	NC_018549.1
Panchlora nivea	1.57	1.77	NC_030002.1
Shelfordella lateralis	1.63	1.63	NC_030003.1
Nauphoeta cinerea	1.49	1.64	NC_035052.1
Cryptocercus meridianus	1.51	1.49	NC_037496.1
Periplaneta australasiae	1.44	1.7	NC_034841.1
Neostylopyga rhombifolia	1.45	1.66	NC_034842.1

Periplaneta brunnea	1.42	1.71	MG010455
Blaptica dubia	1.55	1.54	NC_029224.1
Periplaneta americana	1.36	1.71	NC_016956.1
Gromphadorhina portentosa	1.45	1.42	NC_030001.1
Periplaneta fuliginosa	1.43	1.73	NC_006076.1
Opisthoplatia orientalis	1.35	1.6	NC_029225.1
Cryptocercus relictus	1.21	1.57	NC_018132.1
Cryptocercus kyebangensis	1.33	1.6	NC_030191.1
Eupolyphaga sinensis	0.93	1.64	NC_014274.1

Conclusion

In this study, we sequenced and annotated the *E. splendens* mito-genome. Two tRNAs (tRNA-Gln, tRNA-Met) were lost and a long intergenic region between tRNA-Val and srRNA (93 bp, with a 64.6% similarity with its corresponding srRNA) was also found in *E. splendens* mito-genome. The duplication/random loss model may account for the origin of this long intergenic spacer. We also found two Numts, Numt-1 and Numt-2 transfered from mitochondrion to nucleus at about 14.15 Ma to 22.35 Ma, and 19.19 Ma to 24.06 Ma respectively.

Acknowledgments

We sincerely appreciate Natural History Museum of Sichuan University for the sample collection. Thanks Dr. Megan for language help.

Author Contributions

Conceptualization: XYZ JNM BSY.

Funding acquisition: XYZ.

Investigation: LY ZZH HMW JG QC CJZ.

Resources: LY BSY XYZ JG CJZ.

Writing – original draft: LY ZZH HMW JG QC.

Writing – review & editing: LY XYZ JNM BSY.

References

[1] Wilson AJ, Xu J. Mitochondrial inheritance: diverse patterns and mechanisms with an emphasis on fungi. Mycology. 2012; 3(2): 158-166. doi: 10.1080/21501203.2012.684361. PMID: 7219536.

[2] Ojala D, Montoya J, Attardi G. tRNA punctuation model of RNA processing in human mitochondria. Nature. 1981; 290(5806): 470-474. doi: 10.1038/290470a0. PMID: 7219536.

[3] Boore JL. Animal mitochondrial genomes. Nucleic Acids Res. 1999; 27(8): 1767-1780. doi: 10.1093/nar/27.8.1767. PMID: 10101183.

[4] Li WJ, Wang ZQ , Che YL，The Complete Mitogenome of the Wood-Feeding Cockroach Cryptocercus meridianus (Blattodea: Cryptocercidae) and Its Phylogenetic Relationship among Cockroach Families. Int. J. Mol. Sci. 2017; 18, 2397.doi: 10.3390/ijms18112397. PMID: 29137151. PMCID: PMC5713365

[5] Gong R, Guo X, Ma J, Song X, Shen Y, Geng F, et al. Complete mitochondrial genome of *Periplaneta brunnea* (Blattodea: Blattidae) and phylogenetic analyses within Blattodea. J Asia-Pac Entomol. 2018; 21(3): 885-895. doi: 10.1016/j.aspen.2018.05.006.

[6] Du C, Zhang L, Lu T, Ma J, Chen Z, Yue B, et al. Mitochondrial genomes of blister beetles (Coleoptera, Meloidae) and two large intergenic spacers in *Hycleus* genera. BMC Genomics. 2017; 18(1): 698. doi: 10.1186/s12864-017-4102-y. PMID: 28874137.

[7] Simon S, Hadrys H. A comparative analysis of complete mitochondrial genomes among Hexapoda. Molecular Phylogenetics and Evolution.2013; 69(2) :393–403. doi: 10.1016/j.ympev.2013.03.033. PMID: 23598069

[8] Chen ZT, Du YZ. Rearrangement of mitochondrial genome in insects. Journal of Environmental Entomology. 2016; 38 (4) : 843 — 851.(In Chinese)

[9] Shi Y, Li LY, Liu QP, Ali MY, Yuan ZL, Guy S et al. Complete mitochondrial genomes of four species of praying mantises (Dictyoptera, Mantidae) with ribosomal second structure, evolutionary and phylogenetic analyses.PLoS ONE. 2021;16(11): e0254914. doi: 10.1371/journal.pone.0254914. PMID: 34735444. PMCID: PMC8568281.

[10] Shi Y, Chu Q, Wei DD, Qiu YJ, Shang F, Dou W, Wang JJ. The mitochondrial genome of booklouse, Liposcelis sculptilis (Psocoptera: Liposcelididae) and the evolutionary timescale of *Liposcelis*. Scientific RepoRts.2016;6:30660. doi: 10.1038/srep30660. PMID: 27470659. PMCID: PMC4965752

[11] Shao RF, Zhu XQ, Barker SC, Herd K. Evolution of extensively fragmented mitochondrial genomes in the lice of humans. Genome Biol Evol. 2012; 4(11): 1088–1101.doi:10.1093/gbe/evs088. PMID: 23042553. PMCID: PMC3514963.

[12] Chen SC, Wei DD, Shao RF, Shi JX, Dou W, Wang JJ. Evolution of multipartite mitochondrial genomes in the booklice of the genus Liposcelis (Psocoptera). BMC Genomics. 2014; 15(1):861. doi: 10.1186/1471-2164-15-861. PMID: 25282613. PMCID: PMC4197233.

[13] Zhang LP, Ma Y, Yu DN, Storey KB, Zhang JY. The mitochondrial genomes of *Statilia maculata* and *S. nemoralis* (Mantidae: Mantinae) with different duplications of *trnR* genes. Int J Biol Macromol. 2019; 121: 839–845.https://doi.org/10.1016/j.ijbiomac.2018.10.038.PMID:30340009

[14] Ye F, Lan XE, Zhu WB, You P. Mitochondrial genomes of praying mantises (Dictyoptera, Mantodea): rearrangement, duplication, and reassignment of tRNA genes. Scientific RepoRts.2016;.6:25634. doi: 10.1038/srep25634. PMID: 27157299.PMCID: PMC4860592.

[15] Wang SS, Hou FX, Cao J, Peng C, Guo JL. The complete mitochondrial genome of the *Statilia maculate*. (Mantodea: Mantidae). Mitochondrial DNA Part B Resources.2016;1(1):860-861.doi: 10.1080/23802359.2016.1250134.PMID:33490423.PMCID:PMC7800993.

[16] Ma JN, Du C, Zhou C, Shen YM, Fan ZX, Yue BS, et al. Complete mitochondrial genomes of two blattid cockroaches, *Periplaneta australasiae* and *Neostylopyga rhombifolia*, and phylogenetic relationships within the Blattaria. PLoS One. 2017; 12(5): e0177162. doi: 10.1371/journal.pone.0177162. PMID: 28486518.

[17] Bourguignon T, Tang Q, Ho Simon Y.W., Juna F, Wang ZQ, Arab D A., Cameron S L., Walker J, Rentz D, Evans T A., Lo N. Transoceanic Dispersal and Plate Tectonics Shaped GlobalCockroach Distributions: Evidence from Mitochondrial Phylogenomics. Molecular biology and evolution. 2018; 35(4):970-983. doi: 10.1093/molbev/msy013. PMID: 29420807.

[18] Li TT, Liu DX, Qiu DY, Yue QY. Two new species of Episymploce Bey-Bienko, 1950 (Blattodea, Ectobiidae, Blattellinae) from China. Zookeys. 2020; 954:31-45. doi: 10.3897/zookeys.954.49738. PMID: 32821203; PMCID: PMC7406546.

[19] Wang Z, Che Y. Three new species of cockroach genus *Symploce* Hebard, 1916 (Blattodea, Ectobiidae, Blattellinae) with redescriptions of

two known species based on types from Mainland China. Zookeys. 2013; 337(337): 1-18. doi: 10.3897/zookeys.337.5770. PMID: 24146575.

[20] Xiao B, Chen AH, Zhang YY, Jiang GF, Hu CC, Zhu CD. Complete mitochondrial genomes of two cockroaches, *Blattella germanica* and *Periplaneta americana*, and the phylogenetic position of termites. Curr Genet. 2012; 58(2): 65-77. doi: 10.1007/s00294-012-0365-7. PMID: 22311390.

[21] Chen AH. Complete mitochondrial genome of the double-striped cockroach *Blattella bisignata* (Insecta: Blattaria: Blaberoidea). Mitochondrial DNA. 2013; 24(1): 14-6. doi: 10.3109/19401736.2012.710228. PMID: 22897805.

[22] Lopez JV, Yuhki N, Masuda R, Modi W, O'Brien SJ. Numt, a recent transfer and tandem amplification of mitochondrial DNA to the nuclear genome of the domestic cat. J Mol Evol. 1994; 39(2): 174-90. doi: 10.1007/BF00163806. PMID: 7932781.

[23] Arctander P. Comparison of a mitochondrial gene and a corresponding nuclear pseudogene. Proc Biol Sci. 1995; 262(1363): 13-9. doi: 10.1098/rspb.1995.0170. PMID: 7479989.

[24] Laslett D, Canbäck B. ARWEN: a program to detect tRNA genes in metazoan mitochondrial nucleotide sequences. Bioinformatics. 2008; 24(2): 172-5. doi: 10.1093/bioinformatics/btm573. PMID: 18033792.

[25] Tamura K, Peterson D, Peterson N, Stecher G, Nei M, Kumar S. MEGA5: molecular evolutionary genetics analysis using maximum likelihood, evolutionary distance, and maximum parsimony methods. Mol Biol Evol. 2011; 28(10): 2731-2739. doi: 10.1093/molbev/msr121. PMID: 21546353; PMCID: PMC3203626.

[26] Perna NT, Kocher TD. Patterns of nucleotide composition at fourfold degenerate sites of animal mitochondrial genomes. J Mol Evol. 1995; 41(3): 353-358. PMID: 7563121.

[27] Drummond AJ, Rambaut A. BEAST: Bayesian evolutionary analysis by sampling trees. BMC Evol Biol. 2007; 7: 214. doi: 10.1186/1471-2148-7-214

[28] Gernhard T. The conditioned reconstructed process. J Theor Biol. 2008; 253(4): 769-778. doi: 10.1016/j.jtbi.2008.04.005. PMID: 18538793.

[29] Wolfe JM, Daley AC, Legg DA, Edgecombe GD. Fossil calibrations for the arthropod Tree of Life. Earth-Science Reviews, 2016; 160:43-110.

[30] Evangelista DA, Wipfler B, Bethoux O, Donath A, Fujita M, Kohli MK, et al. An integrative phylogenomic approach illuminates the evolutionary history of cockroaches and termites (Blattodea). Proceedings of the Royal Society. Biological sciences, 2019, 286(1895). DOI:10.1098/rspb.2018.2076.

[31] Vrsˇansky P. Origin and the early evolution of mantises. AMBA Projekty. 2002; 6(1): 1-16.

[32] Dotson EM, Beard CB. Sequence and organization of the mitochondrial genome of the Chagas disease vector, *Triatoma dimidiata*. Insect Mol Biol. 2001; 10(3): 205-215. PMID: 11437912.

[33] Crozier RH, Crozier YC. The mitochondrial genome of the honeybee *Apis mellifera*: complete sequence and genome organization. Genetics. 1993; 133(1): 97-117. PMID: 8417993; PMCID: PMC1205303.

[34] Zischler H, Geisert H, von Haeseler A, Pääbo S. A nuclear 'fossil' of the mitochondrial D-loop and the origin of modern humans. Nature.1995; 30;378(6556):489-92.doi: 10.1038/378489a0.PMID: 7477404

[35] Tourmen Y, Baris O, Dessen P, Jacques C, Malthièry Y, Reynier P. Structure and chromosomal distribution of human mitochondrial pseudogenes. Genomics. 2002; 80(1): 71-7. doi: 10.1006/geno.2002.6798. PMID: 12079285.

[36] Hazkani-Covo E, Sorek R, Graur D. Evolutionary dynamics of large numts in the human genome: rarity of independent insertions and abundance of post-insertion duplications. J Mol Evol. 2003; 56(2): 169-74. doi: 10.1007/s00239-002-2390-5. PMID: 12574863.

[37] Wei SJ, Tang P, Zheng LH, Shi M, Chen XX. The complete mitochondrial genome of *Evania appendigaster* (Hymenoptera: Evaniidae) has low A+T content and a long intergenic spacer between

atp8 and atp6. Mol Biol Rep. 2010; 37(4): 1931-42. doi:10.1007/s11033-009-9640-1. PMID:19655273.

[38] Rodovalho CM, Lyra ML, Ferro M, Jr MB. The mitochondrial genome of the leaf-cutter ant *Atta laevigata*: a mitogenome with a large number of intergenic spacers. PLoS One. 2014; 9(5): e97117. doi: 10.1371/journal.pone.0097117. PMID: 24828084.

[39] Dotson EM, Beard CB. Sequence and organization of the mitochondrial genome of the Chagas disease vector, *Triatoma dimidiata*. Insect Mol Biol. 2001; 10(3): 205-215. PMID: 11437912.

[40] Hua J, Li M, Dong P, Cui Y, Xie Q, Bu W. Comparative and phylogenomic studies on the mitochondrial genomes of Pentatomomorpha (Insecta: Hemiptera: Heteroptera). BMC Genomics. 2008; 9(1): 1-15. doi: 10.1186/1471-2164-9-610. PMID: 19091056; PMCID: PMC2651891.

[41] Cheng XF, Zhang LP, Yu DN, Storey KB, Zhang JY. The complete mitochondrial genomes of four cockroaches (Insecta: Blattodea) and phylogenetic analyses within cockroaches. Gene. 2016; 586(1): 115-122. doi: 10.1016/j.gene.2016.03.057. PMID: 27045773.

[42] Bae JS, Kim I, Sohn HD, Jin BR. The mitochondrial genome of the firefly, *Pyrocoelia rufa*: complete DNA sequence, genome organization, and phylogenetic analysis with other insects. Mol Phylogenet Evol. 2004; 32(3): 978-85. doi: 10.1016/j.ympev.2004.03.009. PMID: 15288070.

[43] Sheffield NC, Song H, Cameron SL, Whiting MF. A comparative analysis of mitochondrial genomes in Coleoptera (Arthropoda: Insecta) and genome descriptions of six new beetles. Mol Biol Evol. 2008; 25(11): 2499-2509. doi: 10.1093/molbev/msn198. PMID: 18779259.

[44] Stanton DJ, Daehler LL, Moritz CC, Brown WM. Sequences with the potential stem-and-Ioop structures are associated with coding-region duplications in animal mitochondrial DNA. Genetics. 1994; 137(1): 233-241. doi: 10.1101/gad.8.9.1131. PMID: 8056313.

[45] Moritz C, Dowling TE, Brown WM. Evolution of Animal Mitochondrial DNA: Relevance for Population Biology and Systematics. Annu Rev Ecol Evol S. 1987; 18: 269-292. doi: 10.1146/annurev.ecolsys.18.1.269

[46] Boore JL. Comparative Genomics: The duplication/random loss model for gene rearrangement exemplified by mitochondrial genomes of Deuterostome animals. 1st ed. Dordrecht: Springer; 2000.

[47] Sayadi A, Immonen E, Tellgren-Roth C, Arnqvist G. The Evolution of Dark Matter in the Mitogenome of Seed Beetles. Genome Biol Evol. 2017; 9(10): 2697-2706. doi: 10.1093/gbe/evx205. PMID: 29048527; PMCID: PMC5737749.

[48] Macey JR, Larson A, Ananjeva NB, Fang Z, Papenfuss TJ. Two novel gene orders and the role of light-strand replication in rearrangement of the vertebrate mitochondrial genome. Mol Biol Evol.

1997; 14(1): 91-104. doi: 10.1093/oxfordjournals.molbev.a025706. PMID: 9000757.

[49] McKnight ML, Shaffer HB. Large, rapidly evolving intergenic spacers in the mitochondrial DNA of the salamander family Ambystomatidae (Amphibia: Caudata). Mol Biol Evol. 1997; 14(11): 1167-76. doi: 10.1093/oxfordjournals.molbev.a025726. PMID: 9364774.

[50] Burger G, Gray MW, Lang BF. Mitochondrial genomes: anything goes. Trends Genet. 2003; 19(12): 709-16. doi: 10.1016/j.tig.2003.10.012. PMID: 14642752.

[51] Cha SY, Yoon HJ, Lee EM, Yoon MH, Hwang JS, Jin BR, et al. The complete nucleotide sequence and gene organization of the mitochondrial genome of the bumblebee, *Bombus ignitus* (Hymenoptera: Apidae). Gene. 2007; 392(1-2): 206-20. doi: 10.1016/j.gene.2006.12.031. PMID: 17321076.

[52] Jørgensen TE, Bakke I, Ursvik A, Andreassen M, Moum T, Johansen SD. An evolutionary preserved intergenic spacer in gadiform mitogenomes generates a long noncoding RNA. BMC Evol Biol. 2014; 14: 182. doi: 10.1186/s12862-014-0182-3. PMID: 25145347; PMCID: PMC4236577.

[53] Gemmell NJ, Janke A, Western PS, Watson JM, Pääbo S, Graves JA. Cloning and characterization of the platypus mitochondrial genome. J

Mol Evol. 1994; 39(2): 200-5. doi: 10.1007/BF00163808. PMID: 7932783.

[54] Zhang JF, Nie LW, Wang Y, Hu LL. The complete mitochondrial genome of the large-headed frog, *Limnonectes bannaensis* (Amphibia: Anura), and a novel gene organization in the vertebrate mtDNA. Gene. 2009; 442(1-2): 119-27. doi: 10.1016/j.gene.2009.04.018. Epub 2009 May 3. PMID: 19397958.

[55] Hlaing T, Tun-Lin W, Somboon P, Socheat D, Setha T, Min S, Chang MS, Walton C. Mitochondrial pseudogenes in the nuclear genome of *Aedes aegypti* mosquitoes: implications for past and future population genetic studies. BMC Genet. 2009; 10: 11. doi: 10.1186/1471-2156-10-11. PMID: 19267896; PMCID: PMC2660364.

[56] von Nickisch-Rosenegk M, Brown WM, Boore JL. Complete sequence of the mitochondrial genome of the tapeworm *Hymenolepis diminuta*: gene arrangements indicate that Platyhelminths are Eutrochozoans. Mol Biol Evol. 2001; 18(5): 721-30. doi: 10.1093/oxfordjournals.molbev.a003854. PMID: 11319256.

[57] San MD, Gower DJ, Oommen OV, Wilkinson M, Zardoya R. Phylogeny of caecilian amphibians (Gymnophiona) based on complete mitochondrial genomes and nuclear RAG1. Mol Phylogenet. 2004; 33(2): 413-27. doi:10.1016/j.ympev.2004.05.014

[58] Peng QL, Nie LW, Pu YG. Complete mitochondrial genome of Chinese big-headed turtle, *Platysternon megacephalum*, with a novel gene organization in vertebrate mtDNA. Gene. 2006; 380(1): 14-20. doi: 10.1016/j.gene.2006.04.001. PMID: 16842936.

[59] Kumazawa Y, Miura S, Yamada C, Hashiguchi Y. Gene rearrangements in gekkonid mitochondrial genomes with shuffling, loss, and reassignment of tRNA genes. BMC Genomics. 2014; 15: 1–13. doi: 10.1186/1471-2164-15-930. PMID: 25344428.PMCID: PMC4223735

[60] Kilpert F, Podsiadlowski L. The complete mitochondrial genome of the common sea slater, Ligia oceanica (Crustacea, Isopoda) bears a novel gene order and unusual control region features. BMC Genomics. 2006; 7: 241. doi: 10.1186/1471-2164-7-241. PMID: 16987408.PMCID: PMC1590035.

[61] Dai Y, Li L, Jiang P, Song F, Ye Z, Yuan X, et al. Sequence and organization of the mitochondrial genome of an urostylidid bug, *Urochela quadrinotata* Reuter (Hemiptera: Urostylididae). Entomotaxonomia, 2012; 34, 613-623.

[62] Wei DD, Shao R, Yuan ML, Dou W, Barker SC, Wang JJ. The multipartite mitochondrial genome of Liposcelis bostrychophila: insights into the evolution of mitochondrial genomes in bilateral animals. PLoS One. 2012; 7(3): e33973. doi: 10.1371/journal.pone.0033973. PMID: 22479490; PMCID: PMC3316519.

[63] Duchêne AM, Pujol C, Maréchal-Drouard L. Import of tRNAs and aminoacyl-tRNA synthetases into mitochondria. Curr Genet. 2009; 55(1): 1-18. doi: 10.1007/s00294-008-0223-9. PMID: 19083240.

[64] Dorner M, Altmann M, Paabo S, Morl M. Evidence for import of a lysyl-tRNA into marsupial mitochondria. Mol Biol Cell. 2001; 12(9): 2688-2698. doi: 10.1091/mbc.12.9.2688

[65] Rogalski M, Karcher D, Bock R. Superwobbling facilitates translation with reduced tRNA sets. Nat Struct Mol Biol. 2008; 15(2): 192-8. doi: 10.1038/nsmb.1370. PMID: 18193063.

Supporting Information

S1 Table. GenBank accession numbers for divergence date analyses.

S2 Table. Nucleotide composition in different regions of *Episymploce splendens*.

S3 Table. Relative synonymous codon usage (RSCU) for PCGs of *Episymploce splendens*.

S4 Table. The length and similarity of these large intergenic spacers with their original genes in some insects. The similarity was calculated by DNAMAN.

Fig. S1 Putative secondary structures of the 20 tRNA genes identified in *Episymploce splendens*.

Fig. S2 Aligement result of the long intergenic spacer and srRNA of *Episymploce splendens* by MEGA.

Fig. S3 Similarity comparison results of Numt-1 and mito-genome of *Episymploce splendens* by DNAMAN.

Fig. S4 Similarity comparison results of Numt-2 and mito-genome of *Episymploce splendens* by DNAMAN.

Fig 1

Click here to access/download;Figure;Fig 1..tiff

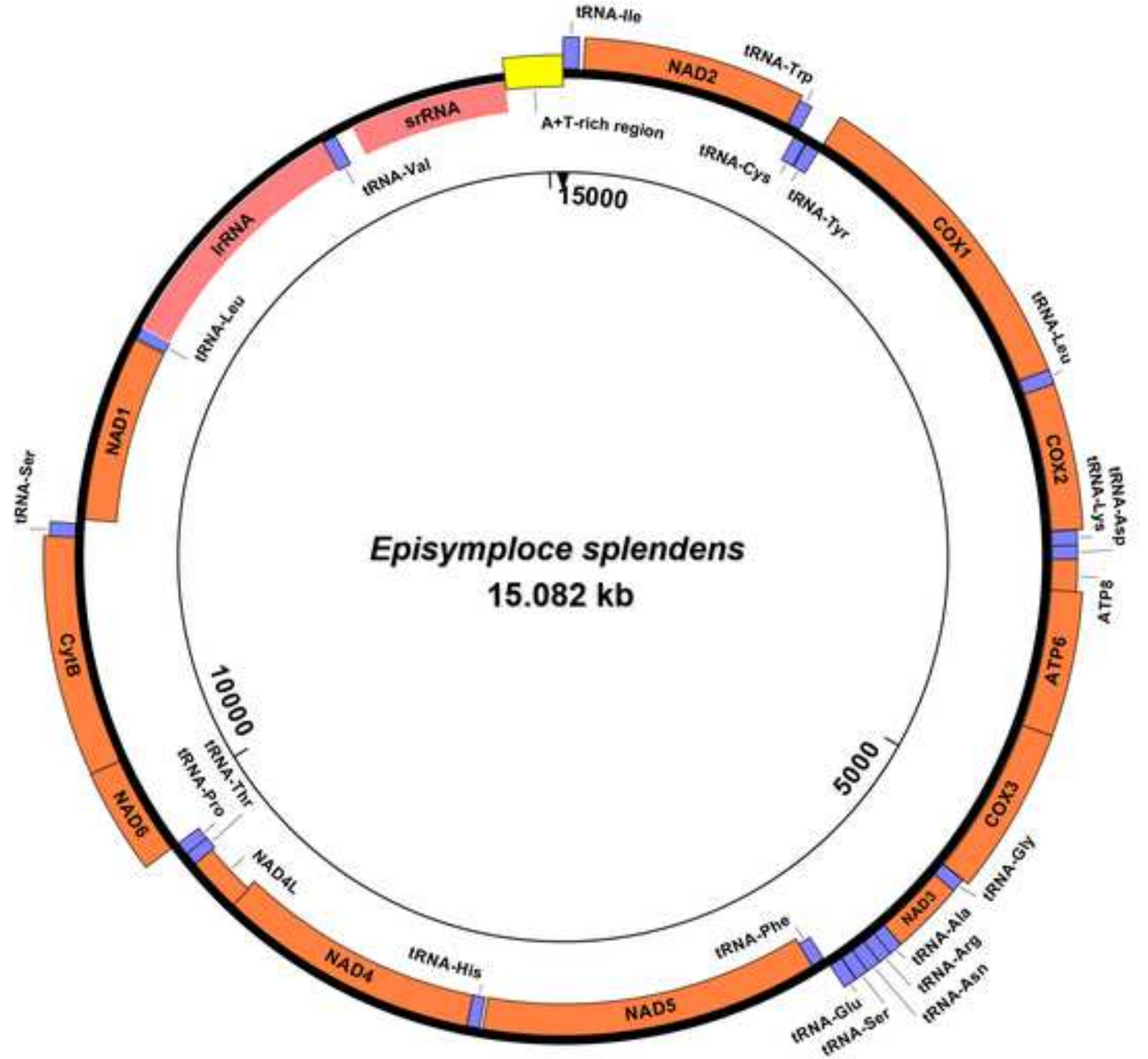

Fig 2

Click here to access/download;Figure;Fig 2..tif

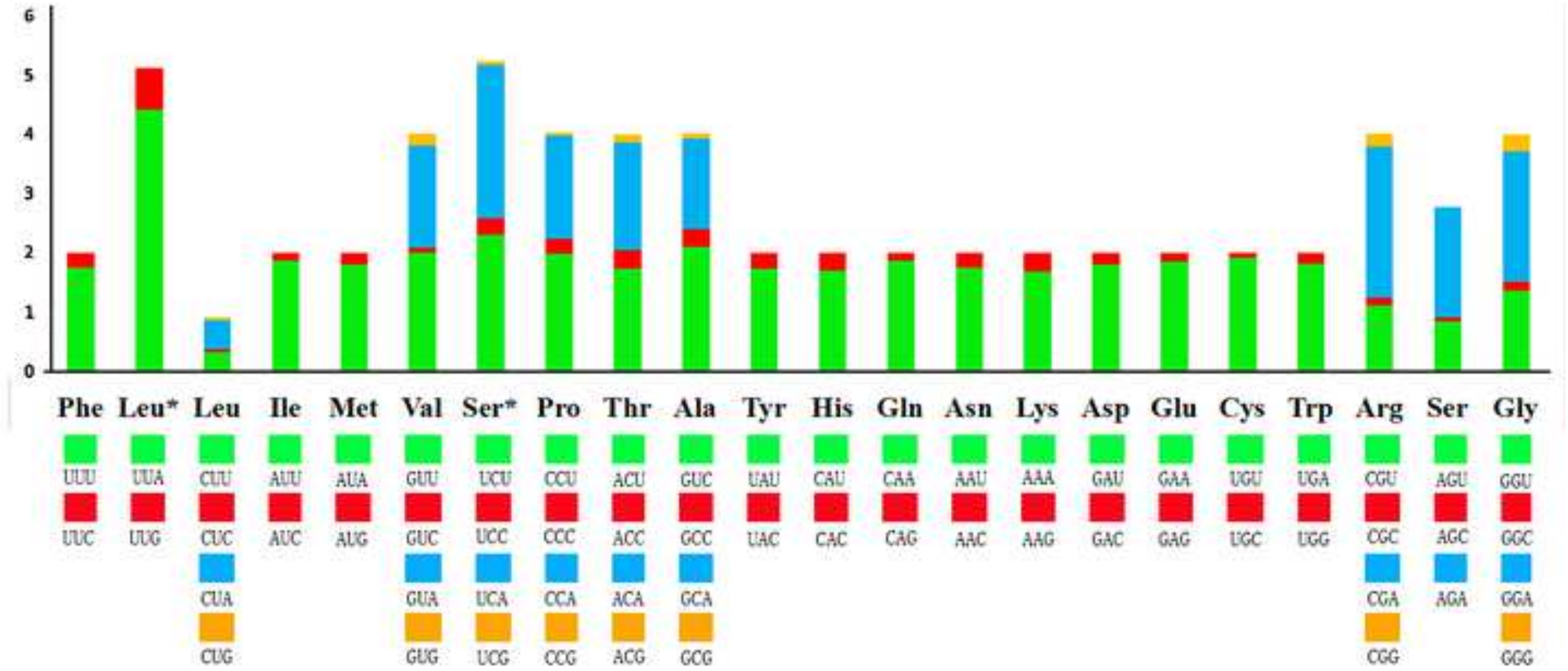

Click here to access/download;Figure;Fig 3..tif

TACCTAAAAATAAAGAGTGCCCCTGTCCCCCACTA
AACTATGATTTTACCTTACCTAAAAATAAAATAAG
TACAACTTCCTGCCCTTGTCCAATTTTCAACCTTTTC
AACCTTTTCAACCTTTTCAACCTAAATAAAAAATA
CCGTCCCTTGTCCCCCACTGAACTATGATTTTACCT
TACCTAAAAATAAAATAAGTACAACTTCCTGCCCT
TGTCCAATTTTCAACCTTTTCAACCTTTTCAACCTTT
TCAACCTAAATAAAAAATACCGTCCCTTGTCCAAT
CACCA

Fig 4(a)

Click here to access/download;Figure;Fig 4(a).tif

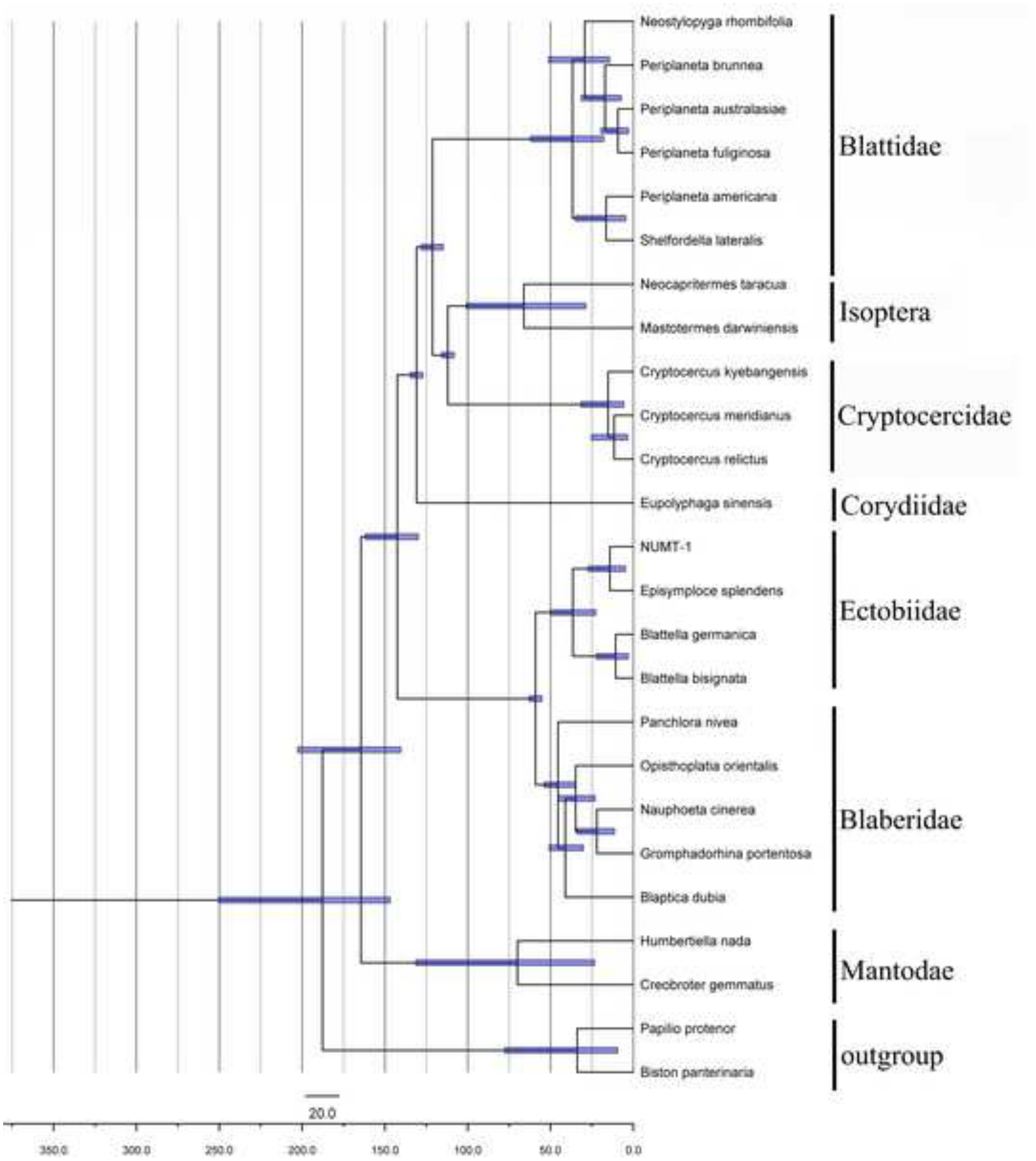

Fig 4(b)

Click here to access/download;Figure;Fig 4(b).tif

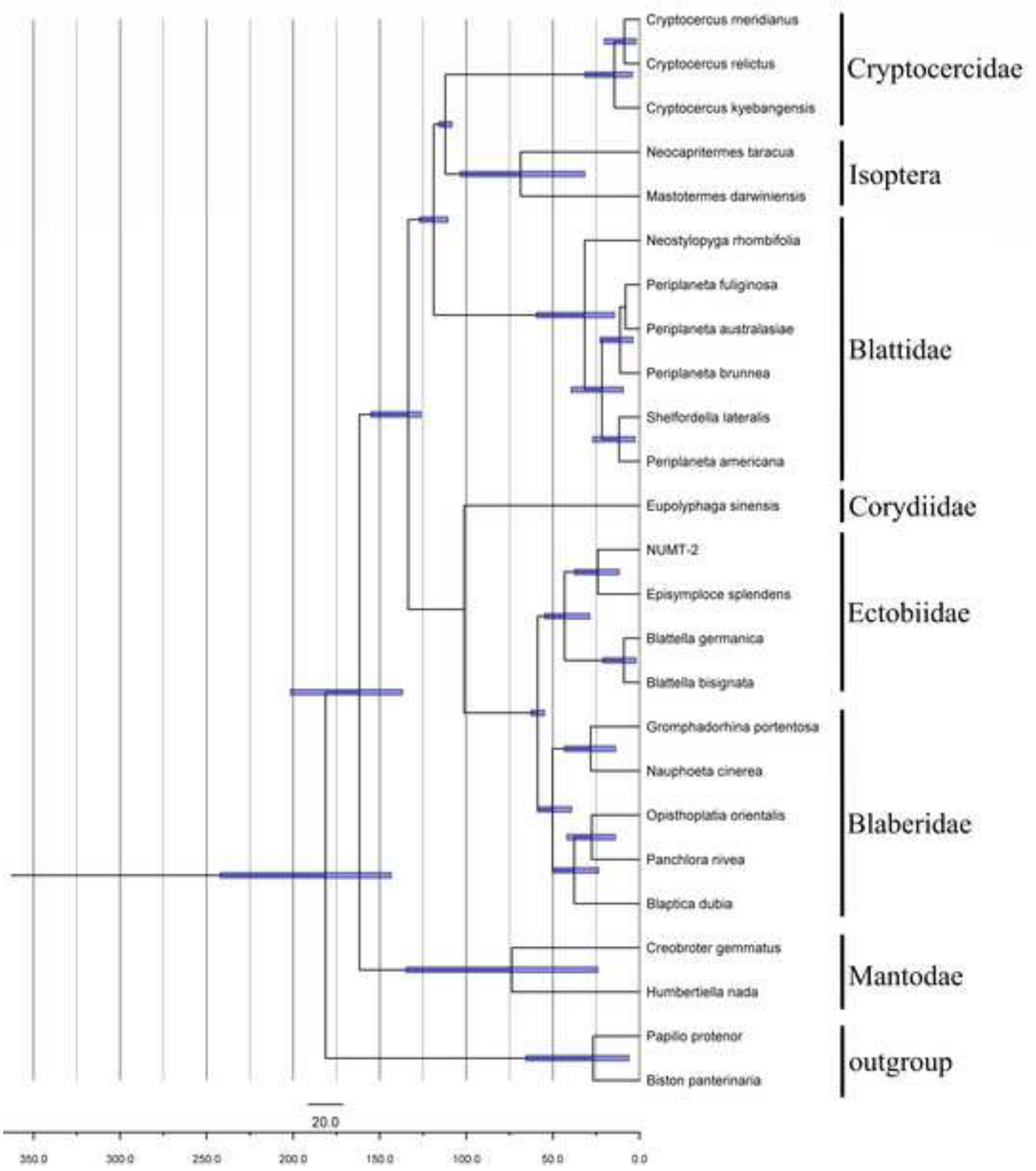

Fig 5

Click here to access/download;Figure;Fig 5..tif

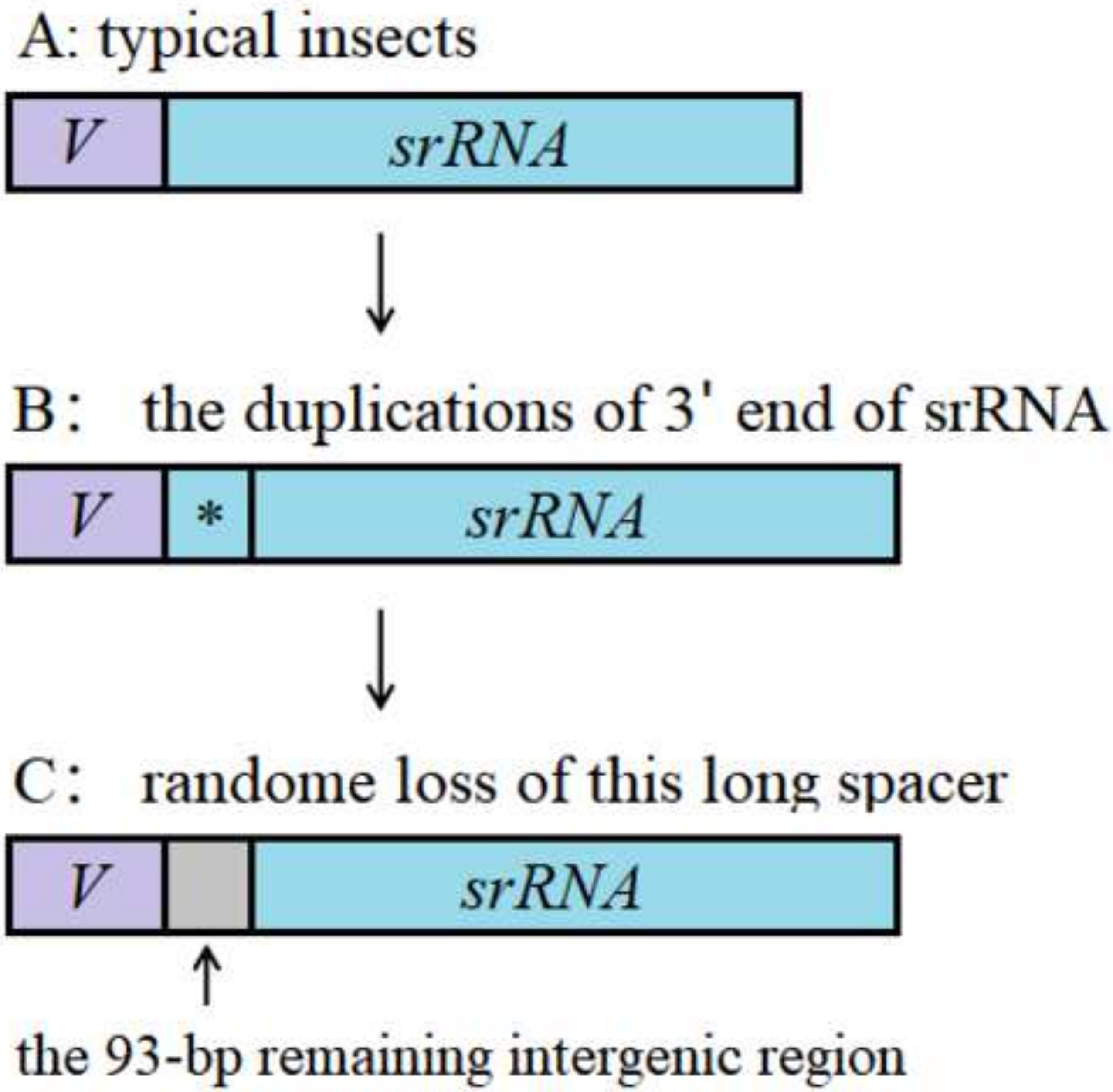

Click here to access/download
Supporting Information - Compressed/ZIP File Archive
supporting information.rar

Complete mitochondrial genome of *Episymploce splendens* (Blattodea: Ectobiidae): a large intergenic spacer and lacking of two tRNA genes

Lin Yan[1], Zhenzhen Hou[2], Jinnan Ma[1], Hongmei Wang[1], Jie Gao[3], Chenjuan Zeng[3], Qin Chen[1], Bisong Yue[1], Xiuyue Zhang[1,2]*

1Key Laboratory of Bio-resources and Eco-environment, Ministry of Education, College of Life Sciences, Sichuan University, No.24 South Section 1, Yihuan Road, Chengdu, China, 610065.

2Sichuan Key Laboratory of Conservation Biology on Endangered Wildlife, College of Life Sciences, Sichuan University, No.24 South Section 1, Yihuan Road, Chengdu, China, 610065.

3Sichuan Key Laboratory of Medicinal Periplaneta americana, Xichang, Sichuan Province 615000, China.

[*]E-mail: zhangxiuyue@scu.edu.cn

Abstract

The complete mitochondrial genome of *Episymploce splendens*, 15,802 bp in length, was determined and annotated in this study. The mito-genome included 13 PCGs, 20 tRNAs and 2 rRNAs. Unlike most typical mito-genomes with conservative gene arrangement and exceptional economic organization, *E. splendens* mito-genome has two tRNAs (tRNA-Gln and tRNA-Met) absence and a long intergenic spacer sequence (93 bp) between tRNA-Val and srRNA, showing the diversified features of insect mito-genomes. This is the first report of the tRNAs deletion in BlattarianOrthopteroidea mito-genomes and we supported the duplication/random loss model as the origin mechanism of the long intergenic spacer. Two Numts, Numt-1 (557 bp) and Numt-2 (975 bp) transferred to the nucleus at about 14.15 Ma to 22.34 Ma, and 19.19 Ma to 24.06 Ma respectively, were found in *E. splendens*. They can be used as molecular fossils in insect phylogenetic relationship inference. Our study provided useful data for further studies on the evolution of insect mito-genome.

Introduction

The mitochondrial genome has the characteristics of maternal inheritance, rare genetic recombination, faster evolution rate and conservative gene arrangement [1]. The mito-genome commonly displays

exceptional economy of organization, with overlapping genes or small intergenic spacers [2]. A typical insect mito-genome, with a compact circular molecule, generally encodes a fixed set of 37 genes including 13 protein-coding genes (PCGs), 22 transfer RNA (tRNA) genes, two ribosomal RNA (rRNA) genes and a control region [3]. However, exceptions have been reported, such as ~~diversity of *COX1* gene initiation codons,~~ long gene intergenic spacer~~s~~[4-6], gene rearrangement~~s~~ and gene loss[7-8]. So far, mitochondrial gene rearrangement has been reported in about 17 orders of insects, and these genes include protein coding gene, rRNA gene and tRNA gene[8]. In addition tRNAs, the missing protein coding genes have also been reported in Mantodea, Phthiraptera, and Psocoptera[9-13]. The tRNAs are most various in no-typical insect mito-genomes, including tRNA translocation, tRNA loss, tRNA tandem duplication and tRNA conversion[4,9-10,14-15]. Phthiraptera and Psocoptera insect mito-genomes may be most multipartite and most fast in evolution, that they could fission into fragmented mito-genomes and have numerous pseudo-genes and diverse gene rearrangement[7-10]. ~~different control regions with great variation [4-7].~~ Thus, insect mitochondrial genomes are good models for researching mitochondrial genome evolution.

There are of 4,600 species of cockroaches [16] and 300 mitochondrial genomes of them have been sequenced and uploaded to the

NCBI database. The Blattarian mito-genomes seem to be in a conserved evolution[16-17]. The gene rearrangement was only reported in Cryptocercidae within Blattarian mito-genomes [4]. *Episymploce splendens* belongs to Ectobiidae and at present, no complete mitochondrial sequences of *Episymploce* have been recorded in the NCBI database. Previous studies have mainly focused on the morphological features of *Episymploce* rather than molecular data [~~8-9~~18-19]. In this study, we sequenced, annotated and described the complete mito-genome of *E. splendens*. Two tRNAs loss and a long intergenic region was found in *E. splendens* mito-genome and two pseudo-genes were also identified in the study. This study could deepen our understanding of mitochondrial genome of insects and contribute to the study of insect mito-genome evolution.

Materials and Methods

Sample and DNA extraction

The *Episymploce* species have typical characteristics of adult cockroaches such as two sets of wings, sclerotized tegmina and wider membranous hindwings [~~8~~18]. Besides, the pronotum of *E. splendens* is black and one-orange patch is on each side of the lateral border. The front and hind wings are fully developed and through the end of the abdomen. Supra-anal plate of male genitalia is asymmetrical.

The cockroaches used in this study were collected on Mount Emei,

Sichuan Province, China. The fresh material was placed in absolute ethanol and stored at -20 ℃. Total genomic DNA was extracted from the muscle tissue (legs) using TIANamp Genomic DNA kit (TIANGEN, Beijing, China). The concentration and purity of total DNA were detected by spectrophotometer. Firstly, 2 μl DNA elution solution was taken to calibrate the instrument. Then, the spot sample hole was wiped clean and 2 μl DNA was taken to determine the concentration and purity of the test sample. In addition, DNA was detected by agarose gel electrophoresis, and 1% agarose gel electrophoresis judged whether DNA was successfully extracted or not. Finally, DNA was stored at -20 ℃.

PCR amplification and sequencing

Primers were designed based on the conserved sequences of *Blattella germanica* and *Blattella bisignata*, and then specific primers were designed based on the amplified and sequenced sequences at both ends [~~10~~20-~~11~~21]. The software Primer Premier 5.0 was used to designed primers and the primer details are listed in Table 1. Primers Es3, Es9 and Es10 were obtained from Xiao *et al* [~~10~~20]. PCR was conducted using 2×Taq PCR Mix (Innovagene, Chengdu, China) and performed on a PTC-100 thermal cycler (BioRad, Hercules, CA) with the following cycling conditions: an initial denaturation for 5 min at 94℃, followed by 35 cycles of denaturation for 30s at 94℃, annealing for 30s at 50-62℃ (depending on primer combinations), elongation for 1-4 min (depending

on putative length of the fragments) at 72℃, and a final extension step of 72℃ for 10 min. PCR products were estimated by 1.0% agarose gel electrophoresis and sent to Tsingke Biotechnology Company (Chengdu, China) for sequencing. All fragments were bidirectional sequences, and the unsuccessful sequenced fragments were redesigned with primers and sequenced again to complete the sequence.

Table 1. Primers for the PCR amplifications of *Episymploce splendens* (Es) mito-genome and Numts.

Primer name	Upstream primers sequences(5'-3')	Downstream primers sequences(5'-3')	Anneal temperature (℃)	Extension time (Second)
Es1	140-CCTCTCTTATCGCAATGTCCA	1609-CGTGGGAAAGCTATATCAGGA	58	90
Es2	1367-GGTCAACAAATCATAAAGATATTGG	2074-TAAACTTCAGGGTGACCAAAAAATCA	55	45
Es3	1559-CAACATTTATTTTGATTCTTTGG	3649-GTTTAAGAGACCACCACTTG	50	90
Es4	3401-GAAGACTTTCACCAACCATC	4391-TAGTACACTCATCTACTCTGGTAAC	52	60
Es5	4031-TGTAACAGCCCATGCTT	5797-AATCGCAATGATGGTAGG	51	110
Es6	4945-AGAAGACTTTCACCAACCAT	6997-GGATTCTCAAGATATTCGTT	51	120
Es7	6378-TCAACCGTTATCGAAAGACT	7328-CTCCTACTCCTGTATCTGCTT	52	60
Es8	7010-AACGAATATCCTGAGAATCC	8415-CACGGATTATGTTCTTCAGG	52	90
Es9	8290-GAAGGGGGTGCTGCTATATTAC	11151-ATTACTCCTCCTAATTTATTAGGAAT	62	180
Es10	10491-CAATGAGTATGAGGAGGATTTGCTGT	14755-TGTGCCAGCAGTCGCGGTTATACA	59.5	240
Es11	13822-CAGATTATATTGATTCGCACAAC	303-ATAGAACTGATGAAGCTAAGGC	55	75
Numt-1-1	GATTACGCTGTTATCCCTAAG	GGTGTAACTAGAATGATACAGGT	51	65
Numt-1-2	CGGTTTGAACTCAGATCATGTAAG	GAAGGTGTAACTAGAATGATACAGGT	57	78
Numt-2-1	TGCTACCTTTGCACGGTC	AGGTGAGATAAGTCGTAACATAGT	53	54
Numt-2-2	TAAACTCTATAGGGTCTTCTCG	CTAGAATGATACAGGTTAGGCT	53	70

The sequences which originated from the invasion of nuclear DNA by mtDNA were named Numts [~~12~~22]. Primers that amplified mitochondrial genes may occasionally amplify Numts [~~13~~23]. In this study, agarose gel electrophoresis of the amplification product (the primer Numt-1-1 and Numt-2-1, Table 1) appeared as two bands. We then redesigned primers (Numt-1-2 and Numt-2-2, Table 1) to amplify these regions and obtained the same result. These non-targeted bands were purified and sequenced, and obtained sequences belonging to mitochondria via the analysis of NCBI blast. Due to differences in mutation rates or freedom from selection pressure, they had some degree of base differences compared to corresponding mito-genome sequences.

Sequence analysis and annotation

DNA SeqMan program, which is included in the Lasergene software package (DNAStar Inc. Madison Wis), was used to assemble sequences to obtain the complete mitochondrial genome. PCGs and rRNAs were identified by comparing *E. splendens* to *B. germanica* and *B. bisignata* [~~10~~20-~~11~~21]. Most of the tRNA and their secondary structure inferences were conducted using the online server ARWEN (http://mbio-serv2.mbioekol.lu.se/ARWEN/) [~~14~~24]. The tRNA-Ile, tRNA-Phe and tRNA-Leu were not identified by ARWEN; they were manually recognized by referring to secondary structural models of other blattaria insects. The mitogenomic map was depicted with SeqBuilder

(http://www.dnastar.com). The A+T content of the nucleotide sequence and relative synonymous codon usage (RSCU) were calculated using MEGA 5.2 [~~15~~25]. The AT skewness was calculated according to the following formula: AT skew = [A-T]/[A+T], and the GC skewness was calculated according to the following formula: GC skew = [G-C]/[G+C] [16].

Divergence dating analysis

There were two Numts found in *E. splendens*, namely Numt-1 and Numt-2. Through sequence alignment by MEGA 5.2, Numt-1 corresponded to partial lrRNA, and Numt-2 was similar to partial lrRNA and its neighboring tRNA-Val of the *E. splendens* mito-genome. We performed divergence date analyses based on the aligned sequences of Blattodea, two mantises and two outgroups (S1 Table). The molecular clock was calibrated using three minimum age constraints based on cockroach fossils, as shown in Table 2. A relaxed molecular-clock model was used for this study with the program BEAST 1.6.1 [~~17~~27]. Rate variation was modeled among branches using uncorrelated lognormal relaxed clocks [~~17~~27]. A Yule speciation process was used for the tree prior and posterior distributions of parameters, including the tree, were estimated using MCMC sampling [~~18~~28]. Two independent runs (each with 4 chains) of 100 million generations were sampled every 5000 generations based on the GTR model. The tree topology was then

estimated using the combined sample from the last 50 million generations of each run.

Table 2. Fossils used for estimation of divergence time of Numts in the analysis.

species	Age (Ma)	Calibration Group	Reference
Valditermes brennae	130.8	*Cryptocercus* + Isoptera	[~~19~~29]
"Gyna" obesa	57.7	Blaberidae	[~~20~~30]
Cratomastotermes wolfschwenningeri	113	termites	[~~21~~31]

Results

Genome content and organization

The mitochondrial genome of *E. splendens* was 15,802 bp in length with typical circular molecules. It coded for 35 mitochondrial genes: 13 PCGs, 2 rRNAs (srRNA and lrRNA), 20 tRNAs and the A+T rich region (Fig 1, Table 3). Putative secondary structures of the 20 tRNAs were shown in Fig. S1. Similar to most species' mitochondria, the coding genes of ~~the~~ *E. splendens* mito-genome are compact, with several genes overlapping. There were five overlaps totaling 21 bp with the two longest overlaps being 7 bp between *ATP8* and *ATP6*, and 7 bp between *NAD4* and *NAD4L*. There were 15 gene spacers totaling 190 bp within the entire mitochondrial genome. The longest spacer was 93 bp between tRNA-Val and srRNA, followed by 24 bp between tRNA-Ile and *NAD2* and 22 bp between *NAD1* and tRNA-Leu and it had a similarity of 64.6% compared

to its adjacent and corresponding srRNA (Fig. S2). There were 16 areas with neither gene overlap nor intergenic spacer. Additionally, there were 20 tRNAs in the mitochondrial genome of *E. splendens*, lacking two tRNAs located between tRNA-Ile and *NAD2*, tRNA-Gln and tRNA-Met (Fig 1).

Fig 1. Circular gene map of ~~the mito-genomes of~~ *Episymploce splendens* mito-genome. Genes coded in the J-strand are inside of the circle. Gene coded in the N-strand are outside of the circle. *COX1*, *COX2* and *COX3* refer to the cytochrome C oxidase subunits; *CytB* refers to cytochrome B; ATPase6 and ATPase8 refer to ATP synthase subunits 6 and 8 genes; and *NAD1-NAD6* and *NAD4L* refer to the NADH dehydrogenase subunit 1-6 and 4Lgenes.

Table 3. Annotation of *Episymploce splendens* mito-genome~~(Es)~~.

Gene	Grand	Location	Anticodon	Start codon	Stop codon
tRNA-Ile	J	1..77	TAT		
NAD2	J	102..1148		ATG	TAA
tRNA-Trp	J	1149..1212	TCA		
tRNA-Cys	N	1208..1271	GCA		
tRNA-Tyr	N	1278..1347	GTA		
COX1	J	1350..2885		TTG	TAA
tRNA-Leu(UUR)	J	2887..2954	TAA		
COX2	J	2956..3642		ATG	TAA
tRNA-Lys	J	3645..3715	CTT		
tRNA-Asp	J	3717..3781	GTC		
ATPase8	J	3782..3940		ATT	TAA
ATPase6	J	3934..4613		ATG	TA-
COX3	J	4614..5402		ATG	TAA
tRNA-Gly	J	5405..5468	TCC		

NAD3	J	5469..5821		ATG	TA-
tRNA-Ala	J	5822..5886	TGC		
tRNA-Arg	J	5885..5950	TCG		
tRNA-Asn	J	5951..6020	GTT		
tRNA-Ser(AGN)	J	6021..6085	GCT		
tRNA-Glu	J	6088..6152	TTC		
tRNA-Phe	N	6162..6228	GAA		
NAD5	N	6229..7947		ATT	TAA
tRNA-His	N	7963..8027	GTG		
NAD4	N	8028..9367		ATG	TA-
NAD4L	N	9361..9642		ATG	TAA
tRNA-Thr	N	9643..9709	TGT		
tRNA-Pro	N	9709..9774	TGG		
NAD6	J	9776..10275		ATT	TA-
CytB	J	10276..11409		ATG	TAA
tRNA-Ser(UCN)	J	11409..11477	TGA		
NAD1	N	11500..12441		ATA	TAG
tRNA-Leu(CUN)	N	12451..12511	TAG		
lrRNA	N	12512..13822			
tRNA-Val	N	13823..13893	TAC		
srRNA	N	13987..14792			
A+T-rich region		14793..15082			

‘TA-’ refer to incomplete stop codons.

Nucleotide composition and codon usage

We calculated the nucleotide composition of the mtDNA in *E. splendens* using MEGA5.2, refer to S2 Table for detailed results. The content of A+T (74.6%) was higher than G+C (25. 4%). It corresponded well to the AT bias generally observed in insect mito-genomes, which ranges from 69.5 to 84.9% [22-23].

The high A+T content and nucleotide skewness of the mitochondrial genome were also reflected in the codon use of protein-coding genes. According to the relative synonymous codon usage (RSCU) value (S3

Table, Fig 2), the occurrence of synonymous codons ending in A or T was much higher than other synonymous codons, and they accounted for 89.7% (3,330) of the total codons. The third position was A or T for all codons with RSCU values greater than 1. The six most frequently used codons were also composed of A and T, namely TTT, TTA, ATT, ATA, TAT and AAT, accounting for 40.7% of the total number of codons.

Fig 2. Relative synonymous codon usage (RSCU) in ~~the mitochondrial genome of~~ *Episymploce splendens* mito-genome. A total of 3,711 codons for *E. splendens* mito-genome were analyzed, excluding stop codons. Leu, Leu*, Ser, and Ser* indicate trnL1 (CUN), trnL2 (UUR), trnS1 (AGN), and trnS2 (UCN), respectively.

Protein-coding genes

All PCGs of *E. splendens* used ATN as the start codon, except for *COX1*. The *COX1* gene in the *E. splendens* mito-genome used TTG as the starting codon, in agreement with other known cockroaches [~~23~~5]. The stop codon was most commonly TAA in the *E. splendens* mito-genome, followed by TAG (*NAD1*). Four protein coding genes: *NAD4*, *NAD6*, *ATP6*, and *NAD3*, used incomplete TA as the stop codon.

A+T rich region

The non-coding region of *E. splendens* mito-genome was located between srRNA and tRNA-Ile, and it was 290 bp long with 64.8% A+T content. Two 120 bp long repeated units separated by a 7 bp interval and four Poly-A structures were found in the A+T rich region of the *E. splendens* mito-genome (Fig 3).

Fig 3. The A +T rich region sequence of *Episymploce splendens* mito-genome**.** The blue and gray areas represented two 120bp-long repeated segments, respectively. The red fonts represented the poly-A structures.

Numts and its divergence time

Numts are originated from mt-genome. They have a low rate of evolution, and the pattern of nucleotide substitution is similar to ancient mtDNA. So they can be called "fossil" markers. Numts can be used to solve some problems in phylogeny, such as Zischler et al used the Numt as phylogenetic outgroup to prove the origin of man [24].

There were two Numts found in *E. splendens*, namely Numt-1 (557 bp) and Numt-2 (975 bp). Comparisons with aligned mitochondrial ~~aligned~~ sequence showed 87.23% homologies in Numt-1 and 76.63% in Numt-2 (Figs S3 and S4). Some characteristics in Numts such as the deletion mutation, base substitution and insertion mutation were also found in Numt-1 and Numt-2 [~~25~~35-~~26~~36].

The timescale for evolution of 25 species and Numt-1 diversification based on aligning sequences and calibrations based on three cockroach fossils is shown in Fig 4(a) while the timescale for Numt-2 is shown in Fig 4(b). The divergence of the lineages leading to *Blattella* and *Episymploce* was 22.60 Ma to 36.50 Ma (95% confidence interval [CI]) in Fig 4(a) while the estimated age of the split between them was 15.54 Ma to 34.27 Ma (95% confidence interval [CI]) in Fig 4(b). Results were similar although different aligning sequences were used to calculate the divergence time in this study (Fig 4). Numt-1 transferred from the mitochondrion to the nucleus between 14.15 Ma to 22.35 Ma (95% confidence interval [CI]), and Numt-2 were estimated to have diverged between 19.19 Ma to 24.06 Ma (95% confidence interval [CI]).

Fig 4(a). Phylogenetic chronogram of Numt-1 and blattodean species based on the sequence aligned with Numt-1, reconstructed using BEAST. The best-fit evolution model was determined by PartitionFinder. Scale bar estimates age in millions of years and blue bars represent 95% highest posterior density intervals for the node ages. *Papilio protenor* and *Biston panterinaria* were employed to root the tree as outgroups.

Fig 4(b). Phylogenetic chronogram of Numt-2 and blattodean species based on the sequence aligned with Numt-2, reconstructed using

BEAST. The best-fit evolution model was determined by PartitionFinder. Scale bar estimates age in millions of years and blue bars represent 95% highest posterior density intervals for the node ages. *Papilio protenor* and *Biston panterinaria* were employed to root the tree as outgroups.

Discussion

Intergenic spacer

Mito-genomes typically exhibited compact arrangements, such as small gene spacing, gene overlap, or incomplete stop codons. However, the *E. splendens* mito-genome had a long intergenic spacer region (93 bp) located between tRNA-Val and srRNA, which is the longest intergenic spacer region of the reported cockroach mito-genomes. The long intergenic spacers in mito-genomes have been reported in some Hymenopteran [~~27~~37-~~28~~38], Hemipteran [~~29~~39-~~30~~40], Dictyopteran [~~31~~41] and Coleopteran insects [~~5~~6] [~~32~~42-~~33~~43]. There are two commonly posited evolutionary mechanisms for the origin of mitochondrial intergenic spacers, the duplication/random loss model and slipped-strand mispairing [~~5~~6] [~~31~~41]. We could not find a homologous sequence with both ends in this intergenic spacer, thus its formation is difficult to explain by slipped-strand mispairing [~~5~~6] [~~34~~44]. A similar long intergenic spacer was reported in a blattarian insect mito-genome, *Blaptica dubia* (71-bp between tRNA-Gln and tRNA-Met) [~~31~~41], and

the duplication/random loss model could bewas used to explain the formation of this intergenic spacer [56] [3141][3545-3646]. We suggested that this intergenic region may be derived from the replication of the 3' end of the srRNA when the DNA double helix unraveled, followed by random loss of partial duplicated gene, and then the residues formed the 93-bp remaining intergenic spacer in *E. splendens* (Fig 5).

Fig 5. Putative formation mechanism of the intergenic spacer in *Episymplocemitochondrial genome of E. splendens* mitochondrial-genome. The Randomly copied fragment was marked with *

Animal mito-genomes were generally considered to be economic and optimized for rapid replication and transcription [3747]. Therefore, mitochondrial evolution had traditionally been regarded as favoring genome size reduction [3] [3848-3949], possibly by eliminating intergenic spacers [4050]. Eliminating nonfunctional intergenic spacers in mitochondrial evolution was important in the highly reduced and efficient mito-genomes [3343]. But with the discovery of more large intergenic spacers in mito-genomes, as several containing additional origin of replication (*Apis mellifera*, *Triatoma dimidiata*, *Bombus ignitus*) [2333] [2939] [4151], tandem repeat units (*Triatoma dimidiata*, *Pyrocoelia rufa*) [2939] [3242], or even open reading frames retained (*Triatoma dimidiata*)

[2939], whether these long spacer regions were functional was controversial [2939] [4252]. We analyzed the large intergenic spacers in the mito-genomes of several insects, comparing the length and similarity of these large intergenic spacer sequences with their root sequences (S4 Table) [56] [3040-3141]. We found these spacer sequences were usually shorter than their root sequences and the greater the length difference between these spacer and their root sequences, the lower the similarity between them. It implied that these mito-genomes lost partial duplicated nucleotides in mitochondrial evolution. In addition, large intergenic spacers found in several genetically close species were not homologous, implying their origin occurred independently after species differentiation [56]. This suggests that these long intergenic spacers did not confer an evolutionary advantage, and they were gradually deleted during mitochondrial evolution. Consequently, we consider that the mito-genome may continue evolving towards compact arrangement and the long intergenic region may gradually decrease or even disappear during mito-genome evolution. However, these discoveries of large intergenic spacers in insect mito-genomes contribute to species identification, and also provide valuable information for the study of the evolution of insect mitochondrial genomes.

tRNAs deletion

Mitochondrial gene content, arrangement and composition were highly conserved, and mito-genomes typically contained 37 coding genes [3] [~~43~~53]. However, some exceptions, such as gene duplications, deletions or rearrangements, were found in ~~several~~ some species [~~44~~54-~~46~~56]. Although tRNA deletion~~ events~~ ~~were~~is unusual, ~~and only several~~increasing cases have been reported, ~~in~~ such as~~prokaryotes, unicellular organisms [47-48], *Placobdella* [47]~~three~~,~~ amphibians species[54,57], three reptile species [~~51~~58-59], ~~[52],~~ one~~ species of~~ crustacean species[60], and one Hemiptera insect~~s~~ ~~–~~and one Coleoptera insect [~~53~~61], four Psocoptera insects[10,62] and three Mantode*a* insects [~~53~~9]~~– ()~~. In this study, two tRNAs, tRNA-Gln and tRNA-Met, were absent in *E. splendens*~~the~~ mito-genome~~ of *E. splendens*~~. The tRNA deletions was only found in the *E. splendens* mito-genome (this study) in all Blattaria~~Orthopteroidea~~ mito-genomes reported and the tRNA deletion events reported in previous research also scattered in different clade branches, therefore, we consider tRNA deletions appear to be separate events occasionally occurring in some species or evolutionary branches. The deletion of functional genes was obviously disadvantageous for species, and the mechanism of deletion is still unclear~~-~~.

The organism may have a functional replacement to cope with the loss of tRNAs. Two mechanisms were proposed for this functional compensation. The first mechanism where tRNAs from the cytosol are

imported into mitochondria has been confirmed, as aminoacyl-tRNA synthetases being imported from the cytosol into mitochondria [5463], and functional tRNA-Lys encoded in the nuclear genome being imported into marsupial mitochondria [5264]. The second compensation mechanism for the missing tRNAs is ‘superwobble’, where a tRNA with an unmodified U in the wobble position reads all four nucleotides in the third codon position [5565]. In our study, the loss of tRNA-Gln and tRNA-Met can be compensated with their first and second codons matching His and Ile, respectively, and the anticodon swing site U for His and Ile. We calculated the Relative Synonymous Codon Usage (RSCU) values of CAU (H) and AUU (I) in reported cockroach mito-genomes, and discovered that *E. splendens* mito-genome had the highest RSCU values (Table 4), indicating that *E. splendens* might be compensated through tRNA superwobble. Regardless, we cannot exclude tRNA import from the cytosol in *E. splendens* mitochondrion. Therefore, more studies are needed to confirm the compensation mechanism for the absence of tRNAs in the mito-genome of *E. splendens*.

Table 4. The RSCU values of CAU (H) and AUU (I) in known cockroaches

Cockroaches	CAU (H)	AUU (I)	GenBank Accession number
Episymploce splendens	1.69	1.86	OK094023
Blattella germanica	1.54	1.83	NC_012901.1
Blattella bisignata	1.57	1.63	NC_018549.1
Panchlora nivea	1.57	1.77	NC_030002.1
Shelfordella lateralis	1.63	1.63	NC_030003.1

Nauphoeta cinerea	1.49	1.64	NC_035052.1
Cryptocercus meridianus	1.51	1.49	NC_037496.1
Periplaneta australasiae	1.44	1.7	NC_034841.1
Neostylopyga rhombifolia	1.45	1.66	NC_034842.1
Periplaneta brunnea	1.42	1.71	MG010455
Blaptica dubia	1.55	1.54	NC_029224.1
Periplaneta americana	1.36	1.71	NC_016956.1
Gromphadorhina portentosa	1.45	1.42	NC_030001.1
Periplaneta fuliginosa	1.43	1.73	NC_006076.1
Opisthoplatia orientalis	1.35	1.6	NC_029225.1
Cryptocercus relictus	1.21	1.57	NC_018132.1
Cryptocercus kyebangensis	1.33	1.6	NC_030191.1
Eupolyphaga sinensis	0.93	1.64	NC_014274.1

Conclusion

In this study, we sequenced and annotated the *E. splendens* mito-genome. Two tRNAs (tRNA-Gln, tRNA-Met) were ~~absent~~lost and a long intergenic region between tRNA-Val and srRNA (93 bp, with a 64.6% similarity with its corresponding srRNA) was also found in *E. splendens* mito-genome. The duplication/random loss model may account for the origin of this long intergenic spacer. We also found two Numts, Numt-1 and Numt-2 transfered from mitochondrion to nucleus at about 14.15 Ma to 22.35 Ma, and 19.19 Ma to 24.06 Ma respectively.

Acknowledgments

We sincerely appreciate Natural History Museum of Sichuan University for the sample collection. Thanks Dr. Megan for language help. ~~This study was funded by the National Natural Science Foundation of China (U21A20409)the Science and Technology Project of Sichuan Province~~

~~(2017SZ0019), Special funds for central government to guide local scientific and Technological Development (2020ZYD098) .~~

Author Contributions

Conceptualization: XYZ JNM BSY.

Funding acquisition: XYZ.

Investigation: LY ZZH HMW JG QC CJZ.

Resources: LY BSY XYZ JG CJZ.

Writing – original draft: LY ZZH HMW JG QC.

Writing – review & editing: LY XYZ JNM BSY.

References

[1] Wilson AJ, Xu J. Mitochondrial inheritance: diverse patterns and mechanisms with an emphasis on fungi. Mycology. 2012; 3(2): 158-166. doi: 10.1080/21501203.2012.684361. PMID: 7219536.

[2] Ojala D, Montoya J, Attardi G. tRNA punctuation model of RNA processing in human mitochondria. Nature. 1981; 290(5806): 470-474. doi: 10.1038/290470a0. PMID: 7219536.

[3] Boore JL. Animal mitochondrial genomes. Nucleic Acids Res. 1999; 27(8): 1767-1780. doi: 10.1093/nar/27.8.1767. PMID: 10101183.

[4] Li WJ, Wang ZQ, Che YL, The Complete Mitogenome of the Wood-Feeding Cockroach Cryptocercus meridianus (Blattodea:

Cryptocercidae) and Its Phylogenetic Relationship among Cockroach Families. Int. J. Mol. Sci. 2017; 18, 2397. doi: 10.3390/ijms18112397. PMID: 29137151. PMCID: PMC5713365.

[5] Gong R, Guo X, Ma J, Song X, Shen Y, Geng F, et al. Complete mitochondrial genome of *Periplaneta brunnea* (Blattodea: Blattidae) and phylogenetic analyses within Blattodea. J Asia-Pac Entomol. 2018; 21(3): 885-895. doi: 10.1016/j.aspen.2018.05.006.

[6] Du C, Zhang L, Lu T, Ma J, Chen Z, Yue B, et al. Mitochondrial genomes of blister beetles (Coleoptera, Meloidae) and two large intergenic spacers in Hycleus genera. BMC Genomics. 2017; 18(1): 698. doi: 10.1186/s12864-017-4102-y. PMID: 28874137.

[7] Simon S, Hadrys H. A comparative analysis of complete mitochondrial genomes among Hexapoda. Molecular Phylogenetics and Evolution.2013; 69(2) :393–403. doi: 10.1016/j.ympev.2013.03.033. PMID: 23598069.

[8] Chen ZT, Du YZ. Rearrangement of mitochondrial genome in insects. Journal of Environmental Entomology. 2016; 38 (4) : 843 — 851.(In Chinese).

[9] Shi Y, Li LY, Liu QP, Ali MY, Yuan ZL, Guy S et al. Complete mitochondrial genomes of four species of praying mantises (Dictyoptera, Mantidae) with ribosomal second structure, evolutionary and phylogenetic analyses.PLoS ONE. 2021;16(11): e0254914. doi:

10.1371/journal.pone.0254914. PMID: 34735444. PMCID: PMC8568281.

[10] Shi Y, Chu Q, Wei DD, Qiu YJ, Shang F, Dou W, Wang JJ. The mitochondrial genome of booklouse, Liposcelis sculptilis (Psocoptera: Liposcelididae) and the evolutionary timescale of Liposcelis. Scientific RepoRts.2016;6:30660. doi: 10.1038/srep30660. PMID: 27470659. PMCID: PMC4965752.

[11] Shao RF, Zhu XQ, Barker SC, Herd K. Evolution of extensively fragmented mitochondrial genomes in the lice of humans. Genome Biol Evol. 2012; 4(11): 1088–1101.doi:10.1093/gbe/evs088. PMID: 23042553. PMCID: PMC3514963.

[12] Chen SC, Wei DD, Shao RF, Shi JX, Dou W, Wang JJ. Evolution of multipartite mitochondrial genomes in the booklice of the genus Liposcelis (Psocoptera). BMC Genomics. 2014; 15(1):861. doi: 10.1186/1471-2164-15-861. PMID: 25282613. PMCID: PMC4197233.

[13] Zhang LP, Ma Y, Yu DN, Storey KB, Zhang JY. The mitochondrial genomes of Statilia maculata and S. nemoralis (Mantidae: Mantinae) with different duplications of trnR genes. Int J Biol Macromol. 2019;121:839–845.https://doi.org/10.1016/j.ijbiomac.2018.10.038.PMID: 30340009.

[14] Ye F, Lan XE, Zhu WB, You P. Mitochondrial genomes of praying mantises (Dictyoptera, Mantodea): rearrangement, duplication, and

reassignment of tRNA genes. Scientific RepoRts.2016;.6:25634. doi: 10.1038/srep25634. PMID: 27157299.PMCID: PMC4860592.

[3][15] Wang SS, Hou FX, Cao J, Peng C, Guo JL. The complete mitochondrial genome of the Statilia maculate. (Mantodea: Mantidae). Mitochondrial DNA Part B Resources.2016;1(1):860-861.doi: 10.1080/23802359.2016.1250134.PMID:33490423.PMCID:PMC7800993.

[4][16] Ma JN, Du C, Zhou C, Shen YM, Fan ZX, Yue BS, et al. Complete mitochondrial genomes of two blattid cockroaches, *Periplaneta australasiae* and *Neostylopyga rhombifolia*, and phylogenetic relationships within the Blattaria. PLoS One. 2017; 12(5): e0177162. doi: 10.1371/journal.pone.0177162. PMID: 28486518.

[17] Bourguignon T, Tang Q, Ho Simon Y.W., Juna F, Wang ZQ, Arab D A., Cameron S L., Walker J, Rentz D, Evans T A., Lo N. Transoceanic Dispersal and Plate Tectonics Shaped GlobalCockroach Distributions: Evidence from Mitochondrial Phylogenomics. Molecular biology and evolution. 2018; 35(4):970-983. doi: 10.1093/molbev/msy013. PMID: 29420807.

[5][18] Li TT, Liu DX, Qiu DY, Yue QY. Two new species of Episymploce Bey-Bienko, 1950 (Blattodea, Ectobiidae, Blattellinae) from China. Zookeys. 2020; 954:31-45. doi: 10.3897/zookeys.954.49738. PMID: 32821203; PMCID: PMC7406546.

~~[6]~~[19] Wang Z, Che Y. Three new species of cockroach genus *Symploce* Hebard, 1916 (Blattodea, Ectobiidae, Blattellinae) with redescriptions of two known species based on types from Mainland China. Zookeys. 2013; 337(337): 1-18. doi: 10.3897/zookeys.337.5770. PMID: 24146575.

~~[7]~~[20] Xiao B, Chen AH, Zhang YY, Jiang GF, Hu CC, Zhu CD. Complete mitochondrial genomes of two cockroaches, *Blattella germanica* and *Periplaneta americana*, and the phylogenetic position of termites. Curr Genet. 2012; 58(2): 65-77. doi: 10.1007/s00294-012-0365-7. PMID: 22311390.

~~[8]~~[21] Chen AH. Complete mitochondrial genome of the double-striped cockroach *Blattella bisignata* (Insecta: Blattaria: Blaberoidea). Mitochondrial DNA. 2013; 24(1): 14-6. doi: 10.3109/19401736.2012.710228. PMID: 22897805.

~~[9]~~[22] Lopez JV, Yuhki N, Masuda R, Modi W, O'Brien SJ. Numt, a recent transfer and tandem amplification of mitochondrial DNA to the nuclear genome of the domestic cat. J Mol Evol. 1994; 39(2): 174-90. doi: 10.1007/BF00163806. PMID: 7932781.

~~[10]~~[23] Arctander P. Comparison of a mitochondrial gene and a corresponding nuclear pseudogene. Proc Biol Sci. 1995; 262(1363): 13-9. doi: 10.1098/rspb.1995.0170. PMID: 7479989.

~~[11]~~[24] Laslett D, Canbäck B. ARWEN: a program to detect tRNA genes in metazoan mitochondrial nucleotide sequences. Bioinformatics. 2008; 24(2): 172-5. doi: 10.1093/bioinformatics/btm573. PMID: 18033792.

~~[12]~~[25] Tamura K, Peterson D, Peterson N, Stecher G, Nei M, Kumar S. MEGA5: molecular evolutionary genetics analysis using maximum likelihood, evolutionary distance, and maximum parsimony methods. Mol Biol Evol. 2011; 28(10): 2731-2739. doi: 10.1093/molbev/msr121. PMID: 21546353; PMCID: PMC3203626.

~~[13]~~[26] Perna NT, Kocher TD. Patterns of nucleotide composition at fourfold degenerate sites of animal mitochondrial genomes. J Mol Evol. 1995; 41(3): 353-358. PMID: 7563121.

~~[14]~~[27] Drummond AJ, Rambaut A. BEAST: Bayesian evolutionary analysis by sampling trees. BMC Evol Biol. 2007; 7: 214. doi: 10.1186/1471-2148-7-214

~~[15]~~[28] Gernhard T. The conditioned reconstructed process. J Theor Biol. 2008; 253(4): 769-778. doi: 10.1016/j.jtbi.2008.04.005. PMID: 18538793.

~~[16]~~[29] Wolfe JM, Daley AC, Legg DA, Edgecombe GD. Fossil calibrations for the arthropod Tree of Life. Earth-Science Reviews, 2016; 160:43-110.

~~[17]~~[30] Evangelista DA, Wipfler B, Bethoux O, Donath A, Fujita M, Kohli MK, et al. An integrative phylogenomic approach illuminates the

evolutionary history of cockroaches and termites (Blattodea). Proceedings of the Royal Society. Biological sciences, 2019, 286(1895). DOI:10.1098/rspb.2018.2076.

[18][31] Vrsˇansky P. Origin and the early evolution of mantises. AMBA Projekty. 2002; 6(1): 1-16.

[19][32] Dotson EM, Beard CB. Sequence and organization of the mitochondrial genome of the Chagas disease vector, *Triatoma dimidiata*. Insect Mol Biol. 2001; 10(3): 205-215. PMID: 11437912.

[33] Crozier RH, Crozier YC. The mitochondrial genome of the honeybee *Apis mellifera*: complete sequence and genome organization. Genetics. 1993; 133(1): 97-117. PMID: 8417993; PMCID: PMC1205303.

[20][34] Zischler H, Geisert H, von Haeseler A, Pääbo S. A nuclear 'fossil' of the mitochondrial D-loop and the origin of modern humans. Nature.1995; 30;378(6556):489-92.doi: 10.1038/378489a0.PMID: 7477404,

[21][35] Tourmen Y, Baris O, Dessen P, Jacques C, Malthièry Y, Reynier P. Structure and chromosomal distribution of human mitochondrial pseudogenes. Genomics. 2002; 80(1): 71-7. doi: 10.1006/geno.2002.6798. PMID: 12079285.

[22][36] Hazkani-Covo E, Sorek R, Graur D. Evolutionary dynamics of large numts in the human genome: rarity of independent insertions and

abundance of post-insertion duplications. J Mol Evol. 2003; 56(2): 169-74. doi: 10.1007/s00239-002-2390-5. PMID: 12574863.

~~[23]~~[37] Wei SJ, Tang P, Zheng LH, Shi M, Chen XX. The complete mitochondrial genome of *Evania appendigaster* (Hymenoptera: Evaniidae) has low A+T content and a long intergenic spacer between atp8 and atp6. Mol Biol Rep. 2010; 37(4): 1931-42. doi:10.1007/s11033-009-9640-1. PMID:19655273.

~~[24]~~[38] Rodovalho CM, Lyra ML, Ferro M, Jr MB. The mitochondrial genome of the leaf-cutter ant *Atta laevigata*: a mitogenome with a large number of intergenic spacers. PLoS One. 2014; 9(5): e97117. doi: 10.1371/journal.pone.0097117. PMID: 24828084.

~~[25]~~[39] Dotson EM, Beard CB. Sequence and organization of the mitochondrial genome of the Chagas disease vector, *Triatoma dimidiata*. Insect Mol Biol. 2001; 10(3): 205-215. PMID: 11437912.

~~[26]~~[40] Hua J, Li M, Dong P, Cui Y, Xie Q, Bu W. Comparative and phylogenomic studies on the mitochondrial genomes of Pentatomomorpha (Insecta: Hemiptera: Heteroptera). BMC Genomics. 2008; 9(1): 1-15. doi: 10.1186/1471-2164-9-610. PMID: 19091056; PMCID: PMC2651891.

~~[27]~~[41] Cheng XF, Zhang LP, Yu DN, Storey KB, Zhang JY. The complete mitochondrial genomes of four cockroaches (Insecta: Blattodea)

and phylogenetic analyses within cockroaches. Gene. 2016; 586(1): 115-122. doi: 10.1016/j.gene.2016.03.057. PMID: 27045773.

~~[28]~~[42] Bae JS, Kim I, Sohn HD, Jin BR. The mitochondrial genome of the firefly, *Pyrocoelia rufa*: complete DNA sequence, genome organization, and phylogenetic analysis with other insects. Mol Phylogenet Evol. 2004; 32(3): 978-85. doi: 10.1016/j.ympev.2004.03.009. PMID: 15288070.

~~[29]~~[43] Sheffield NC, Song H, Cameron SL, Whiting MF. A comparative analysis of mitochondrial genomes in Coleoptera (Arthropoda: Insecta) and genome descriptions of six new beetles. Mol Biol Evol. 2008; 25(11): 2499-2509. doi: 10.1093/molbev/msn198. PMID: 18779259.

~~[30]~~[44] Stanton DJ, Daehler LL, Moritz CC, Brown WM. Sequences with the potential stem-and-Ioop structures are associated with coding-region duplications in animal mitochondrial DNA. Genetics. 1994; 137(1): 233-241. doi: 10.1101/gad.8.9.1131. PMID: 8056313.

~~[31]~~[45] Moritz C, Dowling TE, Brown WM. Evolution of Animal Mitochondrial DNA: Relevance for Population Biology and Systematics. Annu Rev Ecol Evol S. 1987; 18: 269-292. doi: 10.1146/annurev.ecolsys.18.1.269

~~[32]~~[46] Boore JL. Comparative Genomics: The duplication/random loss model for gene rearrangement exemplified by mitochondrial genomes of Deuterostome animals. 1st ed. Dordrecht: Springer; 2000.

~~[33]~~[47] Sayadi A, Immonen E, Tellgren-Roth C, Arnqvist G. The Evolution of Dark Matter in the Mitogenome of Seed Beetles. Genome Biol Evol. 2017; 9(10): 2697-2706. doi: 10.1093/gbe/evx205. PMID: 29048527; PMCID: PMC5737749.

~~[34]~~[48] Macey JR, Larson A, Ananjeva NB, Fang Z, Papenfuss TJ. Two novel gene orders and the role of light-strand replication in rearrangement of the vertebrate mitochondrial genome. Mol Biol Evol. 1997; 14(1): 91-104. doi: 10.1093/oxfordjournals.molbev.a025706. PMID: 9000757.

~~[35]~~[49] McKnight ML, Shaffer HB. Large, rapidly evolving intergenic spacers in the mitochondrial DNA of the salamander family Ambystomatidae (Amphibia: Caudata). Mol Biol Evol. 1997; 14(11): 1167-76. doi: 10.1093/oxfordjournals.molbev.a025726. PMID: 9364774.

~~[36]~~[50] Burger G, Gray MW, Lang BF. Mitochondrial genomes: anything goes. Trends Genet. 2003; 19(12): 709-16. doi: 10.1016/j.tig.2003.10.012. PMID: 14642752.

~~[37]~~[51] Cha SY, Yoon HJ, Lee EM, Yoon MH, Hwang JS, Jin BR, et al. The complete nucleotide sequence and gene organization of the mitochondrial genome of the bumblebee, *Bombus ignitus* (Hymenoptera: Apidae). Gene. 2007; 392(1-2): 206-20. doi: 10.1016/j.gene.2006.12.031. PMID: 17321076.

[38][52] Jørgensen TE, Bakke I, Ursvik A, Andreassen M, Moum T, Johansen SD. An evolutionary preserved intergenic spacer in gadiform mitogenomes generates a long noncoding RNA. BMC Evol Biol. 2014; 14: 182. doi: 10.1186/s12862-014-0182-3. PMID: 25145347; PMCID: PMC4236577.

[39][53] Gemmell NJ, Janke A, Western PS, Watson JM, Pääbo S, Graves JA. Cloning and characterization of the platypus mitochondrial genome. J Mol Evol. 1994; 39(2): 200-5. doi: 10.1007/BF00163808. PMID: 7932783.

[40][54] Zhang JF, Nie LW, Wang Y, Hu LL. The complete mitochondrial genome of the large-headed frog, *Limnonectes bannaensis* (Amphibia: Anura), and a novel gene organization in the vertebrate mtDNA. Gene. 2009; 442(1-2): 119-27. doi: 10.1016/j.gene.2009.04.018. Epub 2009 May 3. PMID: 19397958.

[41][55] Hlaing T, Tun-Lin W, Somboon P, Socheat D, Setha T, Min S, Chang MS, Walton C. Mitochondrial pseudogenes in the nuclear genome of *Aedes aegypti* mosquitoes: implications for past and future population genetic studies. BMC Genet. 2009; 10: 11. doi: 10.1186/1471-2156-10-11. PMID: 19267896; PMCID: PMC2660364.

[56] von Nickisch-Rosenegk M, Brown WM, Boore JL. Complete sequence of the mitochondrial genome of the tapeworm *Hymenolepis diminuta*: gene arrangements indicate that Platyhelminths are

Eutrochozoans. Mol Biol Evol. 2001; 18(5): 721-30. doi: 10.1093/oxfordjournals.molbev.a003854. PMID: 11319256.

[57] San MD, Gower DJ, Oommen OV, Wilkinson M, Zardoya R. Phylogeny of caecilian amphibians (Gymnophiona) based on complete mitochondrial genomes and nuclear RAG1. Mol Phylogenet. 2004; 33(2): 413-27. doi:10.1016/j.ympev.2004.05.014.

[58] Peng QL, Nie LW, Pu YG. Complete mitochondrial genome of Chinese big-headed turtle, Platysternon megacephalum, with a novel gene organization in vertebrate mtDNA. Gene. 2006; 380(1): 14-20. doi: 10.1016/j.gene.2006.04.001. PMID: 16842936.

[59] Kumazawa Y, Miura S, Yamada C, Hashiguchi Y. Gene rearrangements in gekkonid mitochondrial genomes with shuffling, loss, and reassignment of tRNA genes. BMC Genomics. 2014; 15: 1–13. doi: 10.1186/1471-2164-15-930. PMID: 25344428.PMCID: PMC4223735.

[60] Kilpert F, Podsiadlowski L. The complete mitochondrial genome of the common sea slater, Ligia oceanica (Crustacea, Isopoda) bears a novel gene order and unusual control region features. BMC Genomics. 2006; 7: 241. doi: 10.1186/1471-2164-7-241. PMID: 16987408.PMCID: PMC1590035.

[61] Dai Y, Li L, Jiang P, Song F, Ye Z, Yuan X, et al. Sequence and organization of the mitochondrial genome of an urostylidid bug,

Urochela quadrinotata Reuter (Hemiptera: Urostylididae). Entomotaxonomia, 2012; 34, 613-623,

[62] Wei DD, Shao R, Yuan ML, Dou W, Barker SC, Wang JJ. The multipartite mitochondrial genome of Liposcelis bostrychophila: insights into the evolution of mitochondrial genomes in bilateral animals. PLoS One. 2012; 7(3): e33973. doi: 10.1371/journal.pone.0033973. PMID: 22479490; PMCID: PMC3316519.

[63] Duchêne AM, Pujol C, Maréchal-Drouard L. Import of tRNAs and aminoacyl-tRNA synthetases into mitochondria. Curr Genet. 2009; 55(1): 1-18. doi: 10.1007/s00294-008-0223-9. PMID: 19083240.

[64] Dorner M, Altmann M, Paabo S, Morl M. Evidence for import of a lysyl-tRNA into marsupial mitochondria. Mol Biol Cell. 2001; 12(9): 2688-2698. doi: 10.1091/mbc.12.9.2688.

~~[42]~~[65] Rogalski M, Karcher D, Bock R. Superwobbling facilitates translation with reduced tRNA sets. Nat Struct Mol Biol. 2008; 15(2): 192-8. doi: 10.1038/nsmb.1370. PMID: 18193063.

~~[43] Adams KL, Palmer JD. Evolution of mitochondrial gene content: gene loss and transfer to the nucleus. Mol Phylogenet Evol. 2003; 29(3): 380-95. doi: 10.1016/s1055-7903(03)00194-5. PMID: 14615181.~~

~~[44] Tran TT, Belahbib H, Bonnefoy V, Talla E. A. Comprehensive tRNA Genomic Survey Unravels the Evolutionary History of tRNA Arrays in~~

Prokaryotes. Genome Biol Evol. 2015; 8(1): 282-95. doi: 10.1093/gbe/evv254. PMID: 26710853; PMCID: PMC4758250.

[45] Jiménez-Armenta J, Kvist S, Oceguera-Figueroa A. An exceptional case of mitochondrial tRNA duplication-deletion events in blood-feeding leeches. Org Divers Evol. 2020; 20: 221-231. doi: 10.1007/s13127-020-00431-6

[46] San MD, Gower DJ, Oommen OV, Wilkinson M, Zardoya R. Phylogeny of caecilian amphibians (Gymnophiona) based on complete mitochondrial genomes and nuclear RAG1. Mol Phylogenet. 2004; 33(2): 413-27. doi:10.1016/j.ympev.2004.05.014

[47] Peng QL, Nie LW, Pu YG. Complete mitochondrial genome of Chinese big-headed turtle, *Platysternon megacephalum*, with a novel gene organization in vertebrate mtDNA. Gene. 2006; 380(1): 14-20. doi: 10.1016/j.gene.2006.04.001. PMID: 16842936.

[48] Dorner M, Altmann M, Paabo S, Morl M. Evidence for import of a lysyl-tRNA into marsupial mitochondria. Mol Biol Cell. 2001; 12(9): 2688-2698. doi: 10.1091/mbc.12.9.2688

[49] .

[50] Duchêne AM, Pujol C, Maréchal-Drouard L. Import of tRNAs and aminoacyl-tRNA synthetases into mitochondria. Curr Genet. 2009; 55(1): 1-18. doi: 10.1007/s00294-008-0223-9. PMID: 19083240.

~~[51] Rogalski M, Karcher D, Bock R. Superwobbling facilitates translation with reduced tRNA sets. Nat Struct Mol Biol. 2008; 15(2): 192-8. doi: 10.1038/nsmb.1370. PMID: 18193063.~~

Supporting Information

S1 Table. GenBank accession numbers for divergence date analyses.

S2 Table. Nucleotide composition in different regions of *Episymploce splendens*.

S3 Table. Relative synonymous codon usage (RSCU) for PCGs of *Episymploce splendens*.

S4 Table. The length and similarity of these large intergenic spacers with their original genes in some insects. The similarity was calculated by DNAMAN.

Fig. S1 Putative secondary structures of the 20 tRNA genes identified in *Episymploce splendens*.

Fig. S2 Aligement result of the long intergenic spacer and srRNA of *Episymploce splendens* by MEGA.

Fig. S3 Similarity comparison results of Numt-1 and mito-genome of *Episymploce splendens* by DNAMAN.

Fig. S4 Similarity comparison results of Numt-2 and mito-genome of *Episymploce splendens* by DNAMAN.

Response to Reviewers

Dear editor:

Thank you for giving us the opportunity to revise our manuscript entitled "Complete mitochondrial genome of *Episymploce splendens* (Blattodea: Ectobiidae): a large intergenic spacer and lacking of two tRNA genes" to PLOS ONE. We appreciate that the reviewers′ valuable comments for our manuscript. These comments are very important for improving our manuscript. We have revised our manuscript according to the reviewers′ comments and also made other improvement for our manuscript. We do hope that our manuscript is appropriate and could be considered for publication in this journal.

Best regards,

Sincerely yours,

Xiuyue Zhang, PhD and Professor

College of Life Sciences, Sichuan University

Email: zhangxy317@126.com

Reviewers' comments:

Reviewer 2:

This round you responded all the comments carefully, and deleted the meaningless or not closely related part. it feels much more compact and focused. I suggest emphasizing the existence of pseudogenes (numts) and the time when pseudogenes appear in this cockroach, instead of a large intergenic spacer. And I think the introduction to the study of the cockroach mitochondrial genome is too simplistic.

Response:

Thanks for your suggestion. The mito-genome commonly displays exceptional economy of organization and the large intergenic spacer was uncommon. We believed the find of large intergenic spacer in mitochondrial genome of *Episymploce splendens*

was important for the study of insect mitochondrial genome evolution in the future and so we retained it. And based on your suggests, we also further emphasized the discovery of numts and their significance, and added a description of the mitochondrial genome and enriched the content.

Reviewer 3:

1.I failed to remember that I have reviewed this paper before. I failed to find this mt genome of OK094023 in NCBI. I need this sequence to judge the mt genome right or not! If I reviewed it before, I must be concerned on this question. I did not believe two trnQ and trnM lost in Episymploce splendens.

The two trnQ f and trnM can be translated into between two CRs. As the authors said that this is the first report of the tRNAs deletion in Orthopteroidea mito-genomes. But I did not believe it. I suggest the authors check it carefully using the other species in genus Episymploce. Because if the mt genome is existed two similar CRs, the lost genes will be found between two CRs.

I suggest the authors should be carefully checked again.

When the authors used a pair of primers of "Es11 13822-CAGATTATATTGATTCGCACAAC" and "303-ATAGAACTGATGAAGCTAAGGC" to amplify PCR using 75 second Extension. It may be failed to amplify two control regions. I suggest the authors can design more primers to check it.

Response:

Thanks for your careful reading.

We really appreciate your valuable time on reviewing our manuscript. We had provided the complete mt-genome to Genbank and obtained the GenBank identifier (OK094023) but we set up the release date, and so this sequence cannot be found in NCBI at present. However, in order to the convenience of review, it has been uploaded as an attachment in our submitted manuscript and also in our revised manuscript.

The non-coding region of *E. splendens* mito-genome was located between srRNA

and tRNA-Ile, and it was 290 bp long consist of two 120 bp long repeated units. The two lost tRNAs were located behind tRNA-Ile, and the interval between tRNA-Ile and NAD2 was only 24bp. They cannot form the 2 tRNAs. Because we used ARWEN and tRNA-SE to identify tRNAs and obtained same result through reference to secondary structure models for those genes from other blattarian insects (Ma et al., 2017; Xiao et al., 2012). The tRNA-Gln and tRNA-Met located between tRNA-Ile and NAD2, and they were typically 150bp long in other cockroaches. However, this site was only 24bp long in *E. splendens*. In addition, we tried to refer to other cockroach mitochondrial genomes, but however, there was no mitochondrial sequences reported in *Episymploce* in NCBI. In reported cockroach mitochondrial genomes, we did not find two similar CRs, namely, these reported cockroach mitochondrial genomes have only one A-T region. We designed different primers (in the table) to amplify this area and obtain same amplification sequences, so we think our experiment result was correct and there was no sequence lost due to inappropriate primers. We could not found the secondary structures of two tRNAs in *E. splendens* mito-genome sequence, and so we believed their loss event. Although tRNA lacking was rare, other species had been found in previous studies. For example, *Limnonectes bannaensis* mt-genome lost 4 tRNAs (tRNA-Ala, tRNA-Asn, tRNA-Cys and tRNA-Glu), *Gegeneophis ramaswamii* mt-genome lost tRNA-Phe, *Urochela quadrinotata* Reuter mt-genome lost 2 tRNAs (tRNA-Ile and tRNA-Gln), *Platysternon megacephalum* mt-genome lost tRNA-Thr. These loss events of tRNAs occur accidentally and independently in these mt-genome, and we also believe that these findings will be useful for future studies on the cause of their loss and the subsequent adaptive evolution.

	F	R
1.ES11	CAGATTATATTGATTCGCACAAC	ATAGAACTGATGAAGCTAAGGC
2.(Ma et al,2017)	CCTCTAAAAAGACTAAAATACCGCC	GGAATCATCAGTGAAAGGGAGC
3.	AATAAAGAGTGCCCCTGTC	GATATTACAGGGATGAAGGATA
4.	TAAAGAGTGCCCCTGTC	TGATGAAGCTAAGGCTTGAA

2. Two Numts were found by PCR. Two Numts were similar to lrRNA, which can be

explained why the author failed to obtained trnQ and trnM. Check the mt genome again!

Another question is how the authors judge the two Numts？ Why is not lrRNA belonging to Numt？

I did think the divergence time of Numts are interesting. Because the title is "Complete mitochondrial genome of Episymploce splendens (Blattodea: Ectobiidae): a large intergenic spacer and lacking of two tRNA genes" not including Numts.

Response:

The sequences which originated from the invasion of mtDNA into nuclear DNA were named Numts. Numts exist in the nuclear DNA and do not belong to the mitochondrial genome sequence, but due to their partial sequences may be identical to their source sequences, some primers that amplify mitochondrial genomes may accidentally amplify these sequences. So, two Numts may be not related to the loss of tRNAs.

Primers that amplified mitochondrial genes may occasionally amplify Numts. In this study, agarose gel electrophoresis of the amplification product (the primer Numt-1-1 and Numt-2-1, Table 1) appeared as two bands. We then redesigned primers (Numt-1-2 and Numt-2-2, Table 1) to amplify these regions and obtained the same result. These non-targeted bands were purified and sequenced, and obtained sequences belonging to mitochondria via the analysis of NCBI blast. Due to differences in mutation rates or relaxation from selection pressure, they had some degree of base differences compared to corresponding mito-genome sequences. These sequences have more mutations of insertion and deletion compared to their corresponding mitochondrial sequences, and may lose their functions. They did not belong to the mitochondrial genome, so they were not the lost tRNAs. We had explained this in the corresponding section of the manuscript (Page line 162). Numts can be used to solve problems in phylogeny, but they were not a part of the mitochondria, so we did not mentioned them in the title.

3.Is a large intergenic spacer similar to trnI or trnQ? or AT-rich region?

Response:

The large intergenic spacer was not A-T region or similar to trnI or trnQ. It was similar to its corresponding srRNA with a 64.6% similarity.

4.I read the paper and found the English writing is really poor. There are many mistaken in the paper. I can not believe the paper is revsied the second version.

Response:

we're sorry for the writing mistaken and we have made careful revisions to our English writing.

5.All genes name should be the same in text, figures, and tables.

Response:

We have made relevant changes in our revised manuscript.
