# Supplementary material for: Complete mitochondrial genome of Episymploce splendens (Blattodea: Ectobiidae): A large intergenic spacer and lacking of two tRNA genes
Source: PLoS One. 2022 Jun 2;17(6):e0268064. doi: 10.1371/journal.pone.0268064 (PMC9162313; doi:10.1371/journal.pone.0268064)
Supplement: Attachment — Submitted filename: Response to comments.docx [file pone.0268064.s011.docx]

**Reviewers' comments:**

**Reviewer 1:**

In this manuscript, the authors sequenced the mitochondrial genome of the cockroach species Episymploce splendens. The mitogenome lacks two tRNAs and has a long intergenic spacer (93 bp long) between tRNA-Val and srRNA. Then, the authors conducted phylogenetic analyses for Blattodea relying on 13 protein coding genes.

I have several concerns regarding this manuscript, from structure issues (especially discussion inserted within the results) to a lack of methodological details and an inadequate (according to me) phylogenetic section.

The authors report the first mitogenome of Blattodea and relatives that lack a couple of tRNAs. This is interesting data to investigate the molecular evolution of mitogenomes in insect. I regret, however, that the authors do not provide the sequences yet, which is, for a reviewer, highly frustrating. I would have love to see the data, look at the annotation and so on. I consider that, for such a manuscript, it should be mandatory to provide this kind of information for the review process. Also, I was curious about the software (ARWEN) used to annotate the mitogenome and, more precisely, how others software might have been used to confirm the results of the authors. This is especially troubling because ARWEN did not recognize 3 tRNAs that the authors had to identify manually. How likely is it that the 2 tRNAs reported here as lacking could have also been missed by ARWEN and the authors?

Note that I am not relevant enough to discuss further the quality and completion of the data and analyses provided regarding the composition of the mitogenomes or the evolutionary mechanisms. Nevertheless, I am a bit skeptical about the dating analyses with NUMTs. I wonder if those analyses really make sense (i.e. aligning mitochondrial sequences with a NUMt, and then trying to estimate divergence ages). As for the phylogenetic part, I simply think that it should be removed from the manuscript and I will try to explain my position here. First, and above all, the data brought here (i.e. 1 mitogenome of a species that undoubtedly belongs to the Ectobiidae family) does not bring enough additional data or new results to justify those phylogenetic analyses. In addition, the authors did not use all the mitogenomes available and we have no idea why some taxa were used while others were disregarded. Second, the vocabulary used (i.e. mainly the word ‘basal’) leads to some very surprising sentences for phylogeneticists, which might reflect a lack of understanding of phylogenetic analyses or at least of the phylogenetics of Blattodea. Third, there are several parameters that are not provided in the manuscript or methodological choices that should be explained in more details (e.g. two Lepidoptera as outgroups? ‘unmatched’ regions removed?). Finally, the literature cited is not up to date and biased with, for instance, a study relying on a single marker cited while the most recent studies are disregarded. Overall, this part of the manuscript is flawed and does more harm than good to this manuscript.

**Response:**

We really appreciate your valuable time on reviewing our manuscript. We provide the complete mt-genome to Genbank and obtained the GenBank accession number (OK094023) although we set up the release date. Whatever, we provide the Genbank annotation format file to facilitate the review, and the annotation information of this mitochondrial genome is also presented in Table 3. Actually, except for ARWEN, we used tRNA-SE to identify tRNAs and obtained identical result. The tRNAs we did not identify by softwares were identified by manually, through reference to secondary structure models for those genes from other blattarian insects (Ma et al., 2017; Xiao et al., 2012). We drew 20 tRNAs in hand, and could not find the remaning 2 tRNAs, tRNA-Gln and tRNA-Met. tRNA-Gln and tRNA-Met located between tRNA-Ile and NAD2, and they were typically 150bp long. However, this site was only 24bp long in *E. splendens* and we also could not found their secondary structures here. We designed differtrnt primers to amplify this area and obtain same amplification sequences, so we think our experiment result was correct. Although tRNA lacking was rare, other species had been found in previous studies. For example, *Limnonectes bannaensis* lost 4 tRNAs, respectively tRNA-Ala, tRNA-Asn, tRNA-Cys and tRNA-Glu. *Gegeneophis ramaswamii* lost tRNA-Phe in mt-genome. *Urochela quadrinotata* Reuter lost 2 tRNAs, respectively tRNA-Ile and tRNA-Gln. *Platysternon megacephalum* lost tRNA-Thr. So we supported the 2 tRNAs were missing in *Episymploce splenden* mt-genome.

Nuclear mitochondrial pseudogenes (Numts) are DNA fragments transferred from mitochondria to the nucleus, which may become non-functional due to mutation and loss of selective pressure. Since the mutation rate in the nucleus is lower than that of the mitochondria, the divergence time can be calculated according to the sequence differences between them and ancient mtDNA. These Numts are equivalent to fossils of mitochondrial DNA left in nuclear DNA, and the divergence time of species can be estimated based on identical or similar Numts in related species. So they can be called “fossil” markers. Numts can be used to solve some problems in phylogeny, such as Zischler et al (1995) used the Numt of D-loop as phylogenetic outgroup to prove the origin of man, Lou et al(2013) used 2 Numts as “fossil” markers to trace back the speciation time of two sibling fig wasp species. So we [consider](D:/Users/Administrator/AppData/Local/youdao/dict/Application/8.9.6.0/resultui/html/index.html#/javascript:;) the research on Numts is meaningful.

In phylogenetic analyses, the absence of some species may lead to [uncertain](D:/Users/Administrator/AppData/Local/youdao/dict/Application/8.9.6.0/resultui/html/index.html#/javascript:;) inferences for phylogenetic relationships (Tomita et al., 2011). Increased samplings can provide more complete and reliable information for phylogenetic analysis. In this study, we amplified the entire mt-genome sequence of *Episymploce* cockroaches for the first time. Performing phylogenetic trees with mt-genomes was used to confirm the taxonomic status of *E.splendens*. At the same time, our phylogenetic analysis also further some phylogenetic relationships, and the reviewer 2 suggested us to use more phylogenetic analysis methods. Therefore, we had retained this part. However, as your comment, this part was not the highlight in our manuscript, so we tried to simplify it and….. Blattaria contains 4,600 described species, but only limited complete mitochondrial genomes have been reported. At present, there are still little known complete mt-genomes of most [cockroach](D:/Users/Administrator/AppData/Local/youdao/dict/Application/8.9.6.0/resultui/html/index.html#/javascript:;)es. Many [cockroach](D:/Users/Administrator/AppData/Local/youdao/dict/Application/8.9.6.0/resultui/html/index.html#/javascript:;)es are unmentioned since their mt-genomes are still unknown. Besides, we are sorry for using the word “basal”. We had changed “basal” in the whole manuscript (page 2, line 40; page 4, line 70; page 16, line 286; page 26, line 493).

Phylogenetic trees that we build are typically unrooted trees and we need some outgroups to root the tree to reflect the ancestral relationships. Outgroup species are usually closely related to and can be separated from the species to be studied. Both Lepidoptera and Orthopteroidea belong to Pterygota, and have closely relationship, so we chose the two Lepidoptera as outgroups. Besides, previous study on the phylogenetic relationships among cockroaches also chose same two Lepidoptera as outgroups (Gong et al., 2018).

“Unmatched” regions couldn't provide any useful information in [phylogenetic](D:/Users/Administrator/AppData/Local/youdao/dict/Application/8.9.6.0/resultui/html/index.html#/javascript:;) [analysis](D:/Users/Administrator/AppData/Local/youdao/dict/Application/8.9.6.0/resultui/html/index.html#/javascript:;), so they were usually deleted during sequence alignment. About the cited literatures, thanks again for your advice and sorry for these careless mistakes. Now we have carefully read and considered your comments, and we had cited the latest litaratures in the manuscript. The following are point by point responses to your comments:

1.Page 2, line 32. What is Orthopterodea? Is it a commonly accepted term for the clade you have in mind?

**Response:**

Thanks for your suggestion and sorry for the careless mistake. We have changed “Orthopterodea” to “Orthopteroidea”.

2. Page 2, line 33. It reads as if you were the first to propose this mechanism. Please rephrase.

**Response:**

Thanks for your suggestion. We changed “proposed” to “[support](D:/Users/Administrator/AppData/Local/youdao/dict/Application/8.9.6.0/resultui/html/index.html#/javascript:;)ed”.

3.Page 2, line 38. I do not understand what you mean by 'molecular fossils'.

**Response:**

Nuclear mitochondrial pseudogenes (Numts) are DNA fragments transferred from mitochondria to the nucleus, which may become non-functional due to mutation and loss of selective pressure. Since the mutation rate in the nucleus is lower than that of the mitochondria, the divergence time can be calculated according to the sequence differences between them and ancient mtDNA. These Numts are equivalent to fossils of mitochondrial DNA left in nuclear DNA, and the time of divergence can be estimated based on identical or similar Numts in related species (Lopez et al., 1994; Thalmann et al., 2004). Besides, Numts can be used to solve problems in phylogeny, such as Zischler et al(1995) used the Numt of D-loop as phylogenetic outgroup to prove the origin of man, Lou et al(2013) used 2 Numts as “fossil” markers to trace back the speciation time of two sibling fig wasp species.

4.There is no 'basal' position. This kind of terms should be banned. Here Blaberoidea is sister-group to Blattoidea, and none of those clades is basal'.

**Response:**

We changed “basal” in the whole manuscript (page 2, line 40; page 4, line 70; page 16, line 286; page 26,line 493).

5.Page 2, line 40. Well, this result is not new and has been discussed at length in the literature. I do not think it should be a highlight of your study.

**Response:**

Thank you for your valuable advice. We have removed "Isoptera should be classified as a family within Blattodea and Mantodea as the sister clade to (Isoptera + Blattodea)." .

6.Page 2, line 44. Well, every new data in this regard is welcome for sure but to say that your study bring "important data for further studies on (.) the phylogenetic relationships of Blattodea" is a huge overstatement. Several tens of mitogenomes are availlable and you provide the community with an additional one.

**Response:**

Thanks for your suggestion. We changed “These discoveries could deepen our understanding of mitochondrial genome of insects, and increase the clarity of Blattodea phylogenetic relationships and contribute to the study of insect mito-genome evolution” to “Our study provided useful data for further studies on insect mito-genome evolution and the phylogenetic relationships of Blattodea.”.

7.Page 3, line 64. Given the species richness of Blattodea, I would say 'a few' rather than 'many (i.e. ca. 20 species, out of >4000)

**Response:**

Thank you for your valuable advice. We have changed “many” to “a few”.

8.Page 4, line 73. Remove?

**Response:**

We haved removed “*Episymploce*”.

9.Page 4, line 77.Sequenced

**Response:**

We have changed “determined” to “sequenced” in our revised manuscript.

10.Page 4, line 82. spacer and not spacers if you found only one as the abstact suggests.

**Response:**

Thanks for your advice and sorry for the careless mistake. We changed “spacers”to “spacer”.

11.Page 5, line 90. I believe five species of Episymploce are known from China mainland (and I guess more await description). Can you give the readers some details on the way you identified the specimens?

**Response:**

Thanks for your suggestion. We added some details that we identified the specimens. The Episymploce species have typical characteristics of adult cockroaches such as two sets of wings, sclerotized tegmina and wider membranous hindwings. Besides, the [pronotum](D:/Users/Administrator/AppData/Local/youdao/dict/Application/8.9.6.0/resultui/html/index.html#/javascript:;) of *E. splendens* is black and one-orange patch is on each side of the lateral border. The front and hind wings are fully developed and through the end of the abdomen. Supra-anal plate of male genitalia is asymmetrical (page 5, line 90).

12.Page 5, line 98. Remove

**Response:**

Thanks for your advice. We delete “it”.

13.Page 5, line 99.Which muscle tissue, please clarity.

**Response:**

Thanks for your advice. We have added the location that muscle tissue was sampled in the manuscript.

14.Page 5, line 109. I do not understand this sentence. How specific primers have been designed, besides comparing the conserved regions of the two Blattella species mentioned before?

**Response:**

Thank you for your careful work. We firstly designed primers using the conserved regions of two Blattella species mito-genomes to amplify some regions of *E. splendens* mito-genome. And then the sequenced regions were used to design specific primers to amplify the remaining sequences of *E. splendens*. Primers were designed by the software Primer Premier 5.

15.Page 6, line 119. what do you mean here by 'estimated"? Please clarify.

**Response:**

Through 1.0% agarose gel electrophoresis, we can [preliminarily](C:/Users/Administrator/AppData/Local/youdao/DictBeta/Application/8.9.6.0/resultui/html/index.html#/javascript:;) [estimate](D:/Users/Administrator/AppData/Local/youdao/dict/Application/8.9.6.0/resultui/html/index.html#/javascript:;) if the PCR pruduct was the target band we want, such as if it was the right size.

16.Page 8, line 126. We do not know to which sequences this 'these' refers to? Is a sentence missing?

**Response:**

Thank you for your careful work. There is no sentence missing. To avoid ambiguity, we changed “these sequences” to “The sequences” in our revised manuscript.

17.what about Mitos or other software classically used for this kind of purpose?And if ARWEN did not find three tRNAs, how sure can we be about the two others that you consider as missing for this species?

**Response:**

Actually, except for ARWEN, we used tRNA-SE to identify tRNAs and obtained similar result. The tRNAs we did not identify by softwares were identified by eye, through reference to secondary structure models for those genes from other blattarian

insects (Ma et al., 2017; Xiao et al., 2012). We drew 20 tRNAs in hand, and could not find the remaning 2 tRNAs, tRNA-Gln and tRNA-Met. tRNA-Gln and tRNA-Met lacated between tRNA-Ile and NAD2, and they were typically 150bp long. However, this site was only 24bp long in *E. splendens*. We designed differtrnt primers to amplify this area and obtain same amplification sequences, so we think our experiment result was correct. We could not found their secondary structures here. Although tRNA lacking was rare, other species had been found in previous studies. For example, *Limnonectes bannaensis* lost 4 tRNAs, respectively tRNA-Ala, tRNA-Asn, tRNA-Cys and tRNA-Glu. *Gegeneophis ramaswamii* lost tRNA-Phe in mt-genome. *Urochela quadrinotata* Reuter lost 2 tRNAs, respectively tRNA-Ile and tRNA-Gln. *Platysternon megacephalum* lost tRNA-Thr. So we supported the 2 tRNAs were missing in *Episymploce splenden* mt-genome.

18.Page 9, line 158. I guess this is not correct as your sampling includes Caelifera and Ensifera.

**Response:**

Thanks for your advice. We changed “8 grasshopper” to “4 grasshoppers, 4 [crickets](https://xueshu.baidu.com/usercenter/paper/show?paperid=1h4y0p80x45n00a04a2w0xf0w9656607&site=xueshu_se)”.

19.Choosing two Lepidoptera as outgroups should be better explained. I think it is a bad choice and the relationships found regarding Orthoptera notably make me think that I might be right.

**Response:**

Phylogenetic trees that we build are typically unrooted trees and we need some outgroups to root the tree to reflect the ancestral relationships. Outgroup species are usually closely related to and can be separated from the species to be studied. Both Lepidoptera and Orthopteroidea belong to Pterygota, and have closely relationship. [Species differentiation of](https://xueshu.baidu.com/usercenter/paper/show?paperid=3415e1894cafe900195950474a907701&site=xueshu_se) them is 143 Ma to 250 Ma, so we chose the two Lepidoptera as outgroups. Besides, previous study on the phylogenetic relationships among cockroaches also chose same two Lepidoptera as outgroups (Gong et al., 2018).

20.Page 9, line 166. What were these regions? How long were they? This should be stated in the manuscript. It is all the more important that we do not expect 'unaligned or unmatched' regions for the PCG！

**Response:**

“Unmatched” regions couldn't provide any useful information in [phylogenetic](D:/Users/Administrator/AppData/Local/youdao/dict/Application/8.9.6.0/resultui/html/index.html#/javascript:;) [analysis](D:/Users/Administrator/AppData/Local/youdao/dict/Application/8.9.6.0/resultui/html/index.html#/javascript:;), so they were usually deleted during sequence alignment. After alignment in MEGA 5.2, we removed the unaligned sequences at both ends of the sequence and in the middle. Because different species had different mitochondrial sequence lengths, so the sequences we deleted were various. The remaining length of nucleotide data of 13 PCGs was 9,923bp, amino acid sequences of 13 PCGs was 10,032bp, and lrRNA+srRNA was 1,375bp.

21.Page 9, line 168. What about the models for the amino acid sequences? You must provide them as well here.

**Response:**

We modified our manuscript and made it more clear. In the advised manuscript, we provided the models for the amino acid sequences. The GTR+I+G model was chosen as the best-fitting model for BI analysis, the GTR model was chosen as the best-fitting model for the ML analysis.

22.What about convergence diagnostics? Did you check for convergence and if yes, how? Please give the readers all these details.

**Response:**

Convergence diagnostics were done after constructing the trees, but we did not show it in the manuscript at first. The log files could be generated after the [construct](https://xueshu.baidu.com/usercenter/paper/show?paperid=e90f67b1500c90247b49d993ef0f8a2a&site=xueshu_se)ion of BI trees and we used them to conduct convergence diagnostics. In these files, we found all average standard deviations were down to 0.01. This showed the results were credible.

23.Page 10, line 181. 'in’ rather than "by. Note that the sentence is awkward because you worte that you modified the trees. I guess you mean that you use FigTree to read the tree, add support values and error bars or these kind of things, but that you did not modify the tree (i.e did not modify the phylogenetic relationships).

**Response:**

Thanks for your careful work. We changed “by” to “in”. Yes, we used Figtree to read our trees, add support values and GenBank accession number to the node and to the corresponding species, [respectively](C:/Users/Administrator/AppData/Local/youdao/DictBeta/Application/8.9.6.0/resultui/html/index.html#/javascript:;). We did not modify the tree, so we changed “modified” to “visualized”.

24.Page 14, line 252. I don't get it. What are melois? How did you compute the values for this Figure 2?

**Response:**

Thanks for your advice and sorry for the careless mistake. It should be “*E. splendens*” instead of “melois”. We put the sequences of 13 PCGs (expect for the stop codon) to MEGA5.2 to compute the RSCU. We made corresponding revision in our revised manuscript.

25.The results section if a mx of results and discussion (as the numerous reference cited in the result section show)

**Response:**

Thanks for your careful work. We modified this part and remove “The TA stop codon is common in insect mitochondrial genomes and it has been suggested that these incomplete stop codons can be completed by adding A during transcription without after translation [2] [46]”.

26.all Lepidoptera I think and I do not see what is the point of discussing this as it was not found in Episymploce splendens.

**Response:**

Thanks for your advice. We had removed “The initiation codons of COI genes were various in different insect species, with typical COI triple start codons including ATG and ATC. However, non-ATN trinucleotide (CGA, TTG) codons, tetranucleotides (ATAA, TTAA, and ATTA) and hexanucleotides (ATTTAA) have been found as the start codons of COI genes in some species [40] [41] [42] [43] [44] [45]”.

27.Again this belongs to the discussion section, not the results

**Response:**

Thanks for your careful work. We remove “The A+T rich region plays an important role in mitochondrial transcription and replication [47] [48]. Since the control region does not code for genes, the degree of variation in the control region is high and the length varies greatly due to its non-coding characteristic [49]. For example, the non-coding region in insects varies from 70 bp in Ruspolia dubia to 9-13 kb in Pissodes spp. [50] [51]” “Repeated units in the control regions were also found in different blattarian insects. However, these repeated sequences had low similarity in various cockroaches and thus may have different origins [4]”.

28.The word basal should be removed from this manuscript. It can be done quite easily. For instance, Mantodea is not basal to... it is sister-group to Blattodea.

**Response:**

Thank you for your valuable advice. We revised the whole manuscript (page 2, line 40; page 4, line 70; page 16, line 286; page 26,line 493).

29.Page 15, line 280. I'd say polyphyletic.

**Response:**

We changed “paraphyletic” to “polyphyletic”.

30.Page 16, line 297. I do not think that it is an appropriate reference. Other works specifically dealt with support values and compare PP and bootstrap values. Those should be cited instead.

**Response:**

Thanks for your [valuable](D:/Users/Administrator/AppData/Local/youdao/dict/Application/8.9.6.0/resultui/html/index.html#/javascript:;) advice. We [refer](D:/Users/Administrator/AppData/Local/youdao/dict/Application/8.9.6.0/resultui/html/index.html#/javascript:;) [to](D:/Users/Administrator/AppData/Local/youdao/dict/Application/8.9.6.0/resultui/html/index.html#/javascript:;) [relevant](D:/Users/Administrator/AppData/Local/youdao/dict/Application/8.9.6.0/resultui/html/index.html#/javascript:;) [literatures](D:/Users/Administrator/AppData/Local/youdao/dict/Application/8.9.6.0/resultui/html/index.html#/javascript:;) and found this is not an absolute conclusion (Cummings et al., 2008), whether bootstrap or posterior probability values are higher depends on the data in support of alternative topologies. So we remove the reference.

31.Page 17, line313. I do not understand this sentence.

**Response:**

We changed “We found that the deletion mutation, base substitution and insertion mutation conformed to Numt-1 and Numt-2” to “some [characteristic](D:/Users/Administrator/AppData/Local/youdao/dict/Application/8.9.6.0/resultui/html/index.html#/javascript:;)s such as the deletion mutation, base substitution and insertion mutation were also found in Numt-1 and Numt-2”.

32.What is this optimal partitioning scheme. What are the parameters of this analysis? What is the rationals for the taxonomic sampling? There is no CI for crown-Dictyoptera and crown-Blattodea. Is it because those ages were fixed (or very very constrained) in the analyses. If yes, then the slight differences in age estimates between the two topologies might reflect these very strong constraints rather than a signal from the data.

**Response:**

Thanks for your advice. This is our poor description and we just want to descript that the best-fit evolution model was determined by PartitionFinder. We changed “An optimal partitioning scheme was determined by PartitionFinder” to “The best-fit evolution model was determined by PartitionFinder”. Beside, the parameters we used were substitution model (GTR), base frequencies (empirical), site heterogeneity model (Gamma), molecular clock model (estimate relaxed clock: uncorrelated lognormal), tree prior (yule process), fossils and MCMC (length of chain:50,000,000; echo state to sereen every: 1000). The rationals of taxonomic sampling based on [known](D:/Users/Administrator/AppData/Local/youdao/dict/Application/8.9.6.0/resultui/html/index.html#/javascript:;) fossils and the kinship between species. According to another reviewr’s suggestion, we reselected fossils to calculate the divergence time and produce confidence intervals. We found in Fig 5(b), the clade (Eupolyphaga sinensis + Blaberoidea) did not produce CI, we guess it may be caused by lack of [significance](D:/Users/Administrator/AppData/Local/youdao/dict/Application/8.9.6.0/resultui/html/index.html#/javascript:;) [level](D:/Users/Administrator/AppData/Local/youdao/dict/Application/8.9.6.0/resultui/html/index.html#/javascript:;). You can see these information in Page 10, line 191.

33.Page19, line 354. I do no really understand the rationale here. And why mentioning the realtively long intergenic spacer in Blaptica dubia? What does it suggest?

**Response:**

According to slipped-strand mispairing theory, mispairing involves dissociation of replicating DNA strands and then misaligned reassociation, following replication or repair lead to insertions of several repeat units. Formed tandem repeat experienced random loss and/or point mutation, only the repeat units in both ends are completely retained and the residues form the long spacer (Du et al, 2017). We could not find a homologous sequence with both ends in this intergenic spacer in *E.splendens*, so we think this region is not formed by this model. Besides, similar long intergenic spacer in *Blaptica dubia* had been reported and its formation was [speculated](D:/Users/Administrator/AppData/Local/youdao/dict/Application/8.9.6.0/resultui/html/index.html#/javascript:;) by the duplication/random loss model. So we assumed that this long intergenic spacer in *E.splendens* was generated by the duplication/random loss model.

34.I think Fig. 6 is useless.

**Response:**

We believe that Fig. 6 is a useful illustration of the formation of the long intergenic spacer, and we keep this figure in our manuscript for readers to understand.

35.Page 20, line 384. I am not sure to understand what you mean by 'root sequences' and how they were computed. And from here starts a long section with a single reference about beetle mitogenomes. Do you have any references to support your claims or is it purely speculative?

**Response:**

The long spacers listed in S5 Table were [originate](D:/Users/Administrator/AppData/Local/youdao/dict/Application/8.9.6.0/resultui/html/index.html#/javascript:;)d [from](D:/Users/Administrator/AppData/Local/youdao/dict/Application/8.9.6.0/resultui/html/index.html#/javascript:;) protein-coding genes in mt-genomes, they have a certain similarity. So we called the original sequences of protein-coding genes root sequences. MEGA5.2 was used to compare the long spacer region with the protein-coding genes to determine the root sequence. The similarity between the root sequence and long spacer was computed by the software DNAMAN. Through the length comparison, similarity comparison and spacer locations, we carefully set out our speculation in the manuscript.

36.Page 21, line 405. What were the tRNAs lost in these different species? Is it a single tRNA in each species or are there several tRNAs in some of these species?

**Response:**

The amount of tRNAs lost varies among species. For example, *Limnonectes bannaensis* lost 4 tRNAs (tRNA-Ala, tRNA-Asn, tRNA-Cys and tRNA-Glu), *Gegeneophis ramaswamii* lost tRNA-Phe in mt-genome, *Urochela quadrinotata* Reuter lost 2 tRNAs (tRNA-Ile and tRNA-Gln), *Platysternon megacephalum* lost tRNA-Thr.

37.Please remove this. This has been repeatedly published, discussed, with data specifically designed to address this issue, which is not the case for your study. I think the whole the phylogenetic section should be deleted.

**Response:**

We deleted “The Blattodea, Isoptera and Mantodea all belonged to Dictyoptera based on morphological and anatomical characteristics [11] [12]. According to molecular and morphology data, Mantodea was considered as the basal split within Dictyoptera [81] [82]. However, DeSalle et al [83] supported that Mantodea and Isoptera were sister groups within Dictyoptera. Nevertheless, more recent studies discovered that Mantodea was the sister clade to (Isoptera + Blattodea), and even indicated that Isoptera should be classified as a family (Termitidae) within Blattodea [34] [39] [84]. In our study, all results supported Mantodea was a sister clade to Blattodea (including Blattodea and Isoptera) with high support. In addition, we found Isoptera clustered into a [monophyletic](C:/Users/Administrator/AppData/Local/youdao/DictBeta/Application/8.9.3.0/resultui/html/index.html#/javascript:;) clade with high support (Fig 4 and S3 Fig), and this was also supported by several previous studies [9] [12] [85]. Consequently, we propose that Isoptera should be classified as a family within Blattodea”.

38.Page 23, line 446. citing here a paper listing the cockroaches from Colombia is very very weird!

**Response:**

We cited [Djernæs](https://www.researchgate.net/profile/Marie-Djernaes) (2018) instead of Velez et al (2006).

39.Page 24, line 459. is, not was

**Response:**

We changed “was” to “is”.

40.Page 24, line 447. this is a very biased interpretation of the situation and of the literature. Citing here a few references 20-25 years old relying on a single mitochondrial marker with a few taxa is not a good represetation of the state-of-the-art of Blattodea phylogenetics. This cannot be published as is.

**Response:**

Thanks for your advice. We rewrote this part and cited the recent references. We changed “Although about 4,000 species of cockroaches have been recorded, the phylogenetic relationships within the Blattodea have been debated. Blaberoidea was comprised of Blaberidae and Ectobiidae. The relationships within Blattodea, between Blaberoidea and the remaining cockroaches, have been disputed. Most researchers have supported Blaberoidea being clustered with the remaining cockroaches, while some studies supported Blaberoidea as the sister group to Blattidae. In our study, all trees strongly supported Blaberoidea being placed in the basal position of Blattodea. In addition, within Blaberoidea, we found Ectobiidae was paraphyletic while Blaberoidea was monophyletic (Fig 4 and S3 Fig), consistent with several previous studies. However, more Ectobiidae samples needed to be added in the future to confirm our finding.” to “Although about 4,000 species of cockroaches had been recorded, the relationships within Blattodea had been debated. Blattodea can be divided into three superfamilies: Blaberoidea, Blattoidea and Corydioidea. Many studies supported Blaberoidea being clustered with the Blattoidea+Corydioidea using both morphological and molecular characters , transcriptomes, gene locis, but some studies supported Blaberoidea+Corydiidae as the sister group to the remaining [cockroach](D:/Users/Administrator/AppData/Local/youdao/dict/Application/8.9.6.0/resultui/html/index.html#/javascript:;)es, such as Lo et al (used combined dataset of 18S rRNA, histone 3 and COII), Lo et al (based on 18S rDNA and COII). In our study, we got different results. The clade ((Ectobiidae + Blaberidae)+Corydiidae) was clustered with remaining [cockroach](D:/Users/Administrator/AppData/Local/youdao/dict/Application/8.9.6.0/resultui/html/index.html#/javascript:;)es based on lrRNA+srRNA in the BI analysis while other trees strongly supported Blaberoidea as the sister group to the clade Blaberidae+Corydiidae+Blattidae+Crypyocercidae+Isoptera. Blaberoidea is comprised of Blaberidae and Ectobiidae. In this study, within Blaberoidea, we found Ectobiidae was paraphyletic while Blaberoidea was monophyletic (Figs 4 and S3), consistent with several previous studies”.

41.Page 24, line 462. Well other studies with a more extensive taxonomic sampling already confirm the paraphyly of Ectobiidae (see for instance Evangelista et al. 2021 for I guess the latest study with several tens of nuclear loci)!

**Response:**

Thanks for your advice. We revised our manuscript and changed “However, more Ectobiidae samples needed to be added in the future to confirm our finding” to “In this study, within Blaberoidea, we found Ectobiidae was paraphyletic while Blaberoidea was monophyletic (Figs 4 and S3), consistent with several previous studies”.

42.Page 24, line 463. I do not understand how this can be the focus of a discussion section while you bring new data for an uncontroversial Ectobiidae species? In addition, the most recent studies do not show such huge disagreements as you suggest.

**Response:**

The position of Corydiidae was [controversial](D:/Users/Administrator/AppData/Local/youdao/dict/Application/8.9.6.0/resultui/html/index.html#/javascript:;) and it had been debated for many years. Beside, we obtained different topologies in different trees. We supported this was a valuable discussion point.

43.I wonder whether it really makes sense at all. Aligning mitochondrial sequences with Numts (that are therefore not homologous) and then try to estimate divergence ages does not make sense according to me.

**Response:**

Numts have a low rate of evolution, and they can be used to solve problems in phylogeny, such as Zischler et al(1995) used the Numt of D-loop as phylogenetic outgroup to prove the origin of man, Lou et al(2013) used 2 Numts as “fossil” markers to trace back the speciation time of two sibling fig wasp species. So we [consider](D:/Users/Administrator/AppData/Local/youdao/dict/Application/8.9.6.0/resultui/html/index.html#/javascript:;) their research is meaningful.

**Reviewer 2:**

This paper adds significant new data and knowledge to Blattodea mitochondrial genome. This paper deserves publication, but there are several issues that need to be addressed prior to publication, mainly in the phylogenetic study and fossil selection. Please see the attachment for details.

**Response:**

Thank you for your careful reading of our manuscript. We have carefully read and considered your comments, and we believe that the revised manuscript has been greatly improved in comparison to the previous submission thanks to the critical comments received. The following are point by point responses to your comments:

1. Page 2, line 32. This is not first time report of mt genome rearrangement of Orthopterodea, such as Cryptocercus in Blattodea.

**Response:**

Thanks for your suggestion. What we emphasized here was tRNA deletions in mt genome instead of mt genome rearrangement in Orthopterodea.

2. Page 2, line 38. Delete and rewrite. The authors should highlight the two NUMTS not the phylogenetic relationships within Blattodea.

**Response:**

Thanks for your suggestion. We revised this part and reduced the descriptions of phylogenetic relationships within Blattodea. We changed “The phylogenetic relationships were analyzed based on the amino acid and nucleotide sequences of 13 PCGs using maximum likelihood and Bayesian inference and a similar topology was yielded, except for the position of Caelifera and Eupolyphaga sinensis (Corydiidae). Tree results supported that (Blattidae + (Corydiidae + (Cryptocercidae + Isoptera))) and Blaberoidea being placed in the basal position of Blattodea, Isoptera should be classified as a family within Blattodea and Mantodea as the sister clade to (Isoptera + Blattodea)” to “The phylogenetic results with different datasets and inference methods showed different topologies. We found ((Ectobiidae + Blaberidae)+Corydiidae) was the sister group of the remaining [cockroach](D:/Users/Administrator/AppData/Local/youdao/dict/Application/8.9.6.0/resultui/html/index.html#/javascript:;)es in Blattodea based on lrRNA+srRNA in the BI analysis while other trees strongly supported Blaberoidea as the sister group to the clade Blaberidae + Corydiidae + Blattidae + Crypyocercidae + Isoptera. Besides, the position of Corydiidae was uncertain”.

3. Page 3, line 58. When it comes to exceptions, why not mention gene rearrangement in many insect orders, mt genome fragmentation in Anoplura and so on.

**Response:**

Thank you for your careful work. We had made additions to the manuscript and [supplement](D:/Users/Administrator/AppData/Local/youdao/dict/Application/8.9.6.0/resultui/html/index.html#/javascript:;)ed gene rearrangements.

4. Page 3, line 64. Some.

**Response:**

Thanks for your suggestion and sorry for the careless mistake. Considering the species richness of Blattodea, we had changed “many” to “a few”.

5. Page 3, line 65. The reference here is incomplete.

**Response:**

Thanks for your suggestion and sorry for the careless mistake. We consulted relevant literatures and supplemented it in the manuscript.

6. Delete.

**Response:**

Thank you for your valuable advice. We deleted "interfamily".

7. Page 4, line 68. The authors should list the latest literatures here. Especilally Bourguignon et al., 2018, they used more than 100 mt genomes to reconstruct the phylogeny of Blattodea.

**Response:**

Thanks for your constructive suggestion. We listed the latest literatures here, such as Evangelista et al(2018), Djernæs et al(2015).

8. Page 4, line 77. Citation errors of references.

**Response:**

Thanks for your suggestion and sorry for the careless mistake. We listed Li et al(2020) and Wang et al(2013).

9. What is the purpose of performing phylogenetic trees with mt genomes? These discoveries in your study cannot increase the clarity of Blattodea phylogeny.

**Response:**

In phylogenetic analyses, the absence of some species may lead to [uncertain](D:/Users/Administrator/AppData/Local/youdao/dict/Application/8.9.6.0/resultui/html/index.html#/javascript:;) inferences about phylogenetic relationships (Tomita et al., 2011). Increased samplings can provide more complete and reliable information to perform phylogenetic analysis. In this study, we amplified the entire mt-genome sequence of Episymploce cockroaches for the first time. Performing phylogenetic trees with mt-genomes was used to confirm the taxonomic status of *E.splendens*.

10. Page 5, line 100. Authors should write the specific operation process.

**Response:**

Thanks for your suggestion. We rechecked the part of operation process and more detailed information were added. We [supplement](D:/Users/Administrator/AppData/Local/youdao/dict/Application/8.9.6.0/resultui/html/index.html#/javascript:;)ed “The concentration and purity of gDNA were detected by spectrophotometer. Firstly, 2 μl DNA elution solution was taken to calibrate the instrument. Then, the spot sample hole was wiped clean and 2 μl DNA was taken to determine the concentration and purity of the test sample. In addition, DNA was detected by agarose gel electrophoresis, and 1% agarose gel electrophoresis judged whether DNA was successfully extracted or not. Finally, DNA was stored at -20 ℃” here.

11. Page 10, line 183. Your purpose? The dataset you used here is not clear. 12S,16S,or PCG?

**Response:**

Thank you for your valuable advice. We amplified 2 Numts during PCR in this study. Divergence dating analysis was used to confirm the time of mitochondrial DNA transfer to nuclear DNA. The datasets we used here were aligned sequences, Numt-1 corresponded to partial lrRNA, and Numt-2 was similar to partial lrRNA and its neighboring tRNA-Val.

12. Page 10, line 184. Your aligned sequences used in Beast is not clear. You should demonstrate here instead of in the results part.

**Response:**

Thanks for your constructive suggestion. We moved "There were two Numts found in E. splendens, namely Numt-1 and Numt-2. Through [sequence](D:/Users/Administrator/AppData/Local/youdao/dict/Application/8.9.6.0/resultui/html/index.html#/javascript:;) [alignment](D:/Users/Administrator/AppData/Local/youdao/dict/Application/8.9.6.0/resultui/html/index.html#/javascript:;) by MEGA 5.2, Numt-1 corresponded to partial lrRNA, and Numt-2 was similar to partial lrRNA and its neighboring tRNA-Val of the E. splendens mito-genome" from the results part to the methods part.

13. Page 11, line 101. Please select the right fossils according to Evargelista et al. (2017) and Evangelista et al (2019)

**Response:**

Thank you for your careful work. We read the references carefully and selected new fossils to calculate the divergence time, please see Table 2.

14. Page 9, line 154. This part should be ahead of divergence time analyses.

**Response:**

Thank you for your careful reading of our manuscript. We took the part “Phylogenetic inference” in front of “Divergence dating analysis”.

15. How long are your datasets? And why not use 12S and 16S?

**Response:**

Thank you for your valuable advice. The remaining length of nucleotide data of 13PCGs was 9,923bp, amino acid sequences of 13 PCGs was 10,032bp, and lrRNA+srRNA was 1,375bp. Besides, we added ML and BI trees based on 12S+16S.

16. There's no need to pick up so much termite data. You should choose more data of Ectobiidae instead of the data out of Blattodea.

**Response:**

Thank you for your suggestion. We selected 13 PCGs to performe phylogenetic analysis, but the number of complete mt genomes in Blattodea (expect for termites) especially in Ectobiidae were very [limited](D:/Users/Administrator/AppData/Local/youdao/dict/Application/8.9.6.0/resultui/html/index.html#/javascript:;). We took as many whole mt genomes as we could find in NCBI before, so we did not change the taxas used in this study.

17. Page 9, line 168. It’s not clear that the two datasets correspond to ML and BI models respectively.

**Response:**

Thank you for your careful work. For the 3 datasets, the GTR+I+G model was chosen as the best-fitting model for BI analysis, the GTR model was chosen as the best-fitting model for the ML analysis. We had [revised](D:/Users/Administrator/AppData/Local/youdao/dict/Application/8.9.6.0/resultui/html/index.html#/javascript:;) phylogenetic inference in the methods part and made it more clear and organized.

18. Page 12, line 214. Did you consider the sequencing error? I suggest another way to confirmed the gene spacer.

**Response:**

Thank you for your suggestion. Actually, we designed other primers for confirmation and got the same result (Primers in [the](D:/Users/Administrator/AppData/Local/youdao/dict/Application/8.9.6.0/resultui/html/index.html#/javascript:;) [table](D:/Users/Administrator/AppData/Local/youdao/dict/Application/8.9.6.0/resultui/html/index.html#/javascript:;) [below](D:/Users/Administrator/AppData/Local/youdao/dict/Application/8.9.6.0/resultui/html/index.html#/javascript:;)). Besides, similar situation had been found in *Blaptica dubia* (Cheng et al, 2016). So we thought our judgment was correct.

|  | F | R |
| --- | --- | --- |
| 1 | TATGCTACCTTTGCACGGT | TTATATCCTCCAGTAGATCCTACA |
| 2 | CTACTACCGTATGTTACGACTTA | TGATTATATCCTCCAGTAGATCC |

19. Page 12, line 220. Still need to be confirmed.

**Response:**

Thank you for your suggestion. For this problem, we also designed different primers to confirm our speculation (The primers were listed in the table below). Different primers amplified the same result. Besides, the deletion of tRNAs had been found in other animal mt genomes (San et al., 2004; Peng et al., 2006) before. So we believed our conclusion.

|  | F | R |
| --- | --- | --- |
| 1 | AATAAAGAGTGCCCCTGTC | GATATTACAGGGATGAAGGATA |
| 2 | TAAAGAGTGCCCCTGTC | TGATGAAGCTAAGGCTTGAA |

20. Move to discussion.

**Response:**

Thank you for your suggestion. We deleted “However, the reason for the high content of AT in the mitochondrial genome is still unclear”.

21. Gene names need to be in italics and unified in this paper. For example, both ND1 and NAD1 appeared in this paper.

**Response:**

Thank you for your valuable advice. We unified the gene names and changed the gene names to italics in our manuscript.

22. Delete. Most of the content of your statement in this part does not belong to the results.

**Response:**

Thank you for your careful reading of our manuscript. We remove “The A+T rich region plays an important role in mitochondrial transcription and replication [47] [48]. Since the control region does not code for genes, the degree of variation in the control region is high and the length varies greatly due to its non-coding characteristic [49]. For example, the non-coding region in insects varies from 70 bp in Ruspolia dubia to 9-13 kb in Pissodes spp. [50] [51]” “Repeated units in the control regions were also found in different blattarian insects. However, these repeated sequences had low similarity in various cockroaches and thus may have different origins [4]”.

23. In this part, there is no new discovery based on your data. Although there are some difference with the previous study , I think it is because the different datasets were used.

**Response:**

Thank you for your suggestion. Phylogenetic analysis was used to confirm the taxonomic status of *E.splendens*.

24. Page 16, line 281. Write directly. Foe example, -value (BSP=@@, Fig. 4).

**Response:**

Thank you for your suggestion. We standardized writing in the manuscript, please see line 281, line 282 and line 284.

25. The name is inappropriate and doesn’t reflect the divergence time analyses.

**Response:**

Thank you for your suggestion. We changed “Numts” to “Numts and its divergence time”.

26. Still, it did not belong to the Results. References 54-56 are very old, please cite the lateset one.

**Response:**

Thank you for your suggestion. We deleted “Numts have been found in the nuclear genomes of various eukaryotes and can be used as outgroups of phylogeny to solve phylogenetic problems [54] [55] [56]. The two Numts found in this study could be useful tools for the study of [cockroach phylogenetic](https://xueshu.baidu.com/usercenter/paper/show?paperid=6397dc0ade12b53e101421d4c1c1823a&site=xueshu_se) relationships in the future”.

27. This is already a fact in many previous studies (2007-2021).

**Response:**

Thank you for your suggestion. We deleted “The Blattodea, Isoptera and Mantodea all belonged to Dictyoptera based on morphological and anatomical characteristics [11] [12]. According to molecular and morphology data, Mantodea was considered as the basal split within Dictyoptera [81] [82]. However, DeSalle et al [83] supported that Mantodea and Isoptera were sister groups within Dictyoptera. Nevertheless, more recent studies discovered that Mantodea was the sister clade to (Isoptera + Blattodea), and even indicated that Isoptera should be classified as a family (Termitidae) within Blattodea [34] [39] [84]. In our study, all results supported Mantodea was a sister clade to Blattodea (including Blattodea and Isoptera) with high support. In addition, we found Isoptera clustered into a [monophyletic](C:/Users/Administrator/AppData/Local/youdao/DictBeta/Application/8.9.3.0/resultui/html/index.html#/javascript:;) clade with high support (Fig 4 and S3 Fig), and this was also supported by several previous studies [9] [12] [85]. Consequently, we propose that Isoptera should be classified as a family within Blattodea”.

28. Page 23, line 446. Reference is not correct. Djernæs, 2018.

**Response:**

Thanks for your suggestion and sorry for the careless mistake. We had changed the right reference in the manuscript.

29. Page 23, line 447. Blattodea can be divided into three superfamilies: Blaberidae, Blattoidea and Corydioidea.

**Response:**

Thank you for your suggestion. We added "Blattodea can be divided into three superfamilies: Blaberidae, Blattoidea and Corydioidea" here.

30. Page 24. The author should refer to the latest literature before revising this part.

**Response:**

Thank you for your suggestion. We revised the phylogenetic analysis part and listed the latest literatures.

31. Page 24,25. When it comes to phylogenetic relationships, the author ignores the recent literatures. such as Wang et al., 2017; Bourguignon et al., 2018; Evangelista et al., 2019; Djernæs, et al.,2020 and so on.

Please rewrite it.

**Response:**

Thanks for your advice We modified the references and cited the recent literatures.
