# Supplementary material for: Complete mitochondrial genome of Episymploce splendens (Blattodea: Ectobiidae): A large intergenic spacer and lacking of two tRNA genes
Source: PLoS One. 2022 Jun 2;17(6):e0268064. doi: 10.1371/journal.pone.0268064 (PMC9162313; doi:10.1371/journal.pone.0268064)
Supplement: Attachment — Submitted filename: Response to Reviewers.docx [file pone.0268064.s012.docx]

**Response to Reviewers**

Dear editor:

Thank you for giving us the opportunity to revise our manuscript entitled “Complete mitochondrial genome of *Episymploce splendens* (Blattodea: Ectobiidae): a large intergenic spacer and lacking of two tRNA genes” to PLOS ONE. We appreciate that the reviewers′ valuable comments for our manuscript. These comments are very important for improving our manuscript. We have revised our manuscript according to the reviewers′ comments and also made other improvement for our manuscript. We do hope that our manuscript is appropriate and could be considered for publication in this journal.

Best regards,

Sincerely yours,

Xiuyue Zhang, PhD and Professor

College of Life Sciences, Sichuan University

Email: zhangxy317@126.com

**Reviewers' comments:**

**Reviewer 2:**

This round you responded all the comments carefully, and deleted the meaningless or not closely related part. it feels much more compact and focused. I suggest emphasizing the existence of pseudogenes (numts) and the time when pseudogenes appear in this cockroach, instead of a large intergenic spacer. And I think the introduction to the study of the cockroach mitochondrial genome is too simplistic.

**Response:**

Thanks for your suggestion. The mito-genome commonly displays exceptional economy of organization and the large intergenic spacer was uncommon. We believed the find of large intergenic spacer in mitochondrial genome of *Episymploce splendens* was important for the study of insect mitochondrial genome evolution in the future and so we retained it. And based on your suggests, we also further emphasized the discovery of numts and their significance, and added a description of the mitochondrial genome and enriched the content.

**Reviewer 3:**

1.I failed to remember that I have reviewed this paper before. I failed to find this mt genome of OK094023 in NCBI. I need this sequence to judge the mt genome right or not! If I reviewed it before, I must be concerned on this question. I did not believe two trnQ and trnM lost in Episymploce splendens.

The two trnQ f and trnM can be translated into between two CRs. As the authors said that this is the first report of the tRNAs deletion in Orthopteroidea mito-genomes. But I did not believe it. I suggest the authors check it carefully using the other species in genus Episymploce. Because if the mt genome is existed two similar CRs, the lost genes will be found between two CRs.

I suggest the authors should be carefully checked again.

When the authors used a pair of primers of "Es11 13822-CAGATTATATTGATTCGCACAAC" and "303-ATAGAACTGATGAAGCTAAGGC" to amplify PCR using 75 second Extension. It may be failed to amplify two control regions. I suggest the authors can design more primers to check it.

**Response:**

Thanks for your careful reading.

We really appreciate your valuable time on reviewing our manuscript. We had provided the complete mt-genome to Genbank and obtained the GenBank identifier (OK094023) but we set up the release date, and so this sequence cannot be found in NCBI at present. However, in order to the convenience of review, it has been uploaded as an attachment in our submitted manuscript and also in our revised manuscript.

The non-coding region of *E. splendens* mito-genome was located between srRNA and tRNA-Ile, and it was 290 bp long [consist](D:/Users/Administrator/AppData/Local/youdao/dict/Application/8.9.6.0/resultui/html/index.html#/javascript:;) [of](D:/Users/Administrator/AppData/Local/youdao/dict/Application/8.9.6.0/resultui/html/index.html#/javascript:;) two 120 bp long repeated units. The two lost tRNAs were located behind tRNA-Ile, and the interval between tRNA-Ile and NAD2 was only 24bp. They cannot form the 2 tRNAs. Because we used ARWEN and tRNA-SE to identify tRNAs and obtained same result through reference to secondary structure models for those genes from other blattarian insects (Ma et al., 2017; Xiao et al., 2012). The tRNA-Gln and tRNA-Met located between tRNA-Ile and NAD2, and they were typically 150bp long in other [cockroach](D:/Users/Administrator/AppData/Local/youdao/dict/Application/8.9.6.0/resultui/html/index.html#/javascript:;)es. However, this site was only 24bp long in *E. splendens*. In addition, we tried to refer to other cockroach mitochondrial genomes, but however, there was no mitochondrial sequences reported in *Episymploce* in NCBI. In reported cockroach mitochondrial genomes, we did not find two similar CRs, namely, these reported cockroach mitochondrial genomes have only one A-T region. We designed different primers (in the table) to amplify this area and obtain same amplification sequences, so we think our experiment result was correct and there was no sequence lost due to [inappropriate](javascript:;) primers. We could not found the secondary structures of two tRNAs in *E. splendens* mito-genome sequence, and so we believed their loss event. Although tRNA lacking was rare, other species had been found in previous studies. For example, *Limnonectes bannaensis* mt-genome lost 4 tRNAs (tRNA-Ala, tRNA-Asn, tRNA-Cys and tRNA-Glu), *Gegeneophis ramaswamii* mt-genome lost tRNA-Phe, *Urochela quadrinotata* Reuter mt-genome lost 2 tRNAs (tRNA-Ile and tRNA-Gln), *Platysternon megacephalum* mt-genome lost tRNA-Thr. These loss events of tRNAs occur accidentally and independently in these mt-genome, and we also believe that these findings will be useful for future studies on the cause of their loss and the subsequent adaptive evolution.

|  | F | R |
| --- | --- | --- |
| 1.ES11 | CAGATTATATTGATTCGCACAAC | ATAGAACTGATGAAGCTAAGGC |
| 2.(Ma et al,2017) | CCTCTAAAAAGACTAAAATACCGCC | GGAATCATCAGTGAAAGGGAGC |
| 3. | AATAAAGAGTGCCCCTGTC | GATATTACAGGGATGAAGGATA |
| 4. | TAAAGAGTGCCCCTGTC | TGATGAAGCTAAGGCTTGAA |

2. Two Numts were found by PCR. Two Numts were similar to lrRNA, which can be explained why the author failed to obtained trnQ and trnM. Check the mt genome again!

Another question is how the authors judge the two Numts？ Why is not lrRNA belonging to Numt？

I did think the divergence time of Numts are interesting. Because the title is "Complete mitochondrial genome of Episymploce splendens (Blattodea: Ectobiidae): a large intergenic spacer and lacking of two tRNA genes" not including Numts.

**Response:**

The sequences which originated from the invasion of mtDNA into nuclear DNA were named Numts. Numts exist in the nuclear DNA and do not belong to the mitochondrial genome sequence, but due to their partial sequences may be identical to their source sequences, some primers that amplify mitochondrial genomes may accidentally amplify these sequences. So, two Numts may be not related to the loss of tRNAs.

Primers that amplified mitochondrial genes may occasionally amplify Numts. In this study, agarose gel electrophoresis of the amplification product (the primer Numt-1-1 and Numt-2-1, Table 1) appeared as two bands. We then redesigned primers (Numt-1-2 and Numt-2-2, Table 1) to amplify these regions and obtained the same result. These non-targeted bands were purified and sequenced, and obtained sequences belonging to mitochondria via the analysis of NCBI blast. Due to differences in mutation rates or relaxation from selection pressure, they had some degree of base differences compared to corresponding mito-genome sequences. These sequences have more mutations of insertion and deletion compared to their corresponding mitochondrial sequences, and may lose their functions. They did not belong to the mitochondrial genome, so they were not the lost tRNAs. We had explained this in the corresponding section of the manuscript (Page line 162). Numts can be used to solve problems in phylogeny, but they were not a part of the mitochondria, so we did not mentioned them in the title.

3.Is a large intergenic spacer similar to trnI or trnQ? or AT-rich region?

**Response:**

The large intergenic spacer was not A-T region or similar to trnI or trnQ. It was similar to its [corresponding](C:/Users/Administrator/AppData/Local/youdao/DictBeta/Application/8.9.3.0/resultui/html/index.html#/javascript:;) srRNA with a 64.6% similarity.

4.I read the paper and found the English writing is really poor. There are many mistaken in the paper. I can not believe the paper is revsied the second version.

**Response:**

we're sorry for the writing mistaken and we have made careful revisions to our English writing.

5.All genes name should be the same in text, figures, and tables.

**Response:**

We have made relevant changes in our revised manuscript.
