# Supplementary material for: Complete mitochondrial genome of Episymploce splendens (Blattodea: Ectobiidae): A large intergenic spacer and lacking of two tRNA genes
Source: PLoS One. 2022 Jun 2;17(6):e0268064. doi: 10.1371/journal.pone.0268064 (PMC9162313; doi:10.1371/journal.pone.0268064)
Supplement: Attachment — Submitted filename: Response to Reviewers.docx [file pone.0268064.s014.docx]

**Response to Reviewers**

Dear editor:

Thank you for giving us the opportunity to revise our manuscript entitled “Complete mitochondrial genome of *Episymploce splendens* (Blattodea: Ectobiidae): a large intergenic spacer and lacking of two tRNA genes” to PLOS ONE. We appreciate that the reviewers′ valuable comments for our manuscript. These comments are very important for improving our manuscript. We have revised our manuscript according to the reviewers′ comments and also made other improvement for our manuscript. We do hope that our manuscript is appropriate and could be considered for publication in this journal.

Best regards,

Sincerely yours,

Xiuyue Zhang, PhD and Professor

College of Life Sciences, Sichuan University

Email: zhangxy317@126.com

**Reviewers' comments:**

**Reviewer 4:**

1.This manuscript described the molecular features of the mtDNA of the blattarian species Episymploce splendens, with major emphasis to the presumed lack of two tRNA encoding genes, the occurrence of intergenic spacers and on the occurrence of presumed Numts.

Central to the processed analyses is the impossibility to identify, along the mtDNA, genes encoding trnM and trnQ. It is quite difficult to understand if unsuccessful scanning for tRNA genes is due to the inability of automated programs to detect sequences of abnormal size and structure. Some arthropods species have highly modified tRNA secondary structures and their occurrence within a mtDNA may be nightmare to be assessed. In some instances, genes encoding for tRNAs are embedded within both AT-rich region and PCG encoding genes. I understand Authors have scanned manually for their occurrence and trust them when they reach to the conclusion that every attempt was unsuccessful. However, I suggest to further investigate the occurrence of the missing tRNAs within the coding sequence of adjoining genes (i.e. partially overlapping with tRNAI and with nad2). This would possibly be done by considering every possible trinucleotide primary sequence, corresponding to the anticodon of the missing tRNAs, in the vicinity of the site where these latter are usually found. One more additional analysis should be addressed using relative taxa (other species of the same genus). There is a chance that sequencing the same DNA fragment in closely related taxa would support the presumed lack of the tRNA genes, as well. I understand this would need further analyses but would also provide a more convincing support to the conclusions.

Another result of Authors’s study was the presumed discovery of mitochondrial sequences possibly inserted within the nuclear genome. I’m more prone to suspect that this may be due to some artifact of the sequencing procedure, although I don’t have access to supplementary files S3 and S4. I would rather write a more prudential conclusion: the occurrence of Numts is not clearly assessed, although suspected.

Other minor correction along the text.

In conclusion, the lack of tRNA encoding genes should be further investigated and the occurrence of Numts should be downscaled to a possibility, not stated evidence. These before the manuscript should be considered for publication.

**Response:**

We really appreciate your valuable time on reviewing our manuscript. We have tried to investigate the occurrence of the missing tRNAs within the coding sequence of adjoining genes based on your good comments and don’t have found the missing tRNAs.

The tRNA-Gln and tRNA-Met located between tRNA-Ile and NAD2, and they were typically 150bp long in other blattarian insects. However, the interval between tRNA-Ile and NAD2 in *E. splendens* mit-genome was only 24bp and cannot form the 2 tRNAs. We designed different primers to amplify this area and obtain same amplification sequences, so we think our experiment result was correct and there was no sequence lost due to [inappropriate](javascript:;) primers. Because we used ARWEN and tRNA-SE to identify tRNAs and obtained same result through reference to secondary structure models for those genes from other blattarian insects (Ma et al., 2017; Xiao et al., 2012).

Based on your suggestion, we extended the 24bp sequence between tRNA-ILE and NAD2 about 70bp into tRNA-ILE and NAD2, respectively. Then use the online server ARWEN (http://mbio-serv2.mbioekol.lu.se/ARWEN/) and tRNA-SE to identify tRNAs, and we found no tRNAs (as the following Figure 1).

Figure 1 Result in ARWEN

Using similar sequence interception methods, we mapped RNA structures using RNAfold (an online tool for mapping RNA structures) with tRNA-Gln and tRNA-Met anticodons as the centre, and found no typical tRNAs. The partial results are shown in the following Figure 2.

Figure 2 Part of the result in RNAfold（http://rna.tbi.univie.ac.at//cgi-bin/RNAWebSuite/RNAfold.cgi）

We did also the similar analysis with AT-rich region and other PCG encoding genes, and didn’t find the absent tRNAs. So, we believed the loss event dose exist in *E. splendens* mito-genome.

Only *Episymploce splendens* mit-genome has been sequenced in the Episymploce (our study), so we can’t do additional analysis within *Episymploce*. It is a pity that we don't have samples of other species of this genus, so we can't sequence the same fragments from other species and can’t know if there are similar tRNAs loss in other species. Although related species may have similar events, some previous studies also showed that the loss of tRNAs occurs accidentally or independently in different species (Shi et al, 2021). Therefore, Further studies are needed in the future to confirm whether other species of Episymploce also have tRNAs loss. Anyway, 2 species of Blattellidae (EU854321 and JX233805) have been sequenced, and neither of them has any tRNA lossing.

Indeed, sequencing, especially poor sequencing, can lead to artifacts of Numts, but our sequencing results were very good (As the sequencing peaks of Numts shown in following Figure 3, 4). We used forward and reverse bidirectional sequencing, and removed the inaccurate sequence at the beginning and end of sequences, and finally obtained the correct spliced sequences. And we also used different primer pairs to perform PCR and sequence, and we obtained the same results, so we believed that our results are correct. These Numts have more mutations of insertion and deletion compared to their corresponding mitochondrial sequences due to differences in mutation rates or relaxation from selection pressure. The supplementary files S3 and S4 showing the sequence alignment of Numts with their corresponding mtDNA were shown in following Figure 5,6.

Figure 3 The sequencing peaks of Numt-1

Figure 4 The sequencing peaks of Numt-2

Figure 5 Fig S3 Sequence alignment of Numt-1 and the corresponding mtDNA

Figure 6 Fig S4 Sequence alignment of Numt-2 and the corresponding mtDNA

2. Page 2, line 32. Here and along the text... blattarian not capital letter

**Response:**

Thanks for your suggestion and sorry for the careless mistake. We have checked the text and corrected the mistake capital letter.

3. Page 3, line 56-58. rewrite sentence for clarity

**Response:**

Thanks for your suggestion. We changed “The tRNAs are most various in no-typical insect mito-genomes” to “The changes of tRNAs are most various in no-typical insect mito-genomes”.

4. Page 3, line 58. obvious

**Response:**

Phthiraptera and Psocoptera insect mito-genomes may have the most changes, that they could fission into fragmented mito-genomes and have numerous pseudo-genes and diverse gene rearrangements (Simon et al, 2013; Chen et al, 2016; Shi et al, 2021; Shi et al, 2016).

5. Page 3, line 59. with respect to?

**Response:**

Thanks for your suggestion. Their fast evolution were shown in Page 3, line 61-62 “that they could fission into fragmented mito-genomes and have numerous pseudo-genes and diverse gene rearrangements”(Simon et al, 2013; Chen et al, 2016; Shi et al, 2021; Shi et al, 2016).

6. Page 3, line 61. s

**Response:**

Thanks for your suggestion and sorry for the careless mistake. We have changed “rearrangement” to “rearrangements”.

7. Page 3, line 66. what is a "conserved evolution"?

**Response:**

We has changed “The blattarian mito-genomes seem to be in a conserved evolution”. to “The blattarian mito-genomes seem to be conserved in evolution”.

8. Page 4, line 77. what are the open questions and aims addressed by Authors? Please add aims

**Response:**

Thanks for your suggestion. We have changed “*Episymploce splendens* belongs to Ectobiidae and at present, no complete mitochondrial sequences of *Episymploce* have been recorded in the NCBI database. Previous studies have mainly focused on the morphological features of *Episymploce* rather than molecular data [18-19]. In this study, we sequenced ” to “*Episymploce splendens* belongs to Ectobiidae and Previous studies have mainly focused on the morphological features of *Episymploce* rather than molecular data [18-19]. At present, no complete mitochondrial sequences of *Episymploce* have been recorded in the NCBI database. To obtain the sequence information and organization features of mito-genomes in *Episymploce*, we sequenced”.

9. Page 4, line 81-86. this is not M&M topic

**Response:**

Thank you for your valuable advice. We have deleted the irrelevant content.

10. Page 5, line 92-95. delete

**Response:**

Thank you for your suggestion. We have deleted this part.

11. Page 8, line117. "invasion" is a term used for transposons

**Response:**

Thank you for your suggestion. We have changed this sentence to “The sequences which were transferred to nuclear DNA from mitochondrial genome were named Numts”.

12. Page 8, line 137. checked

**Response:**

Thanks for your suggestion. We have changed “recognized” to “checked”.

13. Page 10, line 169. no genome codes for genes, but rather for proteins of other transcripts

**Response:**

Thanks for your advice. We have changed “It coded for 35 mitochondrial genes:” to “It contained 35 mitochondrial genes:”

14. Page 11, line 183. usually

**Response:**

Thanks for your advice. We have added “usually” after “tRNAs”.

15. Page 14, line 238. what is a "low rate of evolution"?

**Response:**

.We have changed this sentence to “They have different mutation rates compared to their ancient mtDNA”.

Thank you again for your careful revision and good suggestions for our manuscript.
